# A Systematic Review of the Methods Used to Assess and Report Dietary Patterns

**DOI:** 10.3389/fnut.2022.892351

**Published:** 2022-05-25

**Authors:** Kate Wingrove, Mark A. Lawrence, Sarah A. McNaughton

**Affiliations:** Institute for Physical Activity and Nutrition (IPAN), School of Exercise and Nutrition Sciences, Deakin University, Geelong, VIC, Australia

**Keywords:** dietary patterns, dietary guidelines, evidence synthesis, evidence translation, systematic review

## Abstract

The use of dietary pattern assessment methods has increased over time. However, data from individual studies can be difficult to compare and synthesize when the dietary pattern assessment methods, and the dietary patterns that are identified are not described sufficiently. The aims of this systematic review were to analyze the application and reporting of dietary pattern assessment methods, and the reporting of the dietary patterns that were analyzed with health outcomes. Three electronic databases were searched (Medline, Embase, and Global Health). Cohort and nested case control studies published in English between January 1980 and March 2019 that examined associations between dietary patterns and health outcomes (including cardiovascular disease, cancer, diabetes and metabolic syndrome, and body weight) in apparently healthy, community dwelling adults (aged over 18 years) or children (aged 2–18 years) were eligible for inclusion. A narrative synthesis was conducted and descriptive statistics were used to summarize the application and reporting of each dietary pattern assessment method, and the reporting of the identified dietary patterns. Analysis of associations between dietary patterns and health outcomes was beyond the scope of this review. Of the included studies *(n* = 410), 62.7% used index-based methods, 30.5% used factor analysis or principal component analysis, 6.3% used reduced rank regression, and 5.6% used cluster analysis. Some studies (4.6%) used more than one method. There was considerable variation in the application and reporting of dietary pattern assessment methods. For example, the application of Mediterranean diet indices varied in terms of the nature of dietary components (foods only or foods and nutrients) and the rationale behind the cut-off points (absolute and/or data driven). In some cases, important methodological details were omitted. The level of detail used to describe the dietary patterns also varied, and food and nutrient profiles were often not reported. To ensure dietary patterns evidence can be synthesized and translated into dietary guidelines, standardized approaches for the application and reporting of dietary pattern assessment methods and the reporting of the identified dietary patterns would be beneficial.

## Introduction

Dietary patterns are complex exposures that incorporate the quantities and combinations of foods that are habitually consumed, the nutrients and those foods contain, and the interactions between dietary components (1–3). Dietary patterns research first appeared in the nutrition science literature in the 1980s, and the methods used to assess dietary patterns have evolved over time (2, 4). Dietary pattern assessment methods can be broadly classified as index-based or data driven. Index-based methods (also known as “investigator driven” or “*a priori*” methods) are used to measure adherence to dietary patterns that have been predefined based on prior knowledge of associations between diet and health (2, 4, 5). Data driven (or “*a posteriori”*) methods use multivariate statistical techniques, such as factor analysis or principal component analysis (FA/PCA), reduced rank regression (RRR), and cluster analysis (CA) to derive patterns from dietary intake data (2, 4, 5).

The application of dietary pattern assessment methods requires researchers to make subjective decisions that could influence results (2, 4, 6). For example, index-based methods require decisions to be made about the number and nature of dietary components and the cut-off points for scoring (7–10), and data driven methods require decisions about the number of food groups that are entered into the dietary pattern analysis, and the number of dietary patterns that are retained for analysis with health outcomes (7, 11–13). If the application of the methods is not reported in sufficient detail, strengths and limitations associated with the methods cannot be accurately assessed, and results from individual studies are difficult to compare and synthesize (14–16).

Some attempts to inform standardized approaches for the application of dietary pattern assessment methods have been documented. For example, to inform the 2015 Dietary Guidelines for Americans, the Dietary Patterns Methods Project aimed to identify associations between dietary patterns and mortality in the United States by applying standardized methods to datasets from three large prospective cohort studies (17). Four diet quality indices were applied: the Healthy Eating Index 2010 (HEI-2010); the Alternative Healthy Eating Index 2010 (AHEI-2010); the Alternate Mediterranean Diet Score (aMED); and the Dietary Approaches to Stop Hypertension (DASH) Score. Application of each method was standardized in terms of the approaches used to code dietary intake data and the criteria used to determine cut-off points for scoring (17). A higher quality diet was consistently and significantly associated with reduced risk of all-cause mortality, cardiovascular disease mortality, and cancer mortality (17–20). The results of this project demonstrate the potential for dietary patterns studies to provide consistent evidence when dietary pattern assessment methods are applied in a standardized way.

The Dietary Patterns Methods Project examined the application of index-based methods, which in theory, can be used to assess adherence to predefined dietary patterns across different study populations (2, 17). In contrast, dietary patterns that are derived using data driven methods are specific to the study population (2, 11). A systematic review published in 2018 compared the application of data driven methods in six studies investigating associations between dietary patterns and health outcomes in a European context (11). In four out of the five studies that used FA/PCA, there was variation in the rationale used to determine the number of dietary patterns to retain, and in the remaining study, this information was not reported (11). In the two studies that used CA, the same statistical method to identify clusters was reported, but the method was applied using different criteria (11). The results of this systematic review highlight the need for more consistency in the application and reporting of data driven methods.

The level of detail used to describe the dietary patterns that are analyzed with health outcomes can influence the extent to which results of individual studies can be compared and synthesized (15, 21). For example, for studies that use index-based methods, people with similar dietary pattern scores may have consumed very different dietary patterns (15, 21, 22). For studies that use data driven methods, decisions about the criteria used to name the dietary patterns that are retained can influence comparability of results (11, 12). For example, the composition of a “Western” dietary pattern identified in one study could differ substantially from a dietary pattern with the same name that was identified in another study. Results of individual studies may be easier to compare and synthesize when the dietary patterns that are analyzed with health outcomes are described using quantitative information about the foods and nutrients they contain (15, 21).

It is now expected that dietary guidelines are informed by evidence from dietary patterns research (23–25). However, a lack of standardization in the application and reporting of dietary pattern assessment methods, and the reporting of the dietary patterns that are analyzed with health outcomes can make it difficult to synthesize evidence from dietary patterns research (7, 14, 15, 22). These evidence synthesis challenges may limit the translation of evidence from dietary patterns research into dietary guidelines (2, 17, 23, 26). The aims of this systematic review were to analyze the application and reporting of dietary pattern assessment methods, and the reporting of the dietary patterns that were analyzed with health outcomes. This review builds on recently published systematic reviews of index-based (21, 27) and data driven (11, 28) dietary pattern assessment methods by analyzing data from a large number of studies that investigated associations between dietary patterns (derived using index-based or data driven methods) and a range of health outcomes that are relevant in the context of dietary guideline development.

## Methods

This systematic review was conducted alongside a systematic review of dietary patterns and health outcomes commissioned by the World Health Organization (WHO) to inform dietary guidelines (29, 30). Observational studies included in the review of dietary patterns and health outcomes were analyzed in this review of dietary pattern assessment methods and reporting. This systematic review has been reported according to the Preferred Reporting Items for Systematic reviews and Meta-Analyses (PRISMA) 2020 Statement (31). This review was not registered. The protocol is available on request.

### Eligibility Criteria

Studies that included apparently healthy, community dwelling adults (aged over 18 years) and children (aged 2–18 years) were eligible for inclusion in this systematic review. Studies conducted in specialist populations (e.g., elite athletes) and institutionalized populations were excluded, as were studies conducted exclusively in pregnant women, diseased populations and acutely ill populations. Studies were included if they examined the association between dietary patterns and the following health outcomes: cardiovascular disease, cancer, diabetes and metabolic syndrome, mortality, body weight, bone health, and micronutrient deficiency among adults, and cardiovascular disease risk (blood lipids and blood pressure), diabetes and metabolic syndrome, growth, body weight, bone health, micronutrient deficiency, and cognition for children. These outcomes were selected using results of a scoping review that was commissioned by the WHO (32), and an outcome prioritization procedure that was conducted in accordance with the WHO Handbook for Guideline Development (29, 33). Studies that did not include any of these outcomes were excluded.

Dietary patterns were the exposure of interest. Conceptually, dietary patterns were defined by the characteristics, quantities, combinations, and frequency of food and beverage consumption (29). Studies were eligible for inclusion if at least one of the following dietary pattern assessment methods were used: index-based methods (defined as methods used to assess adherence to predefined dietary patterns), FA/PCA, RRR, and CA. These methods were selected as they were considered to be the most commonly used dietary pattern assessment methods (2, 4, 5). Studies were only eligible for inclusion if dietary intake was assessed using a food frequency questionnaire, a food diary or food record where data were collected for at least 2 days, or two or more 24-h recalls. These dietary intake assessment methods were selected as they are considered suitable for assessing usual dietary intake (29, 34). Studies that used a single 24-h recall to assess dietary intake were excluded. Studies that assessed combined lifestyle exposures (e.g., dietary patterns combined with physical activity) were also excluded.

Cohort and nested case control studies published in English between January 1980 and March 2019 were eligible for inclusion. The January 1980 start date was selected as this is when dietary patterns research began to appear in the literature (2, 4). Randomized controlled trials were eligible for inclusion in the systematic review of dietary patterns and health outcomes, but intervention studies were excluded from this review. Case control studies, and cross-sectional studies were also excluded.

### Information Sources and Search Strategy

The search strategy was developed in consultation with a subject librarian and informed by the scoping review (29, 32). Three electronic databases (Medline, Embase, and Global Health) were searched in March 2019. Details of the search strategy have been reported elsewhere (29).

### Study Selection

Search results were imported into Covidence systematic review software (35) and duplicates removed. Titles and abstracts were assessed independently by two people, and any discrepancies were resolved by a third person. Full texts were retrieved for the studies that were included at the title and abstract stage, and the assessment process was repeated. Reasons for exclusion were documented at the full text stage.

### Data Collection Process

Data were collected and managed using REDCap electronic data capture tools hosted at Deakin University (36, 37). The data extraction tool was developed using a two-stage approach. Each data extraction item was accompanied by detailed instructions to define the items and ensure consistency across studies. The tool was tested using a 5% sample of included studies. Two authors (KW and SM) extracted data independently. Discrepancies were discussed, and the data extraction tool was updated. Data extraction for all the remaining studies was completed by the first author (KW), with input and guidance provided by a second author (SM). The notes function in REDCap was used to document decision making, and further detail was added to the data extraction instructions as required.

### Data Extraction Items

The data extraction tool was used to collect basic study information (author names, article title, publication year) and detailed information on the application and reporting of dietary pattern assessment methods, and the reporting of the dietary patterns that were analyzed with health outcomes. Data extraction items were intended to reflect the decisions that need to be made by authors when applying dietary pattern assessment methods and reporting the dietary patterns that are identified (7, 11, 12). A combination of fixed and open-ended response options was used to capture the data. Once all data had been extracted, open-ended responses were coded. If the required information was not reported, but a citation was provided in relation to the application of the dietary pattern assessment method “citation provided” was selected. The citation provided may or may not have included the required information. If the required information was not reported and a citation was not provided, “not reported” was selected.

#### Index-Based Methods

For studies that used index-based methods, the number of indices used in the analysis with health outcomes was extracted. If different versions of the same index were used within the same study, they were counted as separate indices. Indices were only counted if they were used in the primary analysis with health outcomes, and if results of the analysis were reported. To analyze the application and reporting of each method, the following data were extracted: the name of the index, whether modifications were made to pre-existing indices, the number of dietary components, the nature of dietary components (e.g., foods only or foods and nutrients), the cut-off points for dietary components (e.g., dichotomous or proportional), the rationale for cut-off points (e.g., absolute or data driven), and the possible score range. Cut-off points were classified as “dichotomous” when only two scoring options existed (e.g., 0 or 1), and “proportional” when more than two scoring options existed (e.g., 0, 1 or 2). The rationale for cut-off points was classified as “absolute” if the cut-off point was fixed (e.g., based on dietary guidelines), and “data driven” if the cut-off off point was specific to the study population (e.g., based on mean or median intake) (7).

To analyze the reporting of the dietary patterns that were analyzed with health outcomes, data were extracted on whether food profiles and nutrient profiles were reported, and whether results were stratified by sex. Food and nutrient profiles were defined as the presentation of quantitative information on intake of at least one food or food group and at least one nutrient from the dietary pattern according to the level of adherence (e.g., the presentation of grams of vegetables consumed by those in the highest quintile compared to the lowest quintile). For studies that only included women or men, stratification by sex was “not applicable.”

#### Data Driven Methods

The following data were extracted to analyze the application and reporting of each data driven method: the number of food groups entered into the dietary pattern analysis, whether the names of all food groups were reported, the criteria used for defining food groups (e.g., to reflect the food frequency questionnaire (FFQ) that was used to collect dietary data, or based on dietary guideline recommendations), the input unit for food groups (e.g., grams or frequency), the rationale for the number of dietary patterns retained (e.g., based on eigenvalues, scree plots, and/or interpretability), whether reliability of the dietary patterns was assessed, the number of dietary patterns retained, and the number of dietary patterns analyzed with health outcomes (where this was different from the number of dietary patterns retained) (11, 12). If dietary intake data were collected using an FFQ, and detail on the criteria for food groups was not provided, it was assumed that the food groups entered into the analysis reflected the FFQ. If an FFQ was used to collect dietary data, and additional criteria were applied, only those additional criteria were selected.

For FA/PCA and RRR, the following data were also extracted: type of energy adjustment method (e.g., using the nutrient density method or the residual method) (38), whether the dietary pattern scores were calculated using all the food groups that were entered into the dietary pattern analysis or only those above a specific factor loading cut-off point, and the total percentage of variation explained by the dietary patterns that were retained. For FA/PCA, data on the type of rotation used (e.g., orthogonal or oblique rotation) was extracted. For RRR, data on the number and nature of intermediate variables (e.g., biomarkers of disease risk or dietary intake) was extracted. For CA, data on the type of cluster analysis were extracted (e.g., k-means or Wards method) (7, 11).

The following data were extracted to analyze the reporting of the dietary patterns that were analyzed with health outcomes: how the dietary patterns were named (e.g., using qualitative labels such as “Western pattern” or “Traditional pattern” or based on the names of the foods such as “Meat and dairy pattern”), whether food profiles and nutrient profiles were reported, and whether results were stratified by sex. For dietary patterns derived using FA/PCA or RRR, the definition of a food profile and a nutrient profile matched the definition used for indices. For dietary patterns derived using CA, dietary intake of at least one food or food group and at least one nutrient had to be reported for each cluster. For studies that only included women or men, stratification by sex was “not applicable.”

### Synthesis of Results

A narrative synthesis was conducted. Analysis of the associations between dietary patterns and health outcomes was beyond the scope of this review, so the risk of bias associated with included studies and the certainty of evidence for each outcome was not assessed. At the study level, descriptive statistics were used to identify the dietary pattern assessment methods that were most frequently used. To examine whether certain methods were more frequently used during particular time periods, studies were also analyzed by publication year.

Descriptive statistics were used to summarize the extracted data relating to the application and reporting of each dietary pattern assessment method, and the reporting of the dietary patterns that were analyzed with health outcomes. For the studies that used index-based methods, each index was treated as the unit of analysis, as the methodological considerations are relevant to each index. For the studies that used data driven methods, each data driven method was treated as the unit of analysis as the methodological considerations are relevant to each method. Subgroup analyses were conducted for indices, so that variation in the application of frequently used indices with similar names could be assessed.

## Results

### Study Selection

**FIGURE 1:**
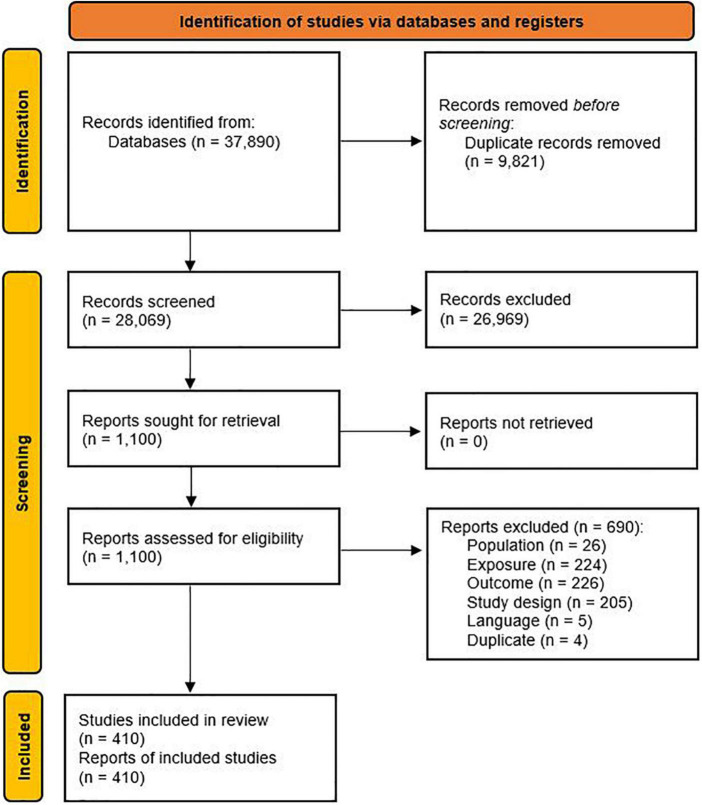
Study selection flow diagram.

A total of 410 studies were included in this systematic review (Figure 1). Each report was treated as a study because the aims of this systematic review were to analyze the application and reporting of dietary pattern assessment methods and the reporting of the dietary patterns that were analyzed with health outcomes, which are factors that reflect the decisions that are made by the authors of each report. An overview of included studies is provided in Supplementary Table 1.

### Study Characteristics

Approximately two thirds of included studies used index-based methods (*n* = 257, 62.7%), approximately one third used FA/PCA (*n* = 125, 30.5%), and a small proportion used RRR (*n* = 26, 6.3%) or CA (*n* = 23, 5.6%) (Table 1). Nineteen studies (4.6%) used more than one dietary pattern assessment method. Fourteen studies (3.4%) used a combination of index-based and data driven methods. Seven studies (1.7%) used multiple data driven methods. Two studies (0.5%) used other data driven methods in addition to FA/PCA, RRR, or CA. One study used random forest with classification tree analysis, and one study used partial least squares regression and principal components regression.

**TABLE 1:** Studies classified according to the dietary pattern assessment methods that were used (*n* = 410).

Dietary pattern assessment method	*n*	% of studies*
Index-based methods	257	62.7
Factor analysis or principal component analysis	125	30.5
Reduced rank regression	26	6.3
Cluster analysis	23	5.6
Other data driven methods	2	0.5

*Some studies used more than one dietary pattern assessment method, so frequencies add up to more than 100%.

**FIGURE 2:**
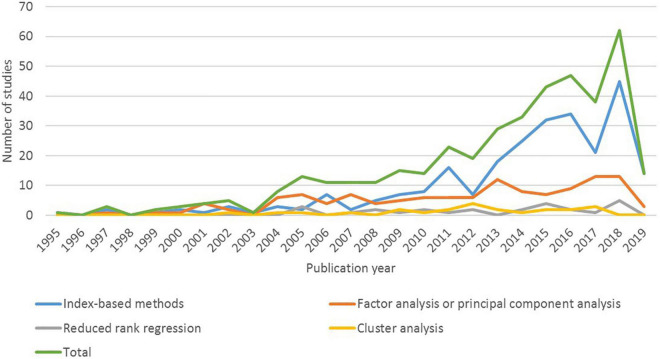
Dietary patterns studies classified according to dietary pattern assessment method and publication year (*n* = 410 studies).

The studies included in this review were published between 1995 and 2019 (the literature search was conducted in March 2019). Figure 2 provides a frequency graph of the total number of studies published over time and the number of studies according to dietary pattern assessment method. This graph demonstrates that the increase in the number of studies published between 2010 and 2019 is largely due to an increase in studies that used index-based methods.

### Index-Based Methods

Of the 257 studies that used index-based methods, most studies used one index only (*n* = 162, 63.0%). The remaining studies used two indices (*n* = 40, 15.6%), three indices (*n* = 21, 8.2%), four indices (*n* = 24, 9.3%), or more than four indices (*n* = 10, 3.9%). The highest number of indices used in any one study was nine.

A total of 463 distinct indices were used (Supplementary Table 2). Based on the name of each index, the most frequently used indices were categorized as Mediterranean diet (MD) indices (*n* = 187, 40.4% of all indices), adaptations of the Healthy Eating Index (HEI) (*n* = 83, 17.9% of all indices), and Dietary Approaches to Stop Hypertension (DASH) indices (*n* = 49, 10.6% of all indices). Indices that were less frequently used were categorized as “other” indices (*n* = 144, 31.1% of all indices). The most frequently used indices within this category were versions of the Healthy Nordic Food Index (*n* = 11, 2.4% of all indices), the Diet Quality Index (*n* = 10, 2.2% of all indices), the WHO Healthy Diet Indicator (*n* = 10, 2.2% of all indices), the Recommended Food Score (*n* = 10, 2.2% of all indices), the Dietary Inflammatory Index (*n* = 6, 1.3% of all indices), and the WCRF/AICR Diet Adherence Score (*n* = 6, 1.3% of all indices).

Approximately one third (32.8%) of all indices were modified versions of existing indices. Modifications were generally used to account for limitations in dietary intake data, or to make the index more applicable to the study population. Compared to MD indices and HEI indices, modified versions of DASH indices were used less frequently (10.2% of DASH indices were modified, compared to 35.8% of MD indices and 27.7% of HEI indices).

Approximately two thirds (64.4%) of all indices included less than 11 dietary components, and around one fifth (21.8%) included between 11 and 20 components. MD indices and DASH indices usually included less than 11 components (77.5% of MD indices and 87.8% of DASH indices), whereas a larger proportion of indices in the HEI category included between 11 and 20 components (47.0% of HEI indices). The only indices that included more than 20 components were in the “other” category.

Approximately two thirds (61.6%) of all indices included a combination of foods and nutrients and one quarter (25.7%) included foods only. Similar frequencies were observed for MD indices. Compared to MD indices, a larger proportion of DASH indices included foods and nutrients (79.6% of DASH indices), and a smaller proportion included foods only (8.2% of DASH indices). Some of the indices in the HEI category included supplements as well as foods (or supplements as well as foods and nutrients) (10.8% of HEI indices), and none of the HEI indices included foods only. Compared to MD indices, HEI indices, and DASH indices, a larger proportion of other indices included foods only (47.2% of other indices).

Many indices used cut-off points that were dichotomous (39.3% of all indices), many used cut-off points that were proportional (35.6% of all indices), and a small proportion used a combination of dichotomous and proportional cut-off points (6.7% of all indices). Most MD indices used dichotomous cut-off points (67.4% of MD indices), whereas most HEI indices and DASH indices used proportional cut-off points (65.1% of HEI indices and 75.5% of DASH indices). Compared to MD indices, a citation was provided more frequently for HEI indices (25.3% of HEI indices) and DASH indices (22.4% of DASH indices).

Approximately one third (31.3%) of all indices used a combination of absolute and data driven cut-off points, some (28.1%) used absolute cut-off points only, and some (21.6%) used data driven cut-off points only. Most MD indices used a combination of absolute and data driven cut-off points (61.0% of MD indices), whereas most HEI indices used absolute cut-off points only (49.4% of HEI indices), and most DASH indices used data driven cut-off points only (77.6% of DASH indices).

For most indices, the possible score range was explicitly stated, or could be derived from the information provided by the authors (90.3% of all indices). For MD indices, the most frequently used score ranges were 0–9 (46.0% of MD indices) and 0–8 (16.0% of MD indices). For HEI indices, the most commonly used score ranges were 0–100 (42.2% of HEI indices) and 0–110 (25.3% of HEI indices). For DASH indices, the most frequently used score range was 8–40 (69.4% of DASH indices).

The following information was omitted more than 10% of the time: the number of dietary components (citation provided for 12.0% of HEI indices and 10.2% of DASH indices), the nature of dietary components (citation provided for 10.2% of MD indices, 15.7% of HEI indices, and 12.2% of DASH indices), the cut-off points for dietary components (citation provided for 17.9% of all indices, 15.0% of MD indices, 25.3% of DASH indices, and 16.0% of other indices), the rationale for cut-off points (citation provided for 17.5% of all indices, 15.5% of MD indices, 22.9% of HEI indices, 14.3% of DASH indices, and 18.1% of other indices), and the possible score range (citation provided for 10.2% of DASH indices and 12.5% of other indices). The citations that were provided may or may not have included the required information.

**FIGURE 3:**
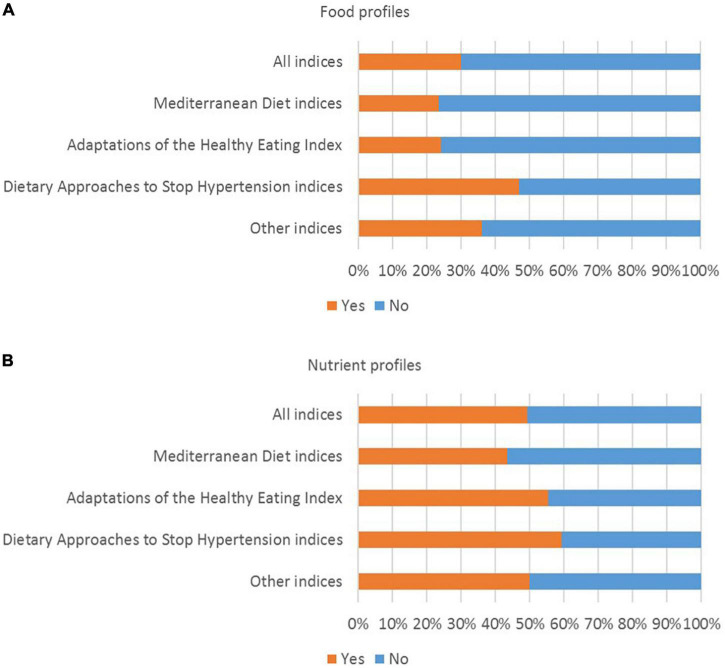
Percentage of indices for which **(A)** food profiles and **(B)** nutrient profiles were reported (*n* = 463 indices).

Food profiles were provided for approximately one third (30.0%) of all indices (Figure 3). Compared to MD indices, HEI indices, and other indices, food profiles were reported more frequently for DASH indices (46.9% of DASH indices). Nutrient profiles were provided for approximately half of all indices (49.2% of all indices). Compared to MD indices, HEI indices, and other indices, nutrient profiles were reported more frequently for DASH indices (59.2% of DASH indices). In the analysis with health outcomes, results for approximately one third (34.6%) of all indices were stratified by sex.

### Data Driven Methods

Considerable variation in the application and reporting of data driven dietary pattern assessment methods was observed (Supplementary Table 3). The most common number of food groups that were entered into the dietary pattern analysis varied according the method that was used (31–40 food groups for FA/PCA and RRR, and more than 40 food groups for CA). Variation in the application of each method was also observed. For example, in the application of FA/PCA, 31–40 food groups were entered in about one third of studies (36.0%), but in many studies 21–30 food groups (21.6%) or more than 40 food groups (26.4%) were entered. The names of all food groups entered into the analysis (including the food groups that may not have contributed to the final dietary pattern score) were reported in approximately 60% of studies (60.8% of FA/PCA studies, 57.7% of RRR studies, and 60.9% of CA studies). A combination of criteria was often used to define the food groups that were entered into the analysis. For example, in studies that used FA/PCA, foods were frequently grouped based on the FFQ (41.6%), based on food composition or type of food (32.8%), and/or based on culinary use (20.8%). Frequency (e.g., servings per day) was the most commonly used input unit in the application of FA/PCA (18.4%), compared to grams for RRR (23.1%), and percentage of energy for CA (34.8%).

In studies that used FA/PCA or RRR, energy intake was frequently adjusted for in statistical models as part of the analysis with health outcomes (49.6% of FA/PCA studies and 46.2% of RRR studies), but in some studies energy adjustment occurred as part of the dietary pattern analysis (prior to the analysis with health outcomes). In studies that used FA/PCA, the residual method was used more frequently than the nutrient density method (22.4 and 8.8%, respectively). In studies that used RRR, the nutrient density method was used more frequently than the residual method (23.1 and 19.2%, respectively). In studies that used FA/PCA, most studies (75.2%) used orthogonal rotation. In most FA/PCA studies (77.6%), the dietary pattern score was calculated using all the food groups that were entered into the dietary pattern analysis. A factor loading cut-off point was sometimes used for descriptive purposes, but only 8.8% of FA/PCA studies calculated the score using only the food groups above a factor loading cut-off point. The cut-off points that were reported ranged from 0.20 to 0.40. In the application of RRR, approximately one quarter of studies (26.9%) used all food groups, one quarter (26.9%) used food groups above a specific cut-off point, and one third (30.8%) used other methods to calculate the dietary pattern score. For the RRR studies that used a cut-off point, 0.20 was consistently used.

In studies that used RRR, the number and nature of intermediate variables varied. Just under half of RRR studies (42.3%) used three intermediate variables, and 19.2% used two intermediate variables. Disease or disease risk biomarkers were used in 65.4% of RRR studies. These included blood lipids, inflammatory biomarkers, hormone levels, glucose markers (e.g., HOMA, HBA1C), and cardiovascular disease risk factors (e.g., BMI, blood pressure). Measures of dietary intake were used as intermediate variables in 34.6% of RRR studies, but none of the studies used biomarkers of nutrient intake. In studies that used CA, approximately half (47.8%) used the k-means method, 17.4% used Ward’s method, and 13.0% used latent class analysis. In approximately half of the studies that used FA/PCA, percentage of variation explained was less than or equal to 20% (25.6%), or between 21 and 30% (24.8%). In studies that used FA/PCA, the number of dietary patterns to retain was often selected based on eigenvalues (72.8%), scree plots (67.2%) and/or interpretability (60.8%). In 53.5% of studies that used RRR, the number of dietary patterns to retain was based on the percentage of variation explained by each intermediate variable. In studies that used CA, the number of clusters to retain was most frequently informed by model fit statistics (39.1%) and/or cluster sample size (26.1%). Reliability of the dietary patterns was assessed in 30.8% of RRR studies, compared to 15.2% of FA/PCA studies and 4.3% of CA studies.

The following information was omitted more than 20% of the time: a complete list of the names of the food groups that were entered into the dietary pattern analysis (citation provided in 29.6% of FA/PCA studies, 38.5% of RRR studies, and 30.4% of CA studies), the criteria used to define the food groups (citation provided in 34.6% of RRR studies and 34.8% of CA studies), the input unit for food groups (citation provided in 31.2% of FA/PCA studies, 38.5% of RRR studies and 21.7% of CA studies; not reported in 31.2% of FA/PCA studies, 11.5% of RRR studies and 13.0% of CA studies), the total percentage of variation explained by the dietary patterns that were derived (citation provided in 24.8% of FA/PCA studies and 38.5% of RRR studies; not reported in 14.4% of FA/PCA studies and 42.3% of RRR studies), the rationale for the number of dietary patterns that were retained (citation provided in 26.9% of RRR studies and 43.5% of CA studies), and the reliability of the dietary patterns (citation provided in 44.0% of FA/PCA studies, 42.3% of RRR studies and 60.9% of CA studies; not reported in 40.8% of FA/PCA studies, 26.9% of RRR studies and 34.8% of CA studies). The citations that were provided may or may not have included the required information.

**FIGURE 4:**
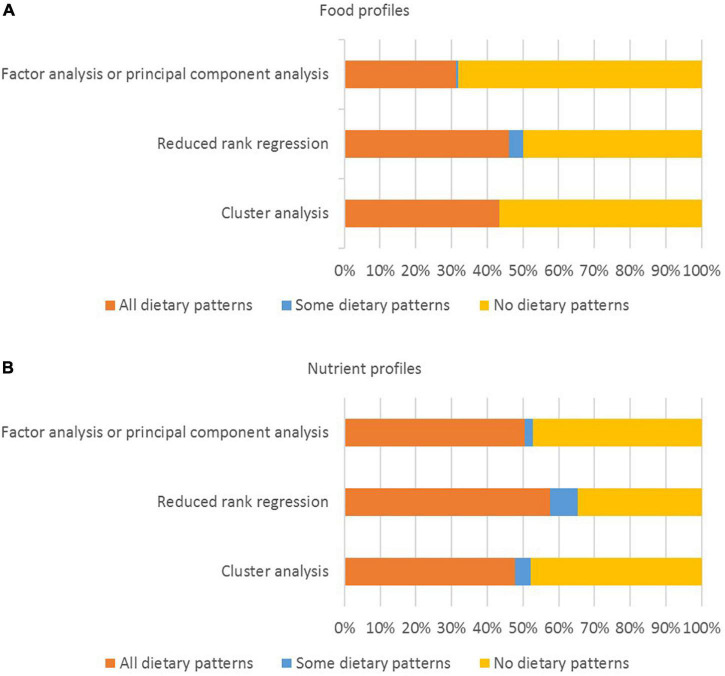
Percentage of studies for which **(A)** food profiles and **(B)** nutrient profiles were reported (*n* = 167 studies).

The number of dietary patterns retained varied according to the data driven method that was used (often one or two patterns for RRR, two or three patterns for FA/PCA, and more than four patterns for CA). In most studies, results were reported for all the dietary patterns that were analyzed with health outcomes. For FA/PCA and CA, the dietary patterns that were analyzed with health outcomes were frequently named using qualitative labels (e.g., healthy, western, Mediterranean, traditional) (75.2% of FA/PCA studies and 82.6% of CA studies) or based on the names of the foods that characterized the dietary pattern (e.g., vegetables, meat) (47.2% of FA/PCA studies and 60.9% of CA studies). For RRR, basic labels were most commonly used (e.g., pattern 1, 2, 3) (34.6% of RRR studies), followed by the names of the intermediate variables (e.g., estrogen food pattern, c-peptide dietary pattern) (26.9% of RRR studies). Food profiles were reported for all the dietary patterns that were analyzed with health outcomes in 31.2% of FA/PCA studies, 46.2% of RRR studies, and 43.5% of CA studies (Figure 4). Nutrient profiles were reported for all the dietary patterns analyzed in 50.4% of FA/PCA studies, 57.7% of RRR studies, and 47.8% of CA studies. In the analysis with health outcomes, results were stratified by sex in 24.8% of FA/PCA studies, 26.9% of RRR studies, and 34.8% of CA studies.

## Discussion

The aims of this systematic review were to analyze the application and reporting dietary pattern assessment methods, and the reporting of the dietary patterns that were analyzed with health outcomes. Index-based methods and FA/PCA were the most frequently used methods (62.7 and 30.5% of studies, respectively). RRR and CA were used much less frequently (6.3 and 5.6% of studies, respectively). There was considerable variation in the application and reporting of dietary pattern assessment methods. Important methodological details were sometimes omitted, including information on the number and nature of dietary components and the cut-off points for scoring (for index-based methods), and details about the foods that were entered into the analysis and how decisions were made about the number of dietary patterns to retain (for data driven methods). The level of detail used to describe the dietary patterns also varied, and food and nutrient profiles were often not reported.

The most frequently used indices were MD indices and adaptations of the HEI (Supplementary Table 2). The original Mediterranean Diet Score (MDS) was published in 1995 (39). Many adaptations have been developed since this time, including the alternate Mediterranean Diet score (aMED) published in 2005 (21, 40). The original HEI was developed in 1995, and revised in 2005 (HEI-2005), 2010 (HEI-2010), and 2015 (HEI-2015) (16, 41). The number of studies that used index-based methods increased dramatically from 2012 (Figure 2). This increase coincides with the availability of the HEI-2010 (42) and the Alternative Healthy Eating Index (AHEI-2010) (43). An important difference between index-based and data driven methods is that index-based methods are more suitable for the purpose of monitoring and surveillance (2, 14, 16). For example, the original HEI and its revisions have been used to monitor adherence to the Dietary Guidelines for Americans (16, 41). Compared to data driven methods, the application of index-based methods may be considered less complex by some researchers in terms of the statistical approaches that are used, and publicly available algorithms or analysis code. These factors may contribute to the more frequent use of index-based methods compared to data driven methods.

FA/PCA and CA have been used as dietary pattern assessment methods since the 1980s (4), compared to RRR which was described by Hoffmann et al. as a “new method” for deriving dietary patterns in 2004 (44). Between 2005 and 2019, FA/PCA continued to be used more frequently than RRR and CA (Figure 2). Each method has advantages and disadvantages, and the most suitable method should be selected based on the research question (2, 6, 14). For example, FA/PCA are used to identify food groups that are highly correlated (i.e., food groups that are often consumed together) (2, 45). In contrast, CA is used to identify mutually exclusive groups of people with similar patterns of dietary intake, but this can impact statistical power (6, 45). RRR is used to understand how particular dietary patterns may contribute to particular health outcomes, but is dependent on selection and availability of appropriate intermediate variables (2, 6, 45). These differences may explain why FA/PCA were used more frequently than CA and RRR.

There was considerable variation in the application and reporting of dietary pattern assessment methods. For example, the application of MD indices varied in terms of the nature of dietary components (64.2% included food and nutrients and 25.1% included foods only), and the rationale behind the cut-off points (61.0% used a combination of absolute and data driven cut-off points, 16.0% used data driven cut-off points only, and 6.4% used absolute cut-off points only) (Supplementary Table 2). Variation in the application of MD indices has also been reported elsewhere (21, 46, 47). Variation in the application and reporting of index-based methods can make it difficult to compare and synthesize results from different studies (7, 14, 15). For example, differences in the components that are included in MD indices and the use of absolute compared to data-driven cut-off points may contribute to differences in effect estimates across studies. This may have implications for evidence use in dietary guideline development because consistent evidence on associations between exposures and outcomes of interest is needed to warrant translation into dietary guidelines (1, 17, 23).

In the application of data driven methods, variation was observed in relation to the number of food groups that were entered into the dietary pattern analysis, the criteria used to determine the food groups, the number of dietary patterns that were retained, and how those dietary patterns were named (Supplementary Table 3). Similar results were reported in a review of 189 studies that that used FA/PCA, RRR or CA published in 2015 (12). Variation in the application of similar data driven methods can influence results. For example, McCann et al. demonstrated that changing the number of food groups that were entered into the dietary pattern analysis had an impact the results of the analysis with the health outcome of interest (48). Further research is needed to understand the impact that particular decisions in relation to the application of dietary pattern assessment methods have on results, and to build consensus on the application and reporting of particular methods.

The level of detail used to describe the dietary patterns that were analyzed with health outcomes varied, and food and nutrient profiles were often not provided (Supplementary Tables 2, 3 and Figures 3, 4). When dietary patterns are derived using indices with absolute cut-off points, some assumptions can be made about the composition of the dietary patterns consumed by people in the highest compared to the lowest quantile. However, for indices that use data-driven cut-off points, these assumptions are difficult to make, because even people in the highest quantile may have a low absolute level of intake of a particular food group in the dietary pattern (depending on the mean or median intake within the study population) (15, 21, 22). Similarly, for dietary patterns derived using FA/PCA, factor loadings provide information on the food groups that are highly correlated, but they don’t provide quantitative information on the foods that were consumed (2, 45). Further research is needed to build consensus on the level of detail required when reporting dietary patterns that are derived using particular methods.

To ensure results of individual studies can be compared and synthesized, a higher level of standardization in the reporting of dietary patterns research is needed (2, 7, 15–17). Reporting guidelines for observational studies in nutritional epidemiology were published in 2016 (49). Strengthening the Reporting of Observational Studies in Epidemiology-Nutritional Epidemiology (STROBE-nut) provides general information on how the application of index-based and data driven dietary pattern assessment methods should be reported, and how results of the analysis with health outcomes should be discussed (49). Kirkpatrick et al. developed a more detailed reporting checklist for the application of the HEI, and suggested that this checklist could be used in conjunction with STROBE-nut (16). Further research is needed to inform the development of detailed reporting guidelines that would make evidence from dietary patterns research easier to synthesize, and could support the translation of this type of evidence into dietary guidelines. More detail could be added to the relevant items in STROBE-nut, or stand-alone guidelines for reporting dietary patterns research could be developed.

### Strengths and Limitations

This systematic review examined a large number of studies (*n* = 410 studies) that were published over a period of 24 years (1995 to 2019). Although the literature search was conducted in 2019, this review focuses on the application and reporting of dietary pattern assessment methods (rather than the associations between dietary patterns and health outcomes) and while reporting may have improved due to the publication of the STROBE-nut guidelines, this paper provides an important snapshot of a large body of literature. This systematic review was limited to cohort and nested case control studies published in English. Analysis of the methods used to assess and report dietary patterns in case control studies, and in studies published in languages other than English would be beneficial. Only studies that used index-based methods, FA/PCA, RRR or CA were eligible for inclusion. However, compared to recently published systematic reviews that analyzed index-based or data driven methods only (11, 21, 27, 28), a strength of this review is that the application and reporting of both index-based and data driven methods were included and analyzed. The analysis of a large number of variables in relation to the application and reporting of index-based and data driven methods, combined with the analysis of how the dietary patterns that were analyzed with health outcomes were reported makes this systematic review a valuable contribution to the literature.

### Conclusion

This systematic review provides a comprehensive examination of how dietary patterns assessment methods are applied and reported, and the level of detail used to report the dietary patterns that are analyzed with health outcomes. The most frequently used methods were index-based methods and FA/PCA, and a small proportion of included studies used RRR and CA. There was considerable variation in the application and reporting of dietary pattern assessment methods, and in some cases, important information was omitted. The level of detail used to describe the dietary patterns also varied, and food and nutrient profiles were often not reported. To ensure evidence from dietary patterns can be synthesized and translated into dietary guidelines, a higher level of standardization in the application and reporting of dietary pattern assessment methods, and the reporting of the dietary patterns that are analyzed with health outcomes would be beneficial. Further research is need to inform the development of reporting guidelines for dietary patterns research.

## Author Contributions

KW performed the original literature search (September 2018), extracted and analyzed the data with support from SM, and prepared the manuscript. SM and ML edited the manuscript. All authors contributed to review conceptualization and methodology, study selection, and read and approved the final version of the manuscript.

## Conflict of Interest

The authors declare that the research was conducted in the absence of any commercial or financial relationships that could be construed as a potential conflict of interest.

## Publisher’s Note

All claims expressed in this article are solely those of the authors and do not necessarily represent those of their affiliated organizations, or those of the publisher, the editors and the reviewers. Any product that may be evaluated in this article, or claim that may be made by its manufacturer, is not guaranteed or endorsed by the publisher.

## References

[1] Dietary Guidelines Advisory Committee [DGAC]. *Scientific Report of the 2020 Dietary Guidelines Advisory Committee: Advisory Report to the Secretary of Agriculture and the Secretary of Health and Human Services.* Washington, DC: United States Department of Agriculture (2020).

[2] McNaughton SA. Dietary patterns. In: Marriott BP Birt DF Stallings VA Yates AA editors. *Present Knowledge in Nutrition.* Washington, DC: International Life Sciences Institute (2020). p. 235–48.

[3] Reedy J Krebs-Smith SM Hammond RA Hennessy E. Advancing the science of dietary patterns research to leverage a complex systems approach. *J Acad Nutr Diet.* (2017) 117:1019–22. 10.1016/j.jand.2017.03.008 28465171

[4] Newby PK Tucker KL. Empirically derived eating patterns using factor or cluster analysis: a review. *Nutr Rev.* (2004) 62:177–203. 10.1111/j.1753-4887.2004.tb00040.x 15212319

[5] Panagiotakos D. A -priori versus α-posterior methods in dietary pattern analysis: a review in nutrition epidemiology. *Nutr Bull.* (2008) 33:311–5. 10.1111/j.1467-3010.2008.00731.x

[6] Zhao J Li Z Gao Q Zhao H Chen S Huang L A review of statistical methods for dietary pattern analysis. *Nutr J.* (2021) 20:37. 10.1186/s12937-021-00692-7 33874970 PMC8056502

[7] Ocké MC. Evaluation of methodologies for assessing the overall diet: dietary quality scores and dietary pattern analysis. *Proc Nutr Soc.* (2013) 72:191–9. 10.1017/S0029665113000013 23360896

[8] Aljuraiban GS Gibson R Oude Griep LM Okuda N Steffen LM Van Horn L Perspective: the application of a priori diet quality scores to cardiovascular disease risk - a critical evaluation of current scoring systems. *Adv Nutr.* (2020) 11:10–24. 10.1093/advances/nmz059 31209464 PMC7442364

[9] Fransen HP Ocke MC. Indices of diet quality. *Curr Opin Clin Nutr Metab Care.* (2008) 11:559–65. 10.1097/MCO.0b013e32830a49db 18685450

[10] Waijers PM Feskens EJ Ocke MC. A Critical review of predefined diet quality scores. *Br J Nutr.* (2007) 97:219–31. 10.1017/S0007114507250421 17298689

[11] Jannasch F Riordan F Andersen LF Schulze MB. Exploratory dietary patterns: a systematic review of methods applied in pan-European studies and of validation studies. *Br J Nutr.* (2018) 120:601–11. 10.1017/S0007114518001800 30064527 PMC6137382

[12] Borges CA Rinaldi AE Conde WL Mainardi GM Behar D Slater B. Dietary patterns: a literature review of the methodological characteristics of the main steps of the multivariate analyzes. *Rev Bras Epidemiol.* (2015) 18:837–57. 10.1590/1980-5497201500040013 26982299

[13] Fransen HP May AM Stricker MD Boer JM Hennig C Rosseel Y A posteriori dietary patterns: how many patterns to retain? *J Nutr.* (2014) 144:1274–82. 10.3945/jn.113.188680 24872222

[14] Krebs-Smith SM Subar AF Reedy J. Examining dietary patterns in relation to chronic disease: matching measures and methods to questions of interest. *Circulation.* (2015) 132:790–3. 10.1161/CIRCULATIONAHA.115.018010 26260734

[15] Reedy J Subar AF George SM Krebs-Smith SM. Extending methods in dietary patterns research. *Nutrients.* (2018) 10:571. 10.3390/nu10050571 29735885 PMC5986451

[16] Kirkpatrick SI Reedy J Krebs-Smith SM Pannucci TE Subar AF Wilson MM Applications of the healthy eating index for surveillance, epidemiology, and intervention research: considerations and caveats. *J Acad Nutr Diet.* (2018) 118:1603–21. 10.1016/j.jand.2018.05.020 30146072 PMC6730554

[17] Liese AD Krebs-Smith SM Subar AF George SM Harmon BE Neuhouser ML The dietary patterns methods project: synthesis of findings across cohorts and relevance to dietary guidance. *J Nutr.* (2015) 145:393–402. 10.3945/jn.114.205336 25733454 PMC4336525

[18] Harmon BE Boushey CJ Shvetsov YB Ettienne R Reedy J Wilkens LR Associations of key diet-quality indexes with mortality in the multiethnic cohort: the dietary patterns methods project. *Am J Clin Nutr.* (2015) 101:587–97. 10.3945/ajcn.114.090688 25733644 PMC4340063

[19] Reedy J Krebs-Smith SM Miller PE Liese AD Kahle LL Park Y Higher diet quality is associated with decreased risk of all-cause, cardiovascular disease, and cancer mortality among older adults. *J Nutr.* (2014) 144:881–9. 10.3945/jn.113.189407 24572039 PMC4018951

[20] George SM Ballard-Barbash R Manson JE Reedy J Shikany JM Subar AF Comparing indices of diet quality with chronic disease mortality risk in postmenopausal women in the women’s health initiative observational study: evidence to inform national dietary guidance. *Am J Epidemiol.* (2014) 180:616–25. 10.1093/aje/kwu173 25035143 PMC4157698

[21] Abdelhamid A Jennings A Hayhoe RPG Awuzudike VE Welch AA. High variability of food and nutrient intake exists across the mediterranean dietary pattern - a systematic review. *Food Sci Nutr.* (2020) 8:4907–18. 10.1002/fsn3.1784 32994952 PMC7500794

[22] Hodge A Bassett J. What can we learn from dietary pattern analysis? *Public Health Nutr.* (2016) 19:191–4. 10.1017/S1368980015003730 26784585 PMC10273249

[23] Tapsell LC Neale EP Satija A Hu FB. Foods, nutrients, and dietary patterns: interconnections and implications for dietary guidelines. *Adv Nutr.* (2016) 7:445–54. 10.3945/an.115.011718 27184272 PMC4863273

[24] Mozaffarian D Forouhi NG. Dietary guidelines and health-is nutrition science up to the task? *BMJ.* (2018) 360:k822. 10.1136/bmj.k822 29549076

[25] Wingrove K Lawrence MA McNaughton SA. Dietary patterns, foods and nutrients: a descriptive analysis of the systematic reviews conducted to inform the Australian dietary guidelines. *Nutr Res Rev.* (2020) 34:117–24. 10.1017/S0954422420000190 32779564

[26] Tapsell LC Neale EP Probst Y. Dietary patterns and cardiovascular disease: insights and challenges for considering food groups and nutrient sources. *Curr Atheroscler Rep.* (2019) 21:9. 10.1007/s11883-019-0770-1 30741361 PMC6373325

[27] Trijsburg L Talsma EF de Vries JHM Kennedy G Kuijsten A Brouwer ID. Diet quality indices for research in low- and middle-income countries: a systematic review. *Nutr Rev.* (2019) 77:515–40. 10.1093/nutrit/nuz017 31127835 PMC6609420

[28] Edefonti V De Vito R Dalmartello M Patel L Salvatori A Ferraroni M. Reproducibility and validity of a posteriori dietary patterns: a systematic review. *Adv Nutr.* (2019) 11:293–326. 10.1093/advances/nmz097 31578550 PMC7442345

[29] McNaughton S Lawrence M Stephens L Wingrove K Leech R Livingstone K *Dietary Patterns and Health Outcomes: A Series of Systematic Reviews and Meta-Analyses.* (2021).

[30] Kumanyika S Afshin A Arimond M Lawrence M McNaughton SA Nishida C. Approaches to defining healthy diets: a background paper for the international expert consultation on sustainable healthy diets. *Food Nutr Bull.* (2020) 41:7S–30S. 10.1177/0379572120973111 33356593

[31] Page MJ McKenzie JE Bossuyt PM Boutron I Hoffmann TC Mulrow CD The prisma 2020 statement: an updated guideline for reporting systematic reviews. *BMJ.* (2021) 372:n71. 10.1136/bmj.n71 33782057 PMC8005924

[32] Lawrence M Wingrove K McNaughton S Lee A Pollard C Stanton R *Report on a Scoping Review into Dietary Patterns and Health and Sustainability Impacts with Particular Focus on the Characteristics: Source; Eating Patterns; and Processing.* (2016).

[33] World Health Organization [WHO]. *Handbook for Guideline Development.* Geneva: World Health Organization (2014).

[34] National Institutes of Health [NIH], National Cancer Institute [NCI]. *Dietary Assessment Primer, Instrument Profiles.* (2015). Available online at: https://dietassessmentprimer.cancer.gov/profiles/ (accessed September 9, 2021).

[35] Veritas Health Innovation [VHI]. *Covidence Systematic Review Software.* Melbourne, Vic: Veritas Health Innovation (2021).

[36] Harris PA Taylor R Minor BL Elliott V Fernandez M O’Neal L The redcap consortium: building an international community of software platform partners. *J Biomed Inform.* (2019) 95:103208. 10.1016/j.jbi.2019.103208 31078660 PMC7254481

[37] Harris PA Taylor R Thielke R Payne J Gonzalez N Conde JG. Research electronic data capture (REDCap)–a metadata-driven methodology and workflow process for providing translational research informatics support. *J Biomed Inform.* (2009) 42:377–81. 10.1016/j.jbi.2008.08.010 18929686 PMC2700030

[38] National Institutes of Health [NIH], National Cancer Institute [NCI]. *Dietary Assessment Primer, Energy Adjustment.* (2015). Available online at: https://dietassessmentprimer.cancer.gov/learn/adjustment.html (accessed September 9, 2021).

[39] Trichopoulou A Kouris-Blazos A Wahlqvist ML Gnardellis C Lagiou P Polychronopoulos E Diet and overall survival in elderly people. *BMJ.* (1995) 311:1457–60. 10.1136/bmj.311.7018.1457 8520331 PMC2543726

[40] Fung TT McCullough ML Newby PK Manson JE Meigs JB Rifai N Diet-quality scores and plasma concentrations of markers of inflammation and endothelial dysfunction. *Am J Clin Nutr.* (2005) 82:163–73. 10.1093/ajcn.82.1.163 16002815

[41] National Cancer Institute [NCI]. *Developing the Healthy Eating Index.* (2020). Available online at: https://epi.grants.cancer.gov/hei/developing.html (accessed October 5, 2021).

[42] Guenther PM Casavale KO Reedy J Kirkpatrick SI Hiza HA Kuczynski KJ Update of the healthy eating index: HEI-2010. *J Acad Nutr Diet.* (2013) 113:569–80. 10.1016/j.jand.2012.12.016 23415502 PMC3810369

[43] Chiuve SE Fung TT Rimm EB Hu FB McCullough ML Wang M Alternative dietary indices both strongly predict risk of chronic disease. *J Nutr.* (2012) 142:1009–18. 10.3945/jn.111.157222 22513989 PMC3738221

[44] Hoffmann K Schulze MB Schienkiewitz A Nöthlings U Boeing H. Application of a new statistical method to derive dietary patterns in nutritional epidemiology. *Am J Epidemiol.* (2004) 159:935–44. 10.1093/aje/kwh134 15128605

[45] Schulze MB Hoffmann K. Methodological approaches to study dietary patterns in relation to risk of coronary heart disease and stroke. *Br J Nutr.* (2006) 95:860–9. 10.1079/BJN20061731 16611375

[46] D’Alessandro A De Pergola G. Mediterranean diet and cardiovascular disease: a critical evaluation of a priori dietary indexes. *Nutrients.* (2015) 7:7863–88. 10.3390/nu7095367 26389950 PMC4586562

[47] Davis C Bryan J Hodgson J Murphy K. Definition of the mediterranean diet: a literature review. *Nutrients.* (2015) 7:9139–53. 10.3390/nu7115459 26556369 PMC4663587

[48] McCann SE Marshall JR Brasure JR Graham S Freudenheim JL. Analysis of patterns of food intake in nutritional epidemiology: food classification in principal components analysis and the subsequent impact on estimates for endometrial cancer. *Public Health Nutr.* (2001) 4:989–97. 10.1079/phn2001168 11784412

[49] Lachat C Hawwash D Ocke MC Berg C Forsum E Hornell A Strengthening the reporting of observational studies in epidemiology-nutritional epidemiology (strobe-nut): an extension of the strobe statement. *PLoS Med.* (2016) 13:e1002036. 10.1371/journal.pmed.1002036 27270749 PMC4896435

