# Supplementary material for: A Systematic Review of the Methods Used to Assess and Report Dietary Patterns
Source: Front Nutr. 2022 May 25;9:892351. doi: 10.3389/fnut.2022.892351 (PMC9175067; doi:10.3389/fnut.2022.892351)
Supplement: Supplementary file 1 [file Data_Sheet_1.pdf]

frontiers

Supplementary Material

Contents

Supplementary Table 1. Overview of included studies (n=410 studies)..2

Supplementary Table 2. Application and reporting of index-based dietary pattern assessment methods, and reporting of the dietary patterns that were analysed with health outcomes (n=463 indices) 34

Supplementary Table 3. Application and reporting of data driven dietary pattern assessment methods, and reporting of the dietary patterns that were analysed with health outcomes (n=167 studies).........39

References 52

Supplementary Table 1. Overview of included studies (n=410 studies)

First author	Year	Index-based methods	Data driven methods: FA/PCA	Data driven methods: RRR	Data driven methods: CA	Data driven methods: other
Abe, S (1)	2019	Yes	No	No	No	No
Abu-Saad, K (2)	2019	Yes	No	No	No	No
Agnoli, C (3)	2013	Yes	No	No	No	No
Agnoli, C (4)	2011	Yes	No	No	No	No
Agnoli, C (5)	2018	Yes	No	No	No	No
Agurs-Collins, T (6)	2009	No	Yes	No	No	No
Aigner, A (7)	2018	Yes	No	No	No	No
Akbaraly, T (8)	2011	Yes	No	No	No	No
Akbaraly, T (9)	2013	Yes	Yes	No	No	No
Akesson, A (10)	2014	Yes	No	No	No	No
Akesson, A (11)	2007	No	Yes	No	No	No
Akinyemiju, T (12)	2016	No	Yes	No	No	No
Alhazmi, A (13)	2014	Yes	No	No	No	No

First author	Year	Index-based methods	Data driven methods: FA/PCA	Data driven methods: RRR	Data driven methods: CA	Data driven methods: other
Alvarez-Alvarez, I (14)	2018	Yes	No	No	No	No
Alvarez-Alvarez, I (15)	2018	Yes	No	No	No	No
Anderson, AL (16)	2011	No	No	No	Yes	No
Appannah, G (17)	2015	No	No	Yes	No	No
Arabshahi, S (18)	2017	No	Yes	No	No	No
Asghari, G (19)	2016	Yes	No	No	No	No
Assmann, K (20)	2014	Yes	No	No	No	No
Auerbach, BJ (21)	2017	Yes	No	No	No	No
Ax, E (22)	2014	Yes	No	No	No	No
Baglietto, L (23)	2011	No	Yes	No	No	No
Baik, I (24)	2013	No	Yes	No	No	No
Balder, HF (25)	2005	No	Yes	No	No	No
Bamia, C (26)	2013	Yes	No	No	No	No

First author	Year	Index-based methods	Data driven methods: FA/PCA	Data driven methods: RRR	Data driven methods: CA	Data driven methods: other
Bamia, C (27)	2007	No	Yes	No	No	No
Bantle, A (28)	2016	Yes	No	No	No	No
Baudry, J (29)	2018	Yes	No	No	No	No
Bauer, F (30)	2013	No	Yes	No	No	No
Behrens, G (31)	2013	Yes	No	No	No	No
Belin, RJ (32)	2011	Yes	No	No	No	No
Bellavia, A (33)	2016	Yes	No	No	No	No
Benetou, V (34)	2018	Yes	No	No	No	No
Benetou, V (35)	2013	Yes	No	No	No	No
Benetou, V (36)	2008	Yes	No	No	No	No
Berentzen, NE (37)	2013	Yes	No	No	No	No
Bertoia, ML (38)	2014	Yes	No	No	No	No
Beunza, JJ (39)	2010	Yes	No	No	No	No
Biesbroek, S (40)	2018	No	Yes	No	No	No

First author	Year	Index-based methods	Data driven methods: FA/PCA	Data driven methods: RRR	Data driven methods: CA	Data driven methods: other
Biesbroek, S (41)	2015	No	Yes	Yes	Yes	Yes
Biesbroek, S (42)	2017	Yes	No	No	No	No
Bogumil, D (43)	2019	Yes	No	No	No	No
Bonaccio, M (44)	2018	Yes	No	No	No	No
Bonaccio, M (45)	2017	Yes	No	No	No	No
Bongard, V (46)	2016	Yes	No	No	No	No
Borges, CA (47)	2012	No	Yes	No	No	No
Bosire, C (48)	2013	Yes	No	No	No	No
Buckland, G (49)	2010	Yes	No	No	No	No
Buckland, G (50)	2011	Yes	No	No	No	No
Buckland, G (51)	2009	Yes	No	No	No	No
Buckland, G (52)	2014	Yes	No	No	No	No
Buckland, G (53)	2013	Yes	No	No	No	No
Buckland, G (54)	2015	Yes	No	No	No	No
Bull, CJ (55)	2016	No	No	No	Yes	No

First author	Year	Index-based methods	Data driven methods: FA/PCA	Data driven methods: RRR	Data driven methods: CA	Data driven methods: other
Butler, LM (56)	2008	No	Yes	No	No	No
Butler, LM (57)	2010	Yes	Yes	No	No	No
Byberg, L (58)	2016	Yes	No	No	No	No
Cade, JE (59)	2011	Yes	No	No	No	No
Cai, H (60)	2007	No	Yes	No	No	No
Canchola, AJ (61)	2015	No	Yes	No	No	No
Catsburg, C (62)	2015	No	Yes	No	No	No
Cespedes, EM (63)	2016	Yes	No	No	No	No
Chan, R (64)	2013	Yes	Yes	No	No	No
Chan, RSM (65)	2019	Yes	Yes	No	No	No
Chang, ET (66)	2008	No	Yes	No	No	No
Chen, Y (67)	2013	No	Yes	No	No	No
Chen, Z (68)	2018	Yes	No	No	No	No
Chiuve, SE (69)	2011	Yes	No	No	No	No

First author	Year	Index-based methods	Data driven methods: FA/PCA	Data driven methods: RRR	Data driven methods: CA	Data driven methods: other
Chiuve, SE (70)	2006	Yes	No	No	No	No
Chiuve, SE (71)	2008	Yes	No	No	No	No
Chomistek, AK (72)	2015	Yes	No	No	No	No
Conklin, AI (73)	2016	Yes	No	No	No	No
Cottet, V (74)	2009	No	Yes	No	No	No
Couto, E (75)	2011	Yes	No	No	No	No
Couto, E (76)	2013	Yes	No	No	No	No
Cutler, GJ (77)	2012	No	Yes	No	No	No
Dahm, CC (78)	2016	Yes	No	No	No	No
Dai, J (79)	2016	Yes	No	No	No	No
Dai, Z (80)	2014	Yes	Yes	No	No	No
Dartois, L (81)	2016	No	Yes	No	No	No
de Jonge, EAL (82)	2018	No	Yes	No	No	No
de Jonge, EAL (83)	2015	Yes	No	No	No	No

**First author**	**Year**	**Index-based methods**	**Data driven methods: FA/PCA**	**Data driven methods: RRR**	**Data driven methods: CA**	**Data driven methods: other**
de Oliveira Otto, MC (84)	2015	Yes	No	No	No	No
Del Gobbo, LC (85)	2015	Yes	No	No	No	No
Deschasaux, M (86)	2018	Yes	No	No	No	No
Díaz-Gutiérrez, J (87)	2018	Yes	No	No	No	No
Diethelm, K (88)	2014	No	Yes	Yes	No	No
Dilis, V (89)	2012	Yes	No	No	No	No
Dixon, LB (90)	2004	No	Yes	No	No	No
Drake, I (91)	2018	No	No	Yes	No	No
Drewnowski, A (92)	2016	Yes	No	No	No	No
Drogan, D (93)	2007	No	No	Yes	No	No
Duffey, KJ (94)	2012	No	No	No	Yes	No
Dugue, PA (95)	2016	Yes	No	No	No	No
Durao, C (96)	2017	No	No	No	Yes	No

First author	Year	Index-based methods	Data driven methods: FA/PCA	Data driven methods: RRR	Data driven methods: CA	Data driven methods: other
Eguaras, S (97)	2017	Yes	No	No	No	No
Eguaras, S (98)	2015	Yes	No	No	No	No
Engeset, D (99)	2009	No	No	No	Yes	No
Erber, E (100)	2010	No	Yes	No	No	No
Ericson, U (101)	2018	No	Yes	No	No	No
Ericson, U (102)	2018	Yes	No	No	No	No
Fagherazzi, G (103)	2018	No	Yes	No	No	No
Farhadnejad, H (104)	2018	Yes	No	No	No	No
Fasanelli, F (105)	2017	Yes	No	No	No	No
Feart, C (106)	2013	Yes	No	No	No	No
Fidanza, F (107)	2004	Yes	No	No	No	No
Fitzgerald, KC (108)	2012	Yes	No	No	No	No
Florencio, TMMT (109)	2015	Yes	No	No	No	No

First author	Year	Index-based methods	Data driven methods: FA/PCA	Data driven methods: RRR	Data driven methods: CA	Data driven methods: other
Folsom, AR (110)	2007	Yes	No	No	No	No
Ford, DW (111)	2014	Yes	No	No	No	No
Forget, G (112)	2013	Yes	No	No	No	No
Franzon, K (113)	2017	Yes	No	No	No	No
Fresán, U (114)	2019	Yes	No	No	No	No
Fresán, U (115)	2019	Yes	Yes	No	No	No
Fung, TT (116)	2008	Yes	No	No	No	No
Fung, TT (117)	2011	Yes	No	No	No	No
Fung, TT (118)	2006	Yes	No	No	No	No
Fung, TT (119)	2012	No	No	Yes	No	No
Fung, TT (120)	2018	Yes	No	No	No	No
Fung, TT (121)	2015	Yes	No	No	No	No
Fung, TT (122)	2016	Yes	No	No	No	No
Fung, TT (123)	2009	Yes	No	No	No	No
Fung, TT (124)	2012	No	No	Yes	No	No

First author	Year	Index-based methods	Data driven methods: FA/PCA	Data driven methods: RRR	Data driven methods: CA	Data driven methods: other
Fung, TT (125)	2004	No	Yes	No	No	No
Fung, TT (126)	2001	No	Yes	No	No	No
Galbete, C (127)	2018	Yes	No	No	No	No
Garcia-Arellano, A (128)	2018	Yes	No	No	No	No
Gardener, H (129)	2011	Yes	No	No	No	No
Garralda-Del-Villar, M (130)	2019	Yes	No	No	No	No
George, SM (131)	2015	Yes	No	No	No	No
George, SM (132)	2014	Yes	No	No	No	No
Gómez-Donoso, C (133)	2018	Yes	No	No	No	No
Granic, A (134)	2013	No	No	No	Yes	No
Guallar-Castillon, P (135)	2012	No	Yes	No	No	No
Guinter, MA (136)	2018	No	No	Yes	No	No

First author	Year	Index-based methods	Data driven methods: FA/PCA	Data driven methods: RRR	Data driven methods: CA	Data driven methods: other
Guinter, MA (137)	2018	Yes	No	No	No	No
Gunge, VB (138)	2017	Yes	No	No	No	No
Hansen, CP (139)	2017	Yes	No	No	No	No
Hansen, CP (140)	2018	Yes	No	No	No	No
Hansen, SH (141)	2018	Yes	No	No	No	No
Haridass, V (142)	2018	Yes	No	No	No	No
Haring, B (143)	2016	Yes	No	No	No	No
Harmon, BE (144)	2015	Yes	No	No	No	No
Harnack, L (145)	2002	Yes	No	No	No	No
Harrington, JM (146)	2014	No	No	No	Yes	No
Harris, HR (147)	2015	No	No	Yes	No	No
Harriss, LR (148)	2007	No	Yes	No	No	No
Hassannejad, R (149)	2018	No	Yes	No	No	No

First author	Year	Index-based methods	Data driven methods: FA/PCA	Data driven methods: RRR	Data driven methods: CA	Data driven methods: other
Heidemann, C (150)	2005	No	No	Yes	No	No
Heidemann, C (151)	2008	No	Yes	No	No	No
Hengeveld, LM (152)	2018	Yes	No	No	No	No
Heroux, M (153)	2010	No	No	Yes	No	No
Hidaka, BH (154)	2017	No	Yes	No	No	No
Hirko, KA (155)	2016	Yes	No	No	No	No
Hlebowicz, J (156)	2013	Yes	No	No	No	No
Hlebowicz, J (157)	2011	No	No	No	Yes	No
Hodge, AM (158)	2018	Yes	No	No	No	No
Hodge, AM (159)	2016	Yes	No	No	No	No
Hodge, AM (160)	2007	No	Yes	No	No	No
Hoevenaar-Blom, MP (161)	2012	Yes	No	No	No	No

First author	Year	Index-based methods	Data driven methods: FA/PCA	Data driven methods: RRR	Data driven methods: CA	Data driven methods: other
Hosseini-Esfahani, F (162)	2014	No	Yes	No	No	No
Hu, FB (163)	2000	No	Yes	No	No	No
Huijbregts, P (164)	1997	Yes	No	No	No	No
Inoue-Choi, M (165)	2011	No	Yes	No	No	No
Jacobs, S (166)	2017	Yes	No	No	No	No
Jacobs, S (167)	2015	Yes	No	No	No	No
Jacobs, S (168)	2016	Yes	No	No	No	No
Jones, NRV (169)	2018	Yes	No	No	No	No
Jones, P (170)	2017	Yes	No	No	No	No
Judd, SE (171)	2013	No	Yes	No	No	No
Julia, C (172)	2015	Yes	No	No	No	No
Kaluza, J (173)	2009	Yes	No	No	No	No
Kaluza, J (174)	2018	Yes	No	No	No	No

First author	Year	Index-based methods	Data driven methods: FA/PCA	Data driven methods: RRR	Data driven methods: CA	Data driven methods: other
Kane-Diallo, A (175)	2018	Yes	No	No	No	No
Kant, AK (176)	2004	Yes	Yes	No	Yes	No
Kant, AK (177)	2000	Yes	No	No	No	No
Kesse, E (178)	2006	No	Yes	No	No	No
Kesse-Guyot, E (179)	2013	Yes	No	No	No	No
Kim, M (180)	2005	No	Yes	No	No	No
Kimokoti, RW (181)	2012	No	No	No	Yes	No
Knoops, KTB (182)	2004	Yes	No	No	No	No
Knoops, KTB (183)	2006	Yes	No	No	No	No
Kojima, R (184)	2017	No	Yes	No	No	No
Koloverou, E (185)	2016	No	Yes	No	No	No
Koloverou, E (186)	2016	Yes	No	No	No	No

**First author**	**Year**	**Index-based methods**	**Data driven methods: FA/PCA**	**Data driven methods: RRR**	**Data driven methods: CA**	**Data driven methods: other**
Kouris-Blazos, A (187)	1999	Yes	No	No	No	No
Kouvari, M (188)	2019	Yes	No	No	No	No
Kroger, J (189)	2014	Yes	No	Yes	No	No
Kromhout, D (190)	2018	Yes	No	No	No	No
Kumagai, Y (191)	2014	No	Yes	No	No	No
Kurotani, K (192)	2016	Yes	No	No	No	No
Kyro, C (193)	2013	Yes	No	No	No	No
Lachman, S (194)	2015	Yes	No	No	No	No
Lacoppidan, SA (195)	2015	Yes	No	No	No	No
Lagiou, P (196)	2006	Yes	No	No	No	No
Langsetmo, L (197)	2011	No	Yes	No	No	No
Langsetmo, L (198)	2010	No	Yes	No	No	No
Larsson, SC (199)	2014	Yes	No	No	No	No

First author	Year	Index-based methods	Data driven methods: FA/PCA	Data driven methods: RRR	Data driven methods: CA	Data driven methods: other
Larsson, SC (200)	2014	Yes	No	No	No	No
Larsson, SC (201)	2016	Yes	No	No	No	No
Larsson, SC (202)	2016	Yes	No	No	No	No
Larsson, SC (203)	2018	Yes	No	No	No	No
Lassale, C (204)	2012	Yes	No	No	No	No
Lassale, C (205)	2016	Yes	No	No	No	No
Lavalette, C (206)	2018	Yes	No	No	No	No
Leary, SD (207)	2015	No	Yes	No	No	No
Lee, MS (208)	2011	Yes	No	No	No	No
Leenders, M (209)	2015	Yes	No	No	No	No
Lemming, EW (210)	2017	No	Yes	No	No	No
Lemming, EW (211)	2018	Yes	No	No	No	No
Letois, F (212)	2016	Yes	No	No	No	No
Levitan, EB (213)	2009	Yes	No	No	No	No

**First author**	**Year**	**Index-based methods**	**Data driven methods: FA/PCA**	**Data driven methods: RRR**	**Data driven methods: CA**	**Data driven methods: other**
Levitan, EB (214)	2009	Yes	No	No	No	No
Ley, SH (215)	2016	Yes	No	No	No	No
Li, M (216)	2017	No	Yes	No	No	No
Li, P (217)	2016	No	Yes	No	No	No
Li, W (218)	2013	Yes	No	No	No	No
Li, WQ (219)	2014	Yes	No	No	No	No
Li, WQ (220)	2014	Yes	No	No	No	No
Li, Y (221)	2015	Yes	No	No	No	No
Li, Y (222)	2015	Yes	No	No	No	No
Li, Y (223)	2018	Yes	No	No	No	No
Liese, AD (224)	2015	Yes	No	No	No	No
Liese, AD (225)	2009	No	No	Yes	No	No
Lim, J (226)	2018	Yes	No	No	No	No
Limongi, F (227)	2017	Yes	No	No	No	No
Link, LB (228)	2013	No	Yes	No	No	No

**First author**	**Year**	**Index-based methods**	**Data driven methods: FA/PCA**	**Data driven methods: RRR**	**Data driven methods: CA**	**Data driven methods: other**
Liu, L (229)	2017	No	No	Yes	No	No
Lopez-Laguna, N (230)	2018	Yes	No	No	No	No
Lutsey, PL (231)	2008	No	Yes	No	No	No
Ma, Y (232)	2019	Yes	No	No	No	No
Makambi, KH (233)	2011	No	Yes	No	No	No
Malekshah, AFT (234)	2016	Yes	No	No	No	No
Malik, VS (235)	2012	No	Yes	No	No	No
Mandalazi, E (236)	2016	Yes	No	No	No	No
Mangano, KM (237)	2017	No	No	No	Yes	No
Mannisto, S (238)	2005	No	Yes	No	No	No
Martinez-Gonzalez, MA (239)	2011	Yes	Yes	No	No	No

First author	Year	Index-based methods	Data driven methods: FA/PCA	Data driven methods: RRR	Data driven methods: CA	Data driven methods: other
Martinez-Gonzalez, MA (240)	2015	No	Yes	No	No	No
Martinez-Gonzalez, MA (241)	2008	Yes	No	No	No	No
Maruyama, K (242)	2013	No	Yes	No	No	No
Masala, G (243)	2007	No	Yes	No	No	No
McCourt, HJ (244)	2014	Yes	No	No	No	No
McCullough, ML (245)	2000	Yes	No	No	No	No
McCullough, ML (246)	2006	Yes	No	No	No	No
McNaughton, SA (247)	2012	Yes	No	No	No	No
Mehta, RS (248)	2017	No	Yes	No	No	No
Mehta, RS (249)	2017	No	Yes	No	No	No
Melaku, YA (250)	2017	No	Yes	No	No	No

First author	Year	Index-based methods	Data driven methods: FA/PCA	Data driven methods: RRR	Data driven methods: CA	Data driven methods: other
Mendez, MA (251)	2006	Yes	No	No	No	No
Menotti, A (252)	1999	No	Yes	No	No	No
Menotti, A (253)	2014	No	Yes	No	No	No
Mertens, E (254)	2017	No	Yes	No	No	No
Meyer, J (255)	2011	No	No	Yes	No	Yes
Michaud, DS (256)	2005	No	Yes	No	No	No
Michels, KB (257)	2002	Yes	No	No	No	No
Millen, BE (258)	2005	No	No	No	Yes	No
Misirli, G (259)	2012	Yes	No	No	No	No
Mohammadifard, N (260)	2017	No	Yes	No	No	No
Molina-Montes, E (261)	2017	Yes	No	No	No	No
Monjardino, T (262)	2014	Yes	No	No	No	No

First author	Year	Index-based methods	Data driven methods: FA/PCA	Data driven methods: RRR	Data driven methods: CA	Data driven methods: other
Monjardino, T (263)	2015	No	No	No	Yes	No
Monma, Y (264)	2010	No	Yes	No	No	No
Montonen, J (265)	2005	No	Yes	No	No	No
Morimoto, A (266)	2012	No	Yes	No	No	No
Moslehi, N (267)	2016	No	Yes	No	No	No
Muller, DC (268)	2009	No	Yes	No	No	No
Nanri, A (269)	2013	No	Yes	No	No	No
Neelakantan, N (270)	2016	Yes	No	No	No	No
Neelakantan, N (271)	2018	Yes	No	No	No	No
Nettleton, JA (272)	2009	No	Yes	No	No	No
Neufcourt, L (273)	2015	Yes	No	No	No	No
Newby, PK (274)	2006	No	Yes	No	No	No

**First author**	**Year**	**Index-based methods**	**Data driven methods: FA/PCA**	**Data driven methods: RRR**	**Data driven methods: CA**	**Data driven methods: other**
Nimptsch, K (275)	2014	No	Yes	No	No	No
Niu, K (276)	2016	No	Yes	No	No	No
Nobbs, HM (277)	2016	No	Yes	No	No	No
Nomura, SJO (278)	2016	Yes	No	No	No	No
Nomura, SJO (279)	2016	Yes	No	No	No	No
Northstone, K (280)	2012	No	Yes	No	No	No
Nothlings, U (281)	2008	No	No	Yes	No	No
Oddy, WH (282)	2018	No	Yes	No	No	No
Odegaard, AO (283)	2014	No	Yes	No	No	No
Ogilvie, RP (284)	2017	No	Yes	No	No	No
Okada, E (285)	2016	No	Yes	No	No	No
Okubo, H (286)	2015	No	Yes	No	No	No

First author	Year	Index-based methods	Data driven methods: FA/PCA	Data driven methods: RRR	Data driven methods: CA	Data driven methods: other
Olsen, A (287)	2011	Yes	No	No	No	No
Orchard, T (288)	2017	Yes	No	No	No	No
Osler, M (289)	2002	Yes	Yes	No	No	No
Osler, M (290)	2001	Yes	Yes	No	No	No
Osler, M (291)	2001	No	Yes	No	No	No
Osler, M (292)	1997	Yes	No	No	No	No
Pachucki, MA (293)	2012	No	No	No	Yes	No
Pala, V (294)	2013	No	Yes	No	No	No
Panagiotakos, DB (295)	2015	Yes	No	No	No	No
Panizza, CE (296)	2018	Yes	No	No	No	No
Park, S (297)	2017	Yes	No	No	No	No
Park, YM (298)	2016	Yes	No	No	No	No
Pastorino, S (299)	2016	No	No	Yes	No	No

First author	Year	Index-based methods	Data driven methods: FA/PCA	Data driven methods: RRR	Data driven methods: CA	Data driven methods: other
Paterson, KE (300)	2018	Yes	No	No	No	No
Petimar, J (301)	2018	Yes	No	No	No	No
Pham, TM (302)	2010	No	Yes	No	No	No
Pilleron, S (303)	2017	No	No	No	Yes	No
Pimenta, AM (304)	2015	Yes	No	No	No	No
Ping-Delfos, WLCS (305)	2015	Yes	No	No	No	No
Prinelli, F (306)	2015	Yes	No	No	No	No
Qi, L (307)	2009	No	Yes	No	No	No
Quatromoni, PA (308)	2002	No	No	No	Yes	No
Rahi, B (309)	2018	Yes	No	No	No	No
Rajaobelina, K (310)	2019	Yes	Yes	No	No	No
Rashidkhani, B (311)	2005	No	Yes	No	No	No

First author	Year	Index-based methods	Data driven methods: FA/PCA	Data driven methods: RRR	Data driven methods: CA	Data driven methods: other
Reedy, J (312)	2010	Yes	Yes	No	Yes	No
Ritchie, LD (313)	2007	No	No	No	Yes	No
Rogers, TS (314)	2018	No	Yes	No	No	No
Romaguera, D (315)	2011	Yes	No	No	No	No
Romaguera, D (316)	2010	Yes	No	No	No	No
Rossi, M (317)	2013	Yes	No	No	No	No
Roswall, N (318)	2014	Yes	No	No	No	No
Roswall, N (319)	2015	Yes	No	No	No	No
Roswall, N (320)	2015	Yes	No	No	No	No
Rumawas, ME (321)	2009	Yes	No	No	No	No
Rutten-Jacobs, LC (322)	2018	Yes	No	No	No	No
Sadeghi, M (323)	2015	Yes	No	No	No	No
Saldanha-Gomes, C (324)	2017	No	Yes	No	No	No

First author	Year	Index-based methods	Data driven methods: FA/PCA	Data driven methods: RRR	Data driven methods: CA	Data driven methods: other
Sanchez-Villegas, A (325)	2006	Yes	No	No	No	No
Satija, A (326)	2016	Yes	No	No	No	No
Schneider, BC (327)	2016	No	No	No	Yes	No
Schulpen, M (328)	2018	Yes	No	No	No	No
Schulpen, M (329)	2019	Yes	No	No	No	No
Schulpen, M (330)	2019	Yes	No	No	No	No
Schulz, M (331)	2008	No	No	Yes	No	No
Schulz, M (332)	2005	No	No	Yes	No	No
Schulze, MB (333)	2005	No	No	Yes	No	No
Seino, F (334)	1997	No	Yes	No	No	No
Seymour, JD (335)	2003	Yes	No	No	No	No
Shah, NS (336)	2018	Yes	No	No	No	No

First author	Year	Index-based methods	Data driven methods: FA/PCA	Data driven methods: RRR	Data driven methods: CA	Data driven methods: other
Shan, Z (337)	2018	Yes	No	No	No	No
Shaw, SC (338)	2018	No	Yes	No	No	No
Sherafat-Kazemzadeh, R (339)	2010	No	No	Yes	No	No
Shi, L (340)	2018	Yes	No	No	No	No
Shimazu, T (341)	2007	No	Yes	No	No	No
Shin, S (342)	2018	No	Yes	No	No	No
Shin, S (343)	2016	No	Yes	No	No	No
Shin, S (344)	2018	No	Yes	No	No	No
Shivappa, N (345)	2017	Yes	No	No	No	No
Shvetsov, YB (346)	2016	Yes	No	No	No	No
Sieri, S (347)	2004	No	Yes	No	No	No
Sijtsma, FP (348)	2015	Yes	No	No	No	No
Sjogren, P (349)	2010	Yes	No	No	No	No

**First author**	**Year**	**Index-based methods**	**Data driven methods: FA/PCA**	**Data driven methods: RRR**	**Data driven methods: CA**	**Data driven methods: other**
Solbak, NM (350)	2017	Yes	No	No	No	No
Sotos-Prieto, M (351)	2015	Yes	No	No	No	No
Steffen, LM (352)	2014	Yes	No	No	No	No
Stefler, D (353)	2017	Yes	No	No	No	No
Stefler, D (354)	2014	Yes	No	No	No	No
Stricker, MD (355)	2013	No	Yes	No	Yes	No
Struijk, EA (356)	2014	Yes	No	No	No	No
Suwaidi, JA (357)	2015	No	Yes	No	No	No
Tabung, FK (358)	2018	No	No	Yes	No	No
Tabung, FK (359)	2018	No	No	Yes	No	No
Tektonidis, TG (360)	2016	Yes	No	No	No	No
Tektonidis, TG (361)	2015	Yes	No	No	No	No
Terry, P (362)	2001	No	Yes	No	No	No

First author	Year	Index-based methods	Data driven methods: FA/PCA	Data driven methods: RRR	Data driven methods: CA	Data driven methods: other
Tharrey, M (363)	2018	No	Yes	No	No	No
Tikk, K (364)	2014	Yes	No	No	No	No
Tobias, DK (365)	2012	Yes	No	No	No	No
Tognon, G (366)	2014	Yes	No	No	No	No
Tognon, G (367)	2014	Yes	No	No	No	No
Tong, TYN (368)	2016	Yes	No	No	No	No
Tortosa, A (369)	2007	Yes	No	No	No	No
Trichopoulou, A (370)	2005	Yes	No	No	No	No
Trichopoulou, A (371)	2010	Yes	No	No	No	No
Trichopoulou, A (372)	2009	Yes	No	No	No	No
Trichopoulou, A (373)	1995	Yes	No	No	No	No
Tseng, M (374)	2004	No	Yes	No	No	No
Tsivgoulis, G (375)	2015	Yes	No	No	No	No

First author	Year	Index-based methods	Data driven methods: FA/PCA	Data driven methods: RRR	Data driven methods: CA	Data driven methods: other
Turati, F (376)	2015	Yes	No	No	No	No
van Dam, RM (377)	2002	No	Yes	No	No	No
Van Dam, RM (378)	2008	Yes	No	No	No	No
van de Laar, RJ (379)	2013	Yes	No	No	No	No
van den Brandt, PA (380)	2011	Yes	No	No	No	No
van den Brandt, PA (381)	2017	Yes	No	No	No	No
Van Den Hooven, EH (382)	2015	No	No	Yes	No	No
Van Horn, L (383)	2012	No	No	No	Yes	No
Vargas, AJ (384)	2016	Yes	No	No	No	No
Veglia, F (385)	2019	Yes	No	No	No	No
Velie, EM (386)	2005	No	Yes	No	No	No
Voortman, T (387)	2017	Yes	No	No	No	No

First author	Year	Index-based methods	Data driven methods: FA/PCA	Data driven methods: RRR	Data driven methods: CA	Data driven methods: other
Wahlqvist, ML (388)	2005	Yes	No	No	No	No
Waijers, PMCM (389)	2006	No	Yes	No	No	No
Walls, HL (390)	2011	Yes	No	No	No	No
Wang, Z (391)	2017	Yes	No	No	No	No
Wang, Z (392)	2018	Yes	No	No	No	No
Ward, KA (393)	2016	No	No	Yes	No	No
Whalen, KA (394)	2017	Yes	No	No	No	No
Wie, GA (395)	2017	No	Yes	No	No	No
Wirfalt, E (396)	2009	No	No	No	Yes	No
Witlox, WJA (397)	2019	Yes	No	No	No	No
Wolters, M (398)	2018	No	Yes	Yes	No	No
Wu, K (399)	2004	No	Yes	No	No	No
Wu, K (400)	2006	No	Yes	No	No	No

**First author**	**Year**	**Index-based methods**	**Data driven methods: FA/PCA**	**Data driven methods: RRR**	**Data driven methods: CA**	**Data driven methods: other**
Xie, J (401)	2014	Yes	No	No	No	No
Yu, D (402)	2015	Yes	No	No	No	No
Yu, D (403)	2018	Yes	No	No	No	No
Yu, D (404)	2014	Yes	No	No	No	No
Yu, R (405)	2011	Yes	Yes	No	No	No
Zamora, D (406)	2010	Yes	No	No	No	No
Zazpe, I (407)	2014	No	Yes	No	No	No
Zhang, W (408)	2013	No	Yes	No	No	No
Zhao, W (409)	2018	No	Yes	No	No	No
Zheng, J (410)	2018	Yes	No	No	No	No

CA: cluster analysis, FA/PCA: factor analysis or principal component analysis, RRR: reduced rank regression.

Supplementary Table 2. Application and reporting of index-based dietary pattern assessment methods, and reporting of the dietary patterns that were analysed with health outcomes (n=463 indices)

Item	n (all indices)	% (all indices)	n (MD indices)	% (MD indices)	n (HEI indices)	% (HEI indices)	n (DASH indices)	% (DASH indices)	n (other indices)	% (other indices)
**Application and reporting of index-based dietary pattern assessment methods**										
**Index modification**										
Yes	152	32.8	67	35.8	23	27.7	5	10.2	57	39.6
No	311	67.2	120	64.2	60	72.3	44	89.8	87	60.4
Total	463	100.0	187	100.0	83	100.0	49	100.0	144	100.0
**Number of dietary components**										
≤10	298	64.4	145	77.5	34	41.0	43	87.8	76	52.8
11 to 20	101	21.8	25	13.4	39	47.0	1	2.0	36	25.0
21-30	16	3.5	0	0.0	0	0.0	0	0.0	16	11.1
30-40	6	1.3	0	0.0	0	0.0	0	0.0	6	4.2

Item	n (all indices)	% (all indices)	n (MD indices)	% (MD indices)	n (HEI indices)	% (HEI indices)	n (DASH indices)	% (DASH indices)	n (other indices)	% (other indices)
>40	3	0.6	0	0.0	0	0.0	0	0.0	3	2.1
Citation provided	38	8.2	16	8.6	10	12.0	5	10.2	7	4.9
Not reported	1	0.2	1	0.5	0	0.0	0	0.0	0	0.0
Total	463	100.0	187	100.0	83	100.0	49	100.0	144	100.0
**Nature of dietary components**										
Foods only	119	25.7	47	25.1	0	0.0	4	8.2	68	47.2
Food and nutrients	285	61.6	120	64.2	61	73.5	39	79.6	65	45.1
Foods and/or nutrients and supplements	13	2.8	0	0.0	9	10.8	0	0.0	4	2.8
Citation provided	45	9.7	19	10.2	13	15.7	6	12.2	7	4.9
Not reported	1	0.2	1	0.5	0	0.0	0	0.0	0	0.0
Total	463	100.0	187	100.0	83	100.0	49	100.00	144	100.0
**Cut-off points for dietary components**										

Item	n (all indices)	% (all indices)	n (MD indices)	% (MD indices)	n (HEI indices)	% (HEI indices)	n (DASH indices)	% (DASH indices)	n (other indices)	% (other indices)
Dichotomous	182	39.3	126	67.4	0	0.0	1	2.0	55	38.2
Proportional	165	35.6	17	9.1	54	65.1	37	75.5	57	39.6
Dichotomous and proportional	31	6.7	14	7.5	8	9.6	0	0.0	9	6.3
Other	0	0.0	0	0.0	0	0.0	0	0.0	0	0.0
Citation provided	83	17.9	28	15.0	21	25.3	11	22.4	23	16.0
Not reported	2	0.4	2	1.1	0	0.0	0	0.0	0	0.0
Total	463	100.0	187	100.0	83	100.0	49	100.0	144	100.0
**Rationale for cut-off points**										
Absolute	130	28.1	12	6.4	41	49.4	2	4.1	75	52.1
Data driven	100	21.6	30	16.0	2	2.4	38	77.6	30	20.8
Absolute and data driven	145	31.3	114	61.0	21	25.3	2	4.1	8	5.6
Other	0	0.0	0	0.0	0	0.0	0	0.0	0	0.0

Item	n (all indices)	% (all indices)	n (MD indices)	% (MD indices)	n (HEI indices)	% (HEI indices)	n (DASH indices)	% (DASH indices)	n (other indices)	% (other indices)
Citation provided	81	17.5	29	15.5	19	22.9	7	14.3	26	18.1
Not reported	7	1.5	2	1.1	0	0.0	0	0.0	5	3.5
Total	463	100.0	187	100.0	83	100.0	49	100.0	144	100.0
**Possible score range**										
Reported	418	90.3	173	92.5	75	90.4	44	89.8	126	87.5
Citation provided	44	9.5	13	7.0	8	9.6	5	10.2	18	12.5
Not reported	1	0.2	1	0.5	0	0.0	0	0.0	0	0.0
Total	463	100.0	187	100.0	83	100.0	49	100.0	144	100.0
**Reporting of the dietary patterns that were analysed with health outcomes**										
**Food profile of dietary pattern**										
Yes	139	30.0	44	23.5	20	24.1	23	46.9	52	36.1
No	324	70.0	143	76.5	63	75.9	26	53.1	92	63.9

Item	n (all indices)	% (all indices)	n (MD indices)	% (MD indices)	n (HEI indices)	% (HEI indices)	n (DASH indices)	% (DASH indices)	n (other indices)	% (other indices)
Total	463	100.0	187	100.0	83	100.0	49	100.0	144	100.0
**Nutrient profile of dietary pattern**										
Yes	228	49.2	81	43.3	46	55.4	29	59.2	72	50.0
No	235	50.8	106	56.7	37	44.6	20	40.8	72	50.0
Total	463	100.0	187	100.0	83	100.0	49	100.0	144	100.0
**Analysis stratified by sex**										
Yes	160	34.6	61	32.6	34	41.0	17	34.7	48	33.3
No	186	40.2	87	46.5	22	26.5	18	36.7	59	41.0
Not applicable	117	25.3	39	20.9	27	32.5	14	28.6	37	25.7
Total	463	100.0	187	100.0	83	100.0	49	100.0	144	100.0
DASH: Dietary Approaches to Stop Hypertension, HEI: Healthy Eating Index, MD: Mediterranean diet.										

Supplementary Table 3. Application and reporting of data driven dietary pattern assessment methods, and reporting of the dietary patterns that were analysed with health outcomes (n=167 studies)

**Item**	**n (FA/PCA studies)**	**% (FA/PCA studies)**	**n (RRR studies)**	**% (RRR studies)**	**n (CA studies)**	**% (CA studies)**
**Application and reporting of data driven dietary pattern assessment methods**						
**Number of food groups**						
≤20	8	6.4	3	11.5	6	26.1
21-30	27	21.6	1	3.8	3	13.0
31-40	45	36.0	14	53.8	6	26.1
>40	33	26.4	8	30.8	7	30.4
Citation provided	11	8.8	0	0.0	1	4.3
Not reported	1	0.8	0	0.0	0	0.0
Total	125	100.0	26	100.0	23	100.0
**Name of food groups**						
Yes	76	60.8	15	57.7	14	60.9

**Item**	**n (FA/PCA studies)**	**% (FA/PCA studies)**	**n (RRR studies)**	**% (RRR studies)**	**n (CA studies)**	**% (CA studies)**
No	12	9.6	1	3.8	2	8.7
Citation provided	37	29.6	10	38.5	7	30.4
Total	125	100.0	26	100.0	23	100.0
**Criteria for food groups***						
To reflect FFQ	52	41.6	4	15.4	2	8.7
Official agencies/dietary recommendations/previous studies	12	9.6	2	7.7	3	13.0
Food composition/type of food	41	32.8	11	42.3	9	39.1
Dietary intake	6	4.8	2	7.7	3	13.0
Culinary use	26	20.8	6	23.1	1	4.3
Citation provided	24	19.2	9	34.6	8	34.8
Not reported	1	0.8	1	3.8	0	0.0
Total	NA	NA	NA	NA	NA	NA

Item	n (FA/PCA studies)	% (FA/PCA studies)	n (RRR studies)	% (RRR studies)	n (CA studies)	% (CA studies)
**Input unit for food groups**						
Grams	15	12.0	6	23.1	2	8.7
Frequency	23	18.4	5	19.2	5	21.7
% energy	9	7.2	2	7.7	8	34.8
Other	0	0.0	0	0.0	0	0.0
Citation provided	39	31.2	10	38.5	5	21.7
Not reported	39	31.2	3	11.5	3	13.0
Total	125	100.0	26	100.0	23	100.0
**Energy adjustment**						
Nutrient density method	11	8.8	6	23.1	NA	NA
Residual method	28	22.4	5	19.2	NA	NA
No energy adjustment	4	3.2	2	7.7	NA	NA

**Item**	**n (FA/PCA studies)**	**% (FA/PCA studies)**	**n (RRR studies)**	**% (RRR studies)**	**n (CA studies)**	**% (CA studies)**
Adjusted for energy in statistical models	62	49.6	12	46.2	NA	NA
Citation provided	13	10.4	1	3.8	NA	NA
Not reported	7	5.6	0	0.0	NA	NA
Total	125	100.0	26	100.0	NA	NA
**Rotation used**						
Varimax or orthogonal rotation	94	75.2	NA	NA	NA	NA
Direct oblimin (oblique or non-orthogonal) rotation	1	0.8	NA	NA	NA	NA
No rotation	0	0.0	NA	NA	NA	NA
Other	0	0.0	NA	NA	NA	NA
Citation provided	22	17.6	NA	NA	NA	NA
Not reported	8	6.4	NA	NA	NA	NA
Total	125	100.0	NA	NA	NA	NA

Item	n (FA/PCA studies)	% (FA/PCA studies)	n (RRR studies)	% (RRR studies)	n (CA studies)	% (CA studies)
**Calculation of dietary pattern score**						
All foods	97	77.6	7	26.9	NA	NA
Food loading above a certain cut-off point	11	8.8	7	26.9	NA	NA
Other	1	0.8	8	30.8	NA	NA
Citation provided	14	11.2	4	15.4	NA	NA
Not reported	2	1.6	0	0.0	NA	NA
Total	125	100.0	26	100.00	NA	NA
**Factor loading cut-off**						
0.20	3	2.4	7	26.9	NA	NA
0.25	1	0.8	0	0.0	NA	NA
0.30	4	3.2	0	0.0	NA	NA
0.35	1	0.8	0	0.0	NA	NA

**Item**	**n (FA/PCA studies)**	**% (FA/PCA studies)**	**n (RRR studies)**	**% (RRR studies)**	**n (CA studies)**	**% (CA studies)**
0.40	2	1.6	0	0.0	NA	NA
Not applicable	99	79.2	15	57.7	NA	NA
Citation provided	14	11.2	4	15.4	NA	NA
Not reported	1	0.8	0	0.0	NA	NA
Total	125	100.0	26	100.0	NA	NA
**Number of intermediate variables**						
1	NA	NA	2	7.7	NA	NA
2	NA	NA	5	19.2	NA	NA
3	NA	NA	11	42.3	NA	NA
4	NA	NA	3	11.5	NA	NA
>4	NA	NA	4	15.4	NA	NA
Citation provided	NA	NA	1	3.8	NA	NA

Item	n (FA/PCA studies)	% (FA/PCA studies)	n (RRR studies)	% (RRR studies)	n (CA studies)	% (CA studies)
Not reported	NA	NA	0	0.0	NA	NA
Total	NA	NA	26	100.0	NA	NA
**Nature of intermediate variables***						
Disease/risk biomarker	NA	NA	17	65.4	NA	NA
Dietary intake biomarker	NA	NA	0	0.0	NA	NA
Dietary intake	NA	NA	9	34.6	NA	NA
Total	NA	NA	NA	NA	NA	NA
**Type of cluster analysis**						
k-means	NA	NA	NA	NA	11	47.8
Wards method	NA	NA	NA	NA	4	17.4
Latent class analysis	NA	NA	NA	NA	3	13.0
SPSS two-step	NA	NA	NA	NA	1	4.3

Item	n (FA/PCA studies)	% (FA/PCA studies)	n (RRR studies)	% (RRR studies)	n (CA studies)	% (CA studies)
Citation provided	NA	NA	NA	NA	4	17.4
Not reported	NA	NA	NA	NA	0	0.0
Total	NA	NA	NA	NA	23	100.0
**Total percentage of variation explained**						
≤20%	32	25.6	2	7.7	NA	NA
21-30%	31	24.8	0	0.0	NA	NA
31-40%	2	1.6	0	0.0	NA	NA
>40%	11	8.8	3	11.5	NA	NA
Citation provided	31	24.8	10	38.5	NA	NA
Not reported	18	14.4	11	42.3	NA	NA
Total	125	100.0	26	100.0	NA	NA
**Rationale for number of dietary patterns retained***						

Item	n (FA/PCA studies)	% (FA/PCA studies)	n (RRR studies)	% (RRR studies)	n (CA studies)	% (CA studies)
Eigenvalue	91	72.8	0	0.0	0	0.0
Scree plot	84	67.2	0	0.0	0	0.0
Interpretability	76	60.8	2	7.7	4	17.4
Variance explained	8	6.4	14	53.8	4	17.4
Model fit statistics	4	3.2	2	7.7	9	39.1
Number of intermediate variables	0	0.0	2	7.7	0	0.0
Cluster sample size	0	0.0	0	0.0	6	26.1
Other	1	0.8	0	0.0	0	0.0
Citation provided	18	14.4	7	26.9	10	43.5
Not reported	4	3.2	0	0.0	0	0.0
Total	NA	NA	NA	NA	NA	NA
**Reliability of dietary patterns**						

**Item**	**n (FA/PCA studies)**	**% (FA/PCA studies)**	**n (RRR studies)**	**% (RRR studies)**	**n (CA studies)**	**% (CA studies)**
Yes	19	15.2	8	30.8	1	4.3
No	51	40.8	7	26.9	8	34.8
Citation provided	55	44.0	11	42.3	14	60.9
Total	125	100.0	26	100.0	23	100.0
**Number of dietary patterns retained**						
1	10	7.2	15	57.7	0	0.0
2	42	33.6	7	26.9	3	13.0
3	35	28.0	2	7.7	2	8.7
4	21	16.8	0	0.0	5	21.7
>4	17	13.6	2	7.7	13	56.5
Total	125	99.2	26	100.0	23	100.0
**Number of dietary patterns analysed with health outcomes**						

Item	n (FA/PCA studies)	% (FA/PCA studies)	n (RRR studies)	% (RRR studies)	n (CA studies)	% (CA studies)
1	14	11.2	16	61.5	0	0.0
2	42	33.6	7	26.9	4	17.4
3	34	27.2	2	7.7	2	8.7
4	18	14.4	0	0.0	4	17.4
>4	17	13.6	1	3.8	13	56.5
Total	125	100.0	26	100.0	23	100.0
**Reporting of the dietary patterns that were analysed with health outcomes**						
**Dietary pattern nomenclature***						
Qualitative labels	94	75.2	5	19.2	19	82.6
Names of the foods	59	47.2	3	11.5	14	60.9
Nutritional composition	10	8.0	5	19.2	5	21.7
Basic labels	6	4.8	9	34.6	2	8.7

Item	n (FA/PCA studies)	% (FA/PCA studies)	n (RRR studies)	% (RRR studies)	n (CA studies)	% (CA studies)
Intermediate variables	0	0.0	7	26.9	0	0.0
Total	NA	NA	NA	NA	NA	NA
**Food profile of dietary patterns**						
All dietary patterns	39	31.2	12	46.2	10	43.5
Some dietary patterns	1	0.8	1	3.8	0	0.0
No dietary patterns	85	68.0	13	50.0	13	56.5
Total	125	100.0	26	100.0	23	100.0
**Nutrient profile of dietary patterns**						
All dietary patterns	63	50.4	15	57.7	11	47.8
Some dietary patterns	3	2.4	2	7.7	1	4.3
No dietary patterns	59	47.2	9	34.6	11	47.8
Total	125	100.0	26	100	23	100.0

Item	n (FA/PCA studies)	% (FA/PCA studies)	n (RRR studies)	% (RRR studies)	n (CA studies)	% (CA studies)
**Analysis stratified by sex**						
Yes	31	24.8	7	26.9	8	34.8
No	46	36.8	12	46.2	9	39.1
Not applicable	48	38.4	7	26.9	6	26.1
Total	125	100.0	26	100.0	23	100.0

CA: cluster analysis, FA/PCA: factor analysis or principal component analysis, NA: not applicable, RRR: reduced rank regression.
*Categories were not mutually exclusive, so frequencies add up to more than 100%.

References

1. Abe S, Zhang S, Tomata Y, Tsuduki T, Sugawara Y, Tsuji I. Japanese Diet and Survival Time: The Ohsaki Cohort 1994 Study. *Clin Nutr* (2019) 39:298-303. doi: 10.1016/j.clnu.2019.02.010.
2. Abu-Saad K, Endevelt R, Goldsmith R, Shimony T, Nitsan L, Shahar DR, et al. Adaptation and Predictive Utility of a Mediterranean Diet Screener Score. *Clin Nutr* (2019). doi: 10.1016/j.clnu.2018.12.034.
3. Agnoli C, Grioni S, Sieri S, Palli D, Masala G, Sacerdote C, et al. Italian Mediterranean Index and Risk of Colorectal Cancer in the Italian Section of the Epic Cohort. *Int J Cancer* (2013) 132(6):1404-11. doi: 10.1002/ijc.27740.
4. Agnoli C, Krogh V, Grioni S, Sieri S, Palli D, Masala G, et al. A Priori-Defined Dietary Patterns Are Associated with Reduced Risk of Stroke in a Large Italian Cohort. *J Nutr* (2011) 141(8):1552-8. doi: 10.3945/jn.111.140061.
5. Agnoli C, Sieri S, Ricceri F, Giraudo MT, Masala G, Assedi M, et al. Adherence to a Mediterranean Diet and Long-Term Changes in Weight and Waist Circumference in the Epic-Italy Cohort. *Nutr Diabetes* (2018) 8(1). doi: 10.1038/s41387-018-0023-3.
6. Agurs-Collins T, Rosenberg L, Makambi K, Palmer JR, Adams-Campbell L. Dietary Patterns and Breast Cancer Risk in Women Participating in the Black Women's Health Study. *Am J Clin Nutr* (2009) 90(3):621-8. doi: 10.3945/ajcn.2009.27666.
7. Aigner A, Becher H, Jacobs S, Wilkens LR, Boushey CJ, Le Marchand L, et al. Low Diet Quality and the Risk of Stroke Mortality: The Multiethnic Cohort Study. *Eur J Clin Nutr* (2018) 72(7):1035-45. doi: 10.1038/s41430-018-0103-4.
8. Akbaraly TN, Ferrie JE, Berr C, Brunner EJ, Head J, Marmot MG, et al. Alternative Healthy Eating Index and Mortality over 18 Y of Follow-Up: Results from the Whitehall Ii Cohort. *Am J Clin Nutr* (2011) 94(1):247-53. doi: 10.3945/ajcn.111.013128.
9. Akbaraly T, Sabia S, Hagger-Johnson G, Tabak AG, Shipley MJ, Jokela M, et al. Does Overall Diet in Midlife Predict Future Aging Phenotypes? A Cohort Study. *Am J Med* (2013) 126(5):411-9.e3. doi: 10.1016/j.amjmed.2012.10.028.
10. Åkesson A, Larsson SC, Discacciati A, Wolk A. Low-Risk Diet and Lifestyle Habits in the Primary Prevention of Myocardial Infarction in Men: A Population-Based Prospective Cohort Study. *Journal of the American College of Cardiology* (2014) 64(13):1299-306. doi: 10.1016/j.jacc.2014.06.1190.
11. Åkesson A, Weismayer C, Newhy PK, Wolk A. Combined Effect of Low-Risk Dietary and Lifestyle Behaviors in Primary Prevention of Myocardial Infarction in Women. *Arch Intern Med* (2007) 167(19):2122-7. doi: 10.1001/archinte.167.19.2122.
12. Akinyemiju T, Moore JX, Pisu M, Lakoski SG, Shikany J, Goodman M, et al. A Prospective Study of Dietary Patterns and Cancer Mortality among Blacks and Whites in the Regards Cohort. *Int J Cancer* (2016) 139(10):2221-31. doi: 10.1002/ijc.30287.
13. Alhazmi A, Stojanovski E, McEvoy M, Brown W, Garg ML. Diet Quality Score Is a Predictor of Type 2 Diabetes Risk in Women: The Australian Longitudinal Study on Women's Health. *Br J Nutr* (2014) 112(6):945-51. doi: 10.1017/s0007114514001688.
14. Alvarez-Alvarez I, de Rojas JP, Fernandez-Montero A, Zazpe I, Ruiz-Canela M, Hidalgo-Santamaría M, et al. Strong Inverse Associations of Mediterranean Diet, Physical Activity and Their Combination with Cardiovascular Disease: The Seguimiento Universidad De Navarra (Sun) Cohort. *Eur J Prev Cardiol* (2018) 25(11):1186-97. doi: 10.1177/2047487318783263.

15. Alvarez-Alvarez I, Zazpe I, Pérez de Rojas J, Bes-Rastrollo M, Ruiz-Canela M, Fernandez-Montero A, et al. Mediterranean Diet, Physical Activity and Their Combined Effect on All-Cause Mortality: The Seguimiento Universidad De Navarra (Sun) Cohort. *Prev Med* (2018) 106:45-52. doi: 10.1016/j.ypmed.2017.09.021.
16. Anderson AL, Harris TB, Tylavsky FA, Perry SE, Houston DK, Hue TF, et al. Dietary Patterns and Survival of Older Adults. *J Am Diet Assoc* (2011) 111(1):84-91. doi: 10.1016/j.jada.2010.10.012.
17. Appannah G, Pot GK, Huang RC, Oddy WH, Beilin LJ, Mori TA, et al. Identification of a Dietary Pattern Associated with Greater Cardiometabolic Risk in Adolescence. *Nutr Metab Cardiovasc Dis* (2015) 25(7):643-50. doi: 10.1016/j.numecd.2015.04.007.
18. Arabshahi S, Ibiebele TI, Hughes MCB, Lahmann PH, Williams GM, van der Pols JC. Dietary Patterns and Weight Change: 15-Year Longitudinal Study in Australian Adults. *Eur J Nutr* (2017) 56(4):1455-65. doi: 10.1007/s00394-016-1191-3.
19. Asghari G, Yuzbashian E, Mirmiran P, Hooshmand F, Najafi R, Azizi F. Dietary Approaches to Stop Hypertension (Dash) Dietary Pattern Is Associated with Reduced Incidence of Metabolic Syndrome in Children and Adolescents. *J Pediatr* (2016) 174:178-84.e1. doi: 10.1016/j.jpeds.2016.03.077.
20. Assmann KE, Lassale C, Galan P, Hercberg S, Kesse-Guyot E. Dietary Quality and 6-Year Anthropometric Changes in a Sample of French Middle-Aged Overweight and Obese Adults. *PLoS ONE* (2014) 9(2). doi: 10.1371/journal.pone.0087083.
21. Auerbach BJ, Katz R, Tucker K, Boyko EJ, Drewnowski A, Bertoni A, et al. Factors Associated with Maintenance of Body Mass Index in the Jackson Heart Study: A Prospective Cohort Study Secondary Analysis. *Prev Med* (2017) 100:95-100. doi: 10.1016/j.ypmed.2017.04.019.
22. Ax E, Garmo H, Grundmark B, Bill-Axelson A, Holmberg L, Becker W, et al. Dietary Patterns and Prostate Cancer Risk: Report from the Population Based Ulsam Cohort Study of Swedish Men. *Nutr Cancer* (2014) 66(1):77-87. doi: 10.1080/01635581.2014.851712.
23. Baglietto L, Krishnan K, Severi G, Hodge A, Brinkman M, English DR, et al. Dietary Patterns and Risk of Breast Cancer. *Br J Cancer* (2011) 104(3):524-31. doi: 10.1038/sj.bjc.6606044.
24. Baik I, Lee M, Jun N-R, Lee J-Y, Shin C. A Healthy Dietary Pattern Consisting of a Variety of Food Choices Is Inversely Associated with the Development of Metabolic Syndrome. *Nutr Res Pract* (2013) 7(3):233-41. doi: 10.4162/nrp.2013.7.3.233.
25. Balder HF, Goldbohm RA, Van Den Brandt PA. Dietary Patterns Associated with Male Lung Cancer Risk in the Netherlands Cohort Study. *Cancer Epidemiol Biomarkers Prev* (2005) 14(2):483-90. doi: 10.1158/1055-9965.EPI-04-0353.
26. Bamia C, Lagiou P, Buckland G, Grioni S, Agnoli C, Taylor AJ, et al. Mediterranean Diet and Colorectal Cancer Risk: Results from a European Cohort. *Eur J Epidemiol* (2013) 28(4):317-28. doi: 10.1007/s10654-013-9795-x.
27. Bamia C, Trichopoulos D, Ferrari P, Overvad K, Bjerregaard L, Tjønneland A, et al. Dietary Patterns and Survival of Older Europeans: The Epic-Elderly Study (European Prospective Investigation into Cancer and Nutrition). *Public Health Nutr* (2007) 10(6):590-8. doi: 10.1017/S1368980007382487.
28. Bantle AE, Chow LS, Steffen LM, Wang Q, Hughes J, Durant NH, et al. Association of Mediterranean Diet and Cardiorespiratory Fitness with the Development of Pre-Diabetes and Diabetes: The Coronary Artery Risk Development in Young Adults (Cardia) Study. *BMJ Open Diabetes Res Care* (2016) 4(1). doi: 10.1136/bmjdrc-2016-000229.
29. Baudry J, Assmann KE, Touvier M, Allès B, Seconda L, Latino-Martel P, et al. Association of Frequency of Organic Food Consumption with Cancer Risk: Findings from the Nutrinet-Santé

Prospective Cohort Study. *JAMA Intern Med* (2018) 178(12):1597-606. doi: 10.1001/jamainternmed.2018.4357.
30. Bauer F, Beulens JW, van der A Dl, Wijmenga C, Grobbee DE, Spijkerman AM, et al. Dietary Patterns and the Risk of Type 2 Diabetes in Overweight and Obese Individuals. *Eur J Nutr* (2013) 52(3):1127-34. doi: 10.1007/s00394-012-0423-4.
31. Behrens G, Fischer B, Kohler S, Park YK, Hollenbeck AR, Leitzmann MF. Healthy Lifestyle Behaviors and Decreased Risk of Mortality in a Large Prospective Study of U.S. Women and Men. *Eur J Epidemiol* (2013) 28(5):361-72. doi: 10.1007/s10654-013-9796-9.
32. Belin RJ, Greenland P, Allison M, Martin L, Shikany JM, Larson J, et al. Diet Quality and the Risk of Cardiovascular Disease: The Women's Health Initiative (Whi). *Am J Clin Nutr* (2011) 94(1):49-57. doi: 10.3945/ajcn.110.011221.
33. Bellavia A, Tektonidis TG, Orsini N, Wolk A, Larsson SC. Quantifying the Benefits of Mediterranean Diet in Terms of Survival. *Eur J Epidemiol* (2016) 31(5):527-30. doi: 10.1007/s10654-016-0127-9.
34. Benetou V, Orfanos P, Feskanich D, Michaëlsson K, Pettersson-Kymmer U, Byberg L, et al. Mediterranean Diet and Hip Fracture Incidence among Older Adults: The Chances Project. *Osteoporos Int* (2018) 29(7):1591-9. doi: 10.1007/s00198-018-4517-6.
35. Benetou V, Orfanos P, Pettersson-Kymmer U, Bergström U, Svensson O, Johansson I, et al. Mediterranean Diet and Incidence of Hip Fractures in a European Cohort. *Osteoporos Int* (2013) 24(5):1587-98. doi: 10.1007/s00198-012-2187-3.
36. Benetou V, Trichopoulou A, Orfanos P, Naska A, Lagiou P, Boffetta P, et al. Conformity to Traditional Mediterranean Diet and Cancer Incidence: The Greek Epic Cohort. *Br J Cancer* (2008) 99(1):191-5. doi: 10.1038/sj.bjc.6604418.
37. Berentzen NE, Beulens JW, Hoevenaar-Blom MP, Kampman E, Bueno-de-Mesquita HB, Romaguera-Bosch D, et al. Adherence to the Who's Healthy Diet Indicator and Overall Cancer Risk in the Epic-Nl Cohort. *PLoS ONE* (2013) 8(8). doi: 10.1371/journal.pone.0070535.
38. Bertoia ML, Triche EW, Michaud DS, Baylin A, Hogan JW, Neuhouser ML, et al. Mediterranean and Dietary Approaches to Stop Hypertension Dietary Patterns and Risk of Sudden Cardiac Death in Postmenopausal Women1-3. *Am J Clin Nutr* (2014) 99(2):344-51. doi: 10.3945/ajcn.112.056135.
39. Beunza JJ, Toledo E, Hu FB, Bes-Rastrollo M, Serrano-Martínez M, Sánchez-Villegas A, et al. Adherence to the Mediterranean Diet, Long-Term Weight Change, and Incident Overweight or Obesity: The Seguimiento Universidad De Navarra (Sun) Cohort. *Am J Clin Nutr* (2010) 92(6):1484-93. doi: 10.3945/ajcn.2010.29764.
40. Biesbroek S, Kneepkens MC, Van Den Berg SW, Fransen HP, Beulens JW, Peeters PHM, et al. Dietary Patterns within Educational Groups and Their Association with Chd and Stroke in the European Prospective Investigation into Cancer and Nutrition-Netherlands Cohort. *Br J Nutr* (2018) 119(8):949-56. doi: 10.1017/S0007114518000569.
41. Biesbroek S, Van Der ADL, Brosens MCC, Beulens JWJ, Verschuren WMM, Van Der Schouw YT, et al. Identifying Cardiovascular Risk Factor-Related Dietary Patterns with Reduced Rank Regression and Random Forest in the Epic-Nl Cohort. *Am J Clin Nutr* (2015) 102(1):146-54. doi: 10.3945/ajcn.114.092288.
42. Biesbroek S, Verschuren WMM, Boer JMA, Van De Kamp ME, Van Der Schouw YT, Geelen A, et al. Does a Better Adherence to Dietary Guidelines Reduce Mortality Risk and Environmental Impact in the Dutch Sub-Cohort of the European Prospective Investigation into Cancer and Nutrition? *Br J Nutr* (2017) 118(1):69-80. doi: 10.1017/S0007114517001878.

43. Bogumil D, Park SY, Le Marchand L, Haiman CA, Wilkens LR, Boushey CJ, et al. High-Quality Diets Are Associated with Reduced Risk of Hepatocellular Carcinoma and Chronic Liver Disease: The Multiethnic Cohort. *Hepatol Commun* (2019) 3(3):437-47. doi: 10.1002/hep4.1313.
44. Bonaccio M, Di Castelnuovo A, Costanzo S, Gialluisi A, Persichillo M, Cerletti C, et al. Mediterranean Diet and Mortality in the Elderly: A Prospective Cohort Study and a Meta-Analysis. *Br J Nutr* (2018). doi: 10.1017/S0007114518002179.
45. Bonaccio M, Di Castelnuovo A, Pounis G, Costanzo S, Persichillo M, Cerletti C, et al. High Adherence to the Mediterranean Diet Is Associated with Cardiovascular Protection in Higher but Not in Lower Socioeconomic Groups: Prospective Findings from the Moli-Sani Study. *Int J Epidemiol* (2017) 46(5):1478-87. doi: 10.1093/IJE/DYX145.
46. Bongard V, Arveiler D, Dallongeville J, Ruidavets JB, Wagner A, Simon C, et al. Food Groups Associated with a Reduced Risk of 15-Year All-Cause Death. *Eur J Clin Nutr* (2016) 70(6):715-22. doi: 10.1038/ejcn.2016.19.
47. Borges CA, Enes CC, Slater B, Conde WL. Bmi Changes Associated with Dietary Trends among Brazilian Adolescents. *Infant Child Adolesc Nutr* (2012) 4(6):361-8. doi: 10.1177/1941406412451995.
48. Bosire C, Stampfer MJ, Subar AF, Park YK, Kirkpatrick SI, Chiuve SE, et al. Index-Based Dietary Patterns and the Risk of Prostate Cancer in the Nih-Aarp Diet and Health Study. *Am J Epidemiol* (2013) 177(6):504-13. doi: 10.1093/aje/kws261.
49. Buckland G, Agudo A, Luján L, Jakszyn P, Bueno-de-Mesquita HB, Palli D, et al. Adherence to a Mediterranean Diet and Risk of Gastric Adenocarcinoma within the European Prospective Investigation into Cancer and Nutrition (Epic) Cohort Study. *Am J Clin Nutr* (2010) 91(2):381-90. doi: 10.3945/ajcn.2009.28209.
50. Buckland G, Agudo A, Travier N, María Huerta J, Cirera L, Tormo MJ, et al. Adherence to the Mediterranean Diet Reduces Mortality in the Spanish Cohort of the European Prospective Investigation into Cancer and Nutrition (Epic-Spain). *Br J Nutr* (2011) 106(10):1581-91. doi: 10.1017/S0007114511002078.
51. Buckland G, González CA, Agudo A, Vilardell M, Berenguer A, Amiano P, et al. Adherence to the Mediterranean Diet and Risk of Coronary Heart Disease in the Spanish Epic Cohort Study. *Am J Epidemiol* (2009) 170(12):1518-29. doi: 10.1093/aje/kwp282.
52. Buckland G, Ros MM, Roswall N, Bueno-De-Mesquita HB, Travier N, Tjonneland A, et al. Adherence to the Mediterranean Diet and Risk of Bladder Cancer in the Epic Cohort Study. *Int J Cancer* (2014) 134(10):2504-11. doi: 10.1002/ijc.28573.
53. Buckland G, Travier N, Cottet V, González CA, Luján-Barroso L, Agudo A, et al. Adherence to the Mediterranean Diet and Risk of Breast Cancer in the European Prospective Investigation into Cancer and Nutrition Cohort Study. *Int J Cancer* (2013) 132(12):2918-27. doi: 10.1002/ijc.27958.
54. Buckland G, Travier N, Huerta JM, Bueno-De-Mesquita HB, Siersema PD, Skeie G, et al. Healthy Lifestyle Index and Risk of Gastric Adenocarcinoma in the Epic Cohort Study. *Int J Cancer* (2015) 137(3):598-606. doi: 10.1002/ijc.29411.
55. Bull CJ, Northstone K. Childhood Dietary Patterns and Cardiovascular Risk Factors in Adolescence: Results from the Avon Longitudinal Study of Parents and Children (Alspac) Cohort. *Public Health Nutr* (2016) 19(18):3369-77. doi: 10.1017/S1368980016001592.
56. Butler LM, Wang R, Koh WP, Yu MC. Prospective Study of Dietary Patterns and Colorectal Cancer among Singapore Chinese. *Br J Cancer* (2008) 99(9):1511-6. doi: 10.1038/sj.bjc.6604678.
57. Butler LM, Wu AH, Wang RW, Koh W, Yuan JM, Yu MC. A Vegetable-Fruit-Soy Dietary Pattern Protects against Breast Cancer among Postmenopausal Singapore Chinese Women. *Am J Clin Nutr* (2010) 91(4):1013-9. doi: 10.3945/ajcn.2009.28572.

58. Byberg L, Bellavia A, Larsson SC, Orsini N, Wolk A, Michaëlsson K. Mediterranean Diet and Hip Fracture in Swedish Men and Women. *J Bone Miner Res* (2016) 31(12):2098-105. doi: 10.1002/jbmr.2896.
59. Cade JE, Taylor EF, Burley VJ, Greenwood DC. Does the Mediterranean Dietary Pattern or the Healthy Diet Index Influence the Risk of Breast Cancer in a Large British Cohort of Women. *Eur J Clin Nutr* (2011) 65(8):920-8. doi: 10.1038/ejcn.2011.69.
60. Cai H, Shu XO, Gao YT, Li H, Yang G, Zheng W. A Prospective Study of Dietary Patterns and Mortality in Chinese Women. *Epidemiology* (2007) 18(3):393-401. doi: 10.1097/01.ede.0000259967.21114.45.
61. Canchola AJ, Lacey JV, Bernstein L, Horn-Ross PL. Dietary Patterns and Endometrial Cancer Risk in the California Teachers Study Cohort. *Cancer Causes Control* (2015) 26(4):627-34. doi: 10.1007/s10552-015-0552-1.
62. Catsburg C, Kim RS, Kirsh VA, Soskolne CL, Kreiger N, Rohan TE. Dietary Patterns and Breast Cancer Risk: A Study in 2 Cohorts. *Am J Clin Nutr* (2015) 101(4):817-23. doi: 10.3945/ajcn.114.097659.
63. Cespedes EM, Hu FB, Tinker L, Rosner B, Redline S, Garcia L, et al. Multiple Healthful Dietary Patterns and Type 2 Diabetes in the Women's Health Initiative. *Am J Epidemiol* (2016) 183(7):622-33. doi: 10.1093/aje/kwv241.
64. Chan R, Chan D, Woo J. The Association of a Priori and a Posterior Dietary Patterns with the Risk of Incident Stroke in Chinese Older People in Hong Kong. *J Nutr Health Aging* (2013) 17(10):1-9. doi: 10.1007/s12603-013-0334-y.
65. Chan RSM, Yu BWM, Leung J, Lee JSW, Auyeung TW, Kwok T, et al. How Dietary Patterns Are Related to Inflammaging and Mortality in Community-Dwelling Older Chinese Adults in Hong Kong — a Prospective Analysis. *J Nutr Health Aging* (2019) 23(2):181-94. doi: 10.1007/s12603-018-1143-0.
66. Chang ET, Lee VS, Canchola AJ, Dalvi TB, Clarke CA, Reynolds P, et al. Dietary Patterns and Risk of Ovarian Cancer in the California Teachers Study Cohort. *Nutr Cancer* (2008) 60(3):285-91. doi: 10.1080/01635580701733091.
67. Chen Y, McClintock TR, Segers S, Parvez F, Tariqul I, Alauddin A, et al. Prospective Investigation of Major Dietary Patterns and Risk of Cardiovascular Mortality in Bangladesh. *Int J Cardiol* (2013) 167(4):1495-501. doi: 10.1016/j.ijcard.2012.04.041.
68. Chen Z, Zuurmond MG, van der Schaft N, Nano J, Wijnhoven HAH, Ikram MA, et al. Plant Versus Animal Based Diets and Insulin Resistance, Prediabetes and Type 2 Diabetes: The Rotterdam Study. *Eur J Epidemiol* (2018) 33(9):883-93. doi: 10.1007/s10654-018-0414-8.
69. Chiuve SE, Fung TT, Rexrode KM, Spiegelman D, Manson JE, Stampfer MJ, et al. Adherence to a Low-Risk, Healthy Lifestyle and Risk of Sudden Cardiac Death among Women. *JAMA* (2011) 306(1):62-9. doi: 10.1001/jama.2011.907.
70. Chiuve SE, McCullough ML, Sacks FM, Rimm EB. Healthy Lifestyle Factors in the Primary Prevention of Coronary Heart Disease among Men: Benefits among Users and Nonusers of Lipid-Lowering and Antihypertensive Medications. *Circulation* (2006) 114(2):160-7. doi: 10.1161/CIRCULATIONAHA.106.621417.
71. Chiuve SE, Rexrode KM, Spiegelman D, Logroscino G, Manson JE, Rimm EB. Primary Prevention of Stroke by Healthy Lifestyle. *Circulation* (2008) 118(9):947-54. doi: 10.1161/CIRCULATIONAHA.108.781062.
72. Chomistek AK, Chiuve SE, Eliassen AH, Mukamal KJ, Willett WC, Rimm EB. Healthy Lifestyle in the Primordial Prevention of Cardiovascular Disease among Young Women. *Journal of the American College of Cardiology* (2015) 65(1):43-51. doi: 10.1016/j.jacc.2014.10.024.

73. Conklin AI, Monsivais P, Khaw KT, Wareham NJ, Forouhi NG. Dietary Diversity, Diet Cost, and Incidence of Type 2 Diabetes in the United Kingdom: A Prospective Cohort Study. *PLoS Med* (2016) 13(7). doi: 10.1371/journal.pmed.1002085.
74. Cottet V, Touvier M, Fournier A, Touillaud MS, Lafay L, Clavel-Chapelon F, et al. Postmenopausal Breast Cancer Risk and Dietary Patterns in the E3n-Epic Prospective Cohort Study. *Am J Epidemiol* (2009) 170(10):1257-67. doi: 10.1093/aje/kwp257.
75. Couto E, Boffetta P, Lagiou P, Ferrari P, Buckland G, Overvad K, et al. Mediterranean Dietary Pattern and Cancer Risk in the Epic Cohort. *Br J Cancer* (2011) 104(9):1493-9. doi: 10.1038/bjc.2011.106.
76. Couto E, Sandin S, Löf M, Ursin G, Adami HO, Weiderpass E. Mediterranean Dietary Pattern and Risk of Breast Cancer. *PLoS ONE* (2013) 8(2). doi: 10.1371/journal.pone.0055374.
77. Cutler GJ, Flood A, Hannan PJ, Slavin JL, Neumark-Sztainer D. Association between Major Patterns of Dietary Intake and Weight Status in Adolescents. *Br J Nutr* (2012) 108(2):349-56. doi: 10.1017/S0007114511005435.
78. Dahm CC, Chomistek AK, Jakobsen MU, Mukamal KJ, Eliassen AH, Sesso HD, et al. Adolescent Diet Quality and Cardiovascular Disease Risk Factors and Incident Cardiovascular Disease in Middle-Aged Women. *J Am Heart Assoc* (2016) 5(12). doi: 10.1161/JAHA.116.003583.
79. Dai J, Krasnow RE, Reed T. Midlife Moderation-Quantified Healthy Diet and 40-Year Mortality Risk from Chd: The Prospective National Heart, Lung, and Blood Institute Twin Study. *Br J Nutr* (2016) 116(2):326-34. doi: 10.1017/S0007114516001914.
80. Dai Z, Butler LM, van Dam RM, Ang L-W, Yuan J-M, Koh W-P. Adherence to a Vegetable-Fruit-Soy Dietary Pattern or the Alternative Healthy Eating Index Is Associated with Lower Hip Fracture Risk among Singapore Chinese. *J Nutr* (2014) 144(4):511-8. doi: 10.3945/jn.113.187955.
81. Dartois L, Fagherazzi G, Baglietto L, Boutron-Ruault MC, Delaloge S, Mesrine S, et al. Proportion of Premenopausal and Postmenopausal Breast Cancers Attributable to Known Risk Factors: Estimates from the E3n-Epic Cohort. *Int J Cancer* (2016) 138(10):2415-27. doi: 10.1002/ijc.29987.
82. de Jonge EAL, Rivadeneira F, Erler NS, Hofman A, Uitterlinden AG, Franco OH, et al. Dietary Patterns in an Elderly Population and Their Relation with Bone Mineral Density: The Rotterdam Study. *Eur J Nutr* (2018) 57(1):61-73. doi: 10.1007/s00394-016-1297-7.
83. de Jonge EAL, Kiefte-de Jong JC, de Groot LCPGM, Voortman T, Schoufour JD, Zillikens MC, et al. Development of a Food Group-Based Diet Score and Its Association with Bone Mineral Density in the Elderly: The Rotterdam Study. *Nutrients* (2015) 7(8):6974-90. doi: 10.3390/nu7085317.
84. De Oliveira Otto MC, Padhye NS, Bertoni AG, Jacobs DR, Mozaffarian D. Everything in Moderation - Dietary Diversity and Quality, Central Obesity and Risk of Diabetes. *PLoS ONE* (2015) 10(10). doi: 10.1371/journal.pone.0141341.
85. Del Gobbo LC, Kalantarian S, Imamura F, Lemaitre R, Siscovick DS, Psaty BM, et al. Contribution of Major Lifestyle Risk Factors for Incident Heart Failure in Older Adults. The Cardiovascular Health Study. *JACC Heart Fail* (2015) 3(7):520-8. doi: 10.1016/j.jchf.2015.02.009.
86. Deschasaux M, Huybrechts I, Murphy N, Julia C, Hercberg S, Srour B, et al. Nutritional Quality of Food as Represented by the Fsam-Nps Nutrient Profiling System Underlying the Nutri-Score Label and Cancer Risk in Europe: Results from the Epic Prospective Cohort Study. *PLoS Med* (2018) 15(9):e1002651-e. doi: 10.1371/journal.pmed.1002651.
87. Díaz-Gutiérrez J, Ruiz-Canela M, Gea A, Fernández-Montero A, Martínez-González MÁ. Association between a Healthy Lifestyle Score and the Risk of Cardiovascular Disease in the Sun Cohort. *Rev Esp Cardiol* (2018) 71(12):1001-9. doi: 10.1016/j.recesp.2017.09.026.

88. Diethelm K, Günther ALB, Schulze MB, Standl M, Heinrich J, Buyken AE. Prospective Relevance of Dietary Patterns at the Beginning and During the Course of Primary School to the Development of Body Composition. *Br J Nutr* (2014) 111(8):1488-98. doi: 10.1017/S0007114513004017.
89. Dilis V, Katsoulis M, Lagiou P, Trichopoulos D, Naska A, Trichopoulou A. Mediterranean Diet and Chd: The Greek European Prospective Investigation into Cancer and Nutrition Cohort. *Br J Nutr* (2012) 108(4):699-709. doi: 10.1017/S0007114512001821.
90. Dixon LB, Balder HF, Virtanen MJ, Rashidkhani B, Männistö S, Krogh V, et al. Dietary Patterns Associated with Colon and Rectal Cancer: Results from the Dietary Patterns and Cancer (Dietscan) Project. *Am J Clin Nutr* (2004) 80(4):1003-11. doi: 10.1093/ajcn/80.4.1003.
91. Drake I, Sonestedt E, Ericson U, Wallström P, Orho-Melander M. A Western Dietary Pattern Is Prospectively Associated with Cardio-Metabolic Traits and Incidence of the Metabolic Syndrome. *Br J Nutr* (2018) 119(10):1168-76. doi: 10.1017/S000711451800079X.
92. Drewnowski A, Aggarwal A, Tang W, Hurvitz PM, Scully J, Stewart O, et al. Obesity, Diet Quality, Physical Activity, and the Built Environment: The Need for Behavioral Pathways. *BMC Public Health* (2016) 16(1153). doi: 10.1186/s12889-016-3798-y.
93. Drogan D, Hoffmann K, Schulz M, Bergmann MM, Boeing H, Weikert C. A Food Pattern Predicting Prospective Weight Change Is Associated with Risk of Fatal but Not with Nonfatal Cardiovascular Disease. *J Nutr* (2007) 137(8):1961-7. doi: 10.1093/jn/137.8.1961.
94. Duffey KJ, Steffen LM, Horn Lv, Jacobs DR, Jr., Popkin BM. Dietary Patterns Matter: Diet Beverages and Cardiometabolic Risks in the Longitudinal Coronary Artery Risk Development in Young Adults (Cardia) Study. *Am J Clin Nutr* (2012) 95(4):909-15. doi: 10.3945/ajcn.111.026682.
95. Dugué PA, Hodge AM, Brinkman MT, Bassett JK, Shivappa N, Hebert JR, et al. Association between Selected Dietary Scores and the Risk of Urothelial Cell Carcinoma: A Prospective Cohort Study. *Int J Cancer* (2016) 139(6):1251-60. doi: 10.1002/ijc.30175.
96. Durão C, Severo M, Oliveira A, Moreira P, Guerra A, Barros H, et al. Association between Dietary Patterns and Adiposity from 4 to 7 Years of Age. *Public Health Nutr* (2017) 20(11):1973-82. doi: 10.1017/S1368980017000854.
97. Eguaras S, Bes-Rastrollo M, Ruiz-Canela M, Carlos S, Rosa Pdl, Martínez-González MA. May the Mediterranean Diet Attenuate the Risk of Type 2 Diabetes Associated with Obesity: The Seguimiento Universidad De Navarra (Sun) Cohort. *Br J Nutr* (2017) 117(10):1478-85. doi: 10.1017/S0007114517001404.
98. Eguaras S, Toledo E, Hernández-Hernández A, Cervantes S, Martínez-González MA. Better Adherence to the Mediterranean Diet Could Mitigate the Adverse Consequences of Obesity on Cardiovascular Disease: The Sun Prospective Cohort. *Nutrients* (2015) 7(11):9154-62. doi: 10.3390/nu7115457.
99. Engeset D, Dyachenko A, Ciampi A, Lund E. Dietary Patterns and Risk of Cancer of Various Sites in the Norwegian European Prospective Investigation into Cancer and Nutrition Cohort: The Norwegian Women and Cancer Study. *Eur J Cancer Prev* (2009) 18(1):69-75. doi: 10.1097/CEJ.0b013e328305a091.
100. Erber E, Hopping BN, Grandinetti A, Park SY, Kolonel LN, Maskarinec G. Dietary Patterns and Risk for Diabetes: The Multiethnic Cohort. *Diabetes Care* (2010) 33(3):532-8. doi: 10.2337/dc09-1621.
101. Ericson U, Brunkwall L, Alves Dias J, Drake I, Hellstrand S, Gullberg B, et al. Food Patterns in Relation to Weight Change and Incidence of Type 2 Diabetes, Coronary Events and Stroke in the Malmö Diet and Cancer Cohort. *Eur J Nutr* (2018):1-14. doi: 10.1007/s00394-018-1727-9.

102. Ericson U, Hindy G, Drake I, Schulz CA, Brunkwall L, Hellstrand S, et al. Dietary and Genetic Risk Scores and Incidence of Type 2 Diabetes. *Genes Nutr* (2018) 13(13):(16 May 2018)-(16 May). doi: 10.1017/S0007114517001404.
103. Fagherazzi G, Gusto G, Mancini FR, Dow C, Rajaobelina K, Balkau B, et al. Determinants of 20-Year Non-Progression to Type 2 Diabetes in Women at Very High Risk: The E3n Cohort Study. *Diabet Med* (2018). doi: 10.1111/dme.13774.
104. Farhadnejad H, Asghari G, Mirmiran P, Azizi F. Dietary Approach to Stop Hypertension Diet and Cardiovascular Risk Factors among 10- to 18-Year-Old Individuals. *Pediatric Obes* (2018) 13(4):185-94. doi: 10.1111/ijpo.12268.
105. Fasanelli F, Zugna D, Giraudo MT, Krogh V, Grioni S, Panico S, et al. Abdominal Adiposity Is Not a Mediator of the Protective Effect of Mediterranean Diet on Colorectal Cancer. *Int J Cancer* (2017) 140(10):2265-71. doi: 10.1002/ijc.30653.
106. Feart C, Lorrain S, Ginder Coupez V, Samieri C, Letenneur L, Paineau D, et al. Adherence to a Mediterranean Diet and Risk of Fractures in French Older Persons. *Osteoporos Int* (2013) 24(12):3031-41. doi: 10.1007/s00198-013-2421-7.
107. Fidanza F, Alberti A, Lanti M, Menotti A. Mediterranean Adequacy Index: Correlation with 25-Year Mortality from Coronary Heart Disease in the Seven Countries Study. *Nutr Metab Cardiovasc Dis* (2004) 14(5):254-8. doi: 10.1016/s0939-4753(04)80052-8.
108. Fitzgerald KC, Chiuve SE, Buring JE, Ridker PM, Glynn RJ. Comparison of Associations of Adherence to a Dietary Approaches to Stop Hypertension (Dash)-Style Diet with Risks of Cardiovascular Disease and Venous Thromboembolism. *J Thromb Haemost* (2012) 10(2):189-98. doi: 10.1111/j.1538-7836.2011.04588.x.
109. Florêncio TMMT, Bueno NB, Clemente APG, Albuquerque FCA, Britto RPA, Ferriolli E, et al. Weight Gain and Reduced Energy Expenditure in Low-Income Brazilian Women Living in Slums: A 4-Year Follow-up Study. *Br J Nutr* (2015) 114(3):462-71. doi: 10.1017/S0007114515001816.
110. Folsom AR, Parker ED, Harnack LJ. Degree of Concordance with Dash Diet Guidelines and Incidence of Hypertension and Fatal Cardiovascular Disease. *Am J Hypertens* (2007) 20(3):225-32. doi: 10.1016/j.amjhyper.2006.09.003.
111. Ford DW, Hartman TJ, Still C, Wood C, Mitchell DC, Erickson P, et al. Body Mass Index, Poor Diet Quality, and Health-Related Quality of Life Are Associated with Mortality in Rural Older Adults. *J Nutr Gerontol Geriatr* (2014) 33(1):23-34. doi: 10.1080/21551197.2014.875819.
112. Forget G, Doyon M, Lacerte G, Labonté M, Brown C, Carpentier AC, et al. Adoption of American Heart Association 2020 Ideal Healthy Diet Recommendations Prevents Weight Gain in Young Adults. *J Acad Nutr Diet* (2013) 113(11):1517-22. doi: 10.1016/j.jand.2013.06.346.
113. Franzon K, Byberg L, Sjögren P, Zethelius B, Cederholm T, Kilander L. Predictors of Independent Aging and Survival: A 16-Year Follow-up Report in Octogenarian Men. *J Am Geriatr Soc* (2017) 65(9):1953-60. doi: 10.1111/jgs.14971.
114. Fresán U, Sabaté J, Martínez-Gonzalez MA, Segovia-Siapco G, de la Fuente-Arrillaga C, Bes-Rastrollo M. Adherence to the 2015 Dietary Guidelines for Americans and Mortality Risk in a Mediterranean Cohort: The Sun Project. *Prev Med* (2019) 118:317-24. doi: 10.1016/j.ypmed.2018.11.015.
115. Fresán U, Martínez-González MA, Sabaté J, Bes-Rastrollo M. Global Sustainability (Health, Environment and Monetary Costs) of Three Dietary Patterns: Results from a Spanish Cohort (the Sun Project). *BMJ Open* (2019) 9(2). doi: 10.1136/bmjopen-2018-021541.

116. Fung TT, Chiuve SE, McCullough ML, Rexrode KM, Logroscino G, Hu FB. Adherence to a Dash-Style Diet and Risk of Coronary Heart Disease and Stroke in Women. *Arch Intern Med* (2008) 168(7):713-20. doi: 10.1001/archinte.168.7.713.
117. Fung TT, Hu FB, Hankinson SE, Willett WC, Holmes MD. Low-Carbohydrate Diets, Dietary Approaches to Stop Hypertension-Style Diets, and the Risk of Postmenopausal Breast Cancer. *Am J Epidemiol* (2011) 174(6):652-60. doi: 10.1093/aje/kwr148.
118. Fung TT, Hu FB, McCullough ML, Newby PK, Willett WC, Holmes MD. Diet Quality Is Associated with the Risk of Estrogen Receptor-Negative Breast Cancer in Postmenopausal Women. *J Nutr* (2006) 136(2):466-72. doi: 10.1093/jn/136.2.466.
119. Fung TT, Hu FB, Schulze M, Pollak M, Wu T, Fuchs CS, et al. A Dietary Pattern That Is Associated with C-Peptide and Risk of Colorectal Cancer in Women. *Cancer Causes Control* (2012) 23(6):959-65. doi: 10.1007/s10552-012-9969-y.
120. Fung TT, Isanaka S, Hu FB, Willett WC. International Food Group-Based Diet Quality and Risk of Coronary Heart Disease in Men and Women. *Am J Clin Nutr* (2018) 107(1):120-9. doi: 10.1093/ajcn/nqx015.
121. Fung TT, Pan A, Hou T, Chiuve SE, Tobias DK, Mozaffarian D, et al. Long-Term Change in Diet Quality Is Associated with Body Weight Change in Men and Women. *J Nutr* (2015) 145(8):1850-6. doi: 10.3945/jn.114.208785.
122. Fung TT, Pan A, Hou T, Mozaffarian D, Rexrode KM, Willett WC, et al. Food Quality Score and the Risk of Coronary Artery Disease: A Prospective Analysis in 3 Cohorts. *Am J Clin Nutr* (2016) 104(1):65-72. doi: 10.3945/ajcn.116.130393.
123. Fung TT, Rexrode KM, Mantzoros CS, Manson JE, Willett WC, Hu FB. Mediterranean Diet and Incidence of and Mortality from Coronary Heart Disease and Stroke in Women. *Circulation* (2009) 119(8):1093-100. doi: 10.1161/CIRCULATIONAHA.108.816736.
124. Fung TT, Schulze MB, Hu FB, Hankinson SE, Holmes MD. A Dietary Pattern Derived to Correlate with Estrogens and Risk of Postmenopausal Breast Cancer. *Breast Cancer Res Treat* (2012) 132(3):1157-62. doi: 10.1007/s10549-011-1942-z.
125. Fung TT, Schulze M, Manson JE, Willett WC, Hu FB. Dietary Patterns, Meat Intake, and the Risk of Type 2 Diabetes in Women. *Arch Intern Med* (2004) 164(20):2235-40. doi: 10.1001/archinte.164.20.2235.
126. Fung TT, Willett WC, Stampfer MJ, Manson JE, Hu FB. Dietary Patterns and the Risk of Coronary Heart Disease in Women. *Arch Intern Med* (2001) 161(15):1857-62. doi: 10.1001/archinte.161.15.1857.
127. Galbete C, Kröger J, Jannasch F, Iqbal K, Schwingshackl L, Schwedhelm C, et al. Nordic Diet, Mediterranean Diet, and the Risk of Chronic Diseases: The Epic-Potsdam Study. *BMC Med* (2018) 16(1). doi: 10.1186/s12916-018-1082-y.
128. Garcia-Arellano A, Martínez-González MA, Ramallal R, Salas-Salvadó J, Hébert JR, Corella D, et al. Dietary Inflammatory Index and All-Cause Mortality in Large Cohorts: The Sun and Predimed Studies. *Clin Nutr* (2018). doi: 10.1016/j.clnu.2018.05.003.
129. Gardener H, Wright CB, Gu Y, Demmer RT, Boden-Albala B, Elkind MSV, et al. Mediterranean-Style Diet and Risk of Ischemic Stroke, Myocardial Infarction, and Vascular Death: The Northern Manhattan Study. *Am J Clin Nutr* (2011) 94(6):1458-64. doi: 10.3945/ajcn.111.012799.
130. Garralda-Del-Villar M, Carlos-Chillerón S, Diaz-Gutierrez J, Ruiz-Canela M, Gea A, Martínez-González MA, et al. Healthy Lifestyle and Incidence of Metabolic Syndrome in the Sun Cohort. *Nutrients* (2019). doi: 10.3390/nu11010065.

131. George SM, Ballard R, Shikany JM, Crane TE, Neuhouser ML. A Prospective Analysis of Diet Quality and Endometrial Cancer among 84,415 Postmenopausal Women in the Women's Health Initiative. *Ann Epidemiol* (2015) 25(10):788-93. doi: 10.1016/j.annepidem.2015.05.009.
132. George SM, Ballard-Barbash R, Manson JE, Reedy J, Shikany JM, Subar AF, et al. Comparing Indices of Diet Quality with Chronic Disease Mortality Risk in Postmenopausal Women in the Women's Health Initiative Observational Study: Evidence to Inform National Dietary Guidance. *Am J Epidemiol* (2014) 180(6):616-25. doi: 10.1093/aje/kwu173.
133. Gómez-Donoso C, Martínez-González MA, Gea A, Murphy KJ, Parletta N, Bes-Rastrollo M. A Food-Based Score and Incidence of Overweight/Obesity: The Dietary Obesity-Prevention Score (Dos). *Clin Nutr* (2018). doi: 10.1016/j.clnu.2018.11.003.
134. Granic A, Andel R, Dahl AK, Gatz M, Pedersen NL. Midlife Dietary Patterns and Mortality in the Population-Based Study of Swedish Twins. *J Epidemiol Community Health* (2013) 67(7):578-86. doi: 10.1136/jech-2012-201780.
135. Guallar-Castillón P, Rodríguez-Artalejo F, Tormo MJ, Sánchez MJ, Rodríguez L, Quirós JR, et al. Major Dietary Patterns and Risk of Coronary Heart Disease in Middle-Aged Persons from a Mediterranean Country: The Epic-Spain Cohort Study. *Nutr Metab Cardiovasc Dis* (2012) 22(3):192-9. doi: 10.1016/j.numecd.2010.06.004.
136. Guinter MA, McLain AC, Merchant AT, Sandler DP, Steck SE. A Dietary Pattern Based on Estrogen Metabolism Is Associated with Breast Cancer Risk in a Prospective Cohort of Postmenopausal Women. *Int J Cancer* (2018) 143(3):580-90. doi: 10.1002/ijc.31387.
137. Guinter MA, Sandler DP, McLain AC, Merchant AT, Steck SE. An Estrogen-Related Dietary Pattern and Postmenopausal Breast Cancer Risk in a Cohort of Women with a Family History of Breast Cancer. *Cancer Epidemiol Biomarkers Prev* (2018) 27(10):1223-6. doi: 10.1158/1055-9965.EPI-18-0514.
138. Gunge VB, Andersen I, Kyrø C, Hansen CP, Dahm CC, Christensen J, et al. Adherence to a Healthy Nordic Food Index and Risk of Myocardial Infarction in Middle-Aged Danes: The Diet, Cancer and Health Cohort Study. *Eur J Clin Nutr* (2017) 71(5):652-8. doi: 10.1038/ejcn.2017.1.
139. Hansen CP, Overvad K, Kyrø C, Olsen A, Tjønneland A, Johnsen Sø P, et al. Adherence to a Healthy Nordic Diet and Risk of Stroke: A Danish Cohort Study. *Stroke* (2017) 48(2):259-64. doi: 10.1161/STROKEAHA.116.015019.
140. Hansen CP, Overvad K, Tetens I, Tjonneland A, Parner ET, Jakobsen MU, et al. Adherence to the Danish Food-Based Dietary Guidelines and Risk of Myocardial Infarction: A Cohort Study. *Public Health Nutr* (2018) 21(7):1286-96. doi: 10.1017/S1368980017003822.
141. Hansen SH, Overvad K, Hansen CP, Dahm CC. Adherence to National Food-Based Dietary Guidelines and Incidence of Stroke: A Cohort Study of Danish Men and Women. *PLoS ONE* (2018) 13(10):e0206242-e. doi: 10.1371/journal.pone.0206242.
142. Haridass V, Ziogas A, Neuhausen SL, Anton-Culver H, Odegaard AO. Diet Quality Scores Inversely Associated with Postmenopausal Breast Cancer Risk Are Not Associated with Premenopausal Breast Cancer Risk in the California Teachers Study. *J Nutr* (2018) 148(11):1830-7. doi: 10.1093/jn/nxy187.
143. Haring B, Crandall CJ, Wu C, LeBlanc ES, Shikany JM, Carbone L, et al. Dietary Patterns and Fractures in Postmenopausal Women: Results from the Women's Health Initiative. *JAMA Intern Med* (2016) 176(5):645-52. doi: 10.1001/jamainternmed.2016.0482.
144. Harmon BE, Boushey CJ, Shvetsov YB, Ettienne R, Reedy J, Wilkens LR, et al. Associations of Key Diet-Quality Indexes with Mortality in the Multiethnic Cohort: The Dietary Patterns Methods Project. *Am J Clin Nutr* (2015) 101(3):587-97. doi: 10.3945/ajcn.114.090688.

145. Harnack L, Nicodemus K, Jacobs Jr DR, Folsom AR. An Evaluation of the Dietary Guidelines for Americans in Relation to Cancer Occurrence. *Am J Clin Nutr* (2002) 76(4):889-96. doi: 10.1093/ajcn/76.4.889.
146. Harrington JM, Dahly DL, Fitzgerald AP, Gilthorpe MS, Perry IJ. Capturing Changes in Dietary Patterns among Older Adults: A Latent Class Analysis of an Ageing Irish Cohort. *Public Health Nutr* (2014) 17(12):2674-86. doi: 10.1017/S1368980014000111.
147. Harris HR, Bergkvist L, Wolk A. An Estrogen-Associated Dietary Pattern and Breast Cancer Risk in the Swedish Mammography Cohort. *Int J Cancer* (2015) 137(9):2149-54. doi: 10.1002/ijc.29586.
148. Harriss LR, English DR, Powles J, Giles GG, Tonkin AM, Hodge AM, et al. Dietary Patterns and Cardiovascular Mortality in the Melbourne Collaborative Cohort Study. *Am J Clin Nutr* (2007) 86(1):221-9. doi: 10.1093/ajcn/86.1.221.
149. Hassannejad R, Kazemi I, Sadeghi M, Mohammadifard N, Roohafza H, Sarrafzadegan N, et al. Longitudinal Association of Metabolic Syndrome and Dietary Patterns: A 13-Year Prospective Population-Based Cohort Study. *Nutr Metab Cardiovasc Dis* (2018) 28(4):352-60. doi: 10.1016/j.numecd.2017.10.025.
150. Heidemann C, Hoffmann K, Spranger J, Klipstein-Grobusch K, Möhlig M, Pfeiffer AFH, et al. A Dietary Pattern Protective against Type 2 Diabetes in the European Prospective Investigation into Cancer and Nutrition (Epic) - Potsdam Study Cohort. *Diabetologia* (2005) 48(6):1126-34. doi: 10.1007/s00125-005-1743-1.
151. Heidemann C, Schulze MB, Franco OH, Dam RMv, Mantzoros CS, Hu FB. Dietary Patterns and Risk of Mortality from Cardiovascular Disease, Cancer, and All Causes in a Prospective Cohort of Women. *Circulation* (2008) 118(3):230-7. doi: 10.1161/CIRCULATIONAHA.108.771881.
152. Hengeveld LM, Wijnhoven HAH, Olthof MR, Brouwer IA, Harris TB, Kritchevsky SB, et al. Prospective Associations of Poor Diet Quality with Long-Term Incidence of Protein-Energy Malnutrition in Community-Dwelling Older Adults: The Health, Aging, and Body Composition (Health Abc) Study. *Am J Clin Nutr* (2018) 107(2):155-64. doi: 10.1093/ajcn/nqx020.
153. Héroux M, Janssen I, Lam M, Lee DC, Hebert JR, Sui XM, et al. Dietary Patterns and the Risk of Mortality: Impact of Cardiorespiratory Fitness. *Int J Epidemiol* (2010) 39(1):197-209. doi: 10.1093/ije/dyp191.
154. Hidaka BH, Kimler BF, Fabian CJ, Carlson SE. An Empirically Derived Dietary Pattern Associated with Breast Cancer Risk Is Validated in a Nested Case-Control Cohort from a Randomized Primary Prevention Trial. *Clin Nutr ESPEN* (2017) 17:8-17. doi: 10.1016/j.clnesp.2016.10.008.
155. Hirko KA, Willett WC, Hankinson SE, Rosner BA, Beck AH, Tamimi RM, et al. Healthy Dietary Patterns and Risk of Breast Cancer by Molecular Subtype. *Breast Cancer Res Treat* (2016) 155(3):579-88. doi: 10.1007/s10549-016-3706-2.
156. Hlebowicz J, Drake I, Gullberg B, Sonestedt E, Wallström P, Persson M, et al. A High Diet Quality Is Associated with Lower Incidence of Cardiovascular Events in the Malmö Diet and Cancer Cohort. *PLoS ONE* (2013) 8(8). doi: 10.1371/journal.pone.0071095.
157. Hlebowicz J, Persson M, Gullberg B, Sonestedt E, Wallström P, Drake I, et al. Food Patterns, Inflammation Markers and Incidence of Cardiovascular Disease: The Malmö Diet and Cancer Study. *J Intern Med* (2011) 270(4):365-76. doi: 10.1111/j.1365-2796.2011.02382.x.
158. Hodge AM, Bassett JK, Dugué PA, Shivappa N, Hébert JR, Milne RL, et al. Dietary Inflammatory Index or Mediterranean Diet Score as Risk Factors for Total and Cardiovascular Mortality. *Nutr Metab Cardiovasc Dis* (2018) 28(5):461-9. doi: 10.1016/j.numecd.2018.01.010.

159. Hodge AM, Bassett JK, Shivappa N, Hébert JR, English DR, Giles GG, et al. Dietary Inflammatory Index, Mediterranean Diet Score, and Lung Cancer: A Prospective Study. *Cancer Causes Control* (2016) 27(7):907-17. doi: 10.1007/s10552-016-0770-1.
160. Hodge AM, English DR, O'Dea K, Giles GG. Dietary Patterns and Diabetes Incidence in the Melbourne Collaborative Cohort Study. *Am J Epidemiol* (2007) 165(6):603-10. doi: 10.1093/aje/kwk061.
161. Hoevenaar-Blom MP, Nooyens ACJ, Kromhout D, Spijkerman AMW, Beulens JWJ, Schouw YTvd, et al. Mediterranean Style Diet and 12-Year Incidence of Cardiovascular Diseases: The Epic-Nl Cohort Study. *PLoS ONE* (2012) 7(9):e45458-e. doi: 10.1371/journal.pone.0045458.
162. Hosseini-Esfahani F, Mirmiran P, Daneshpour MS, Mehrabi Y, Hedayati M, Zarkesh M, et al. Western Dietary Pattern Interaction with Apoc3 Polymorphism in the Risk of Metabolic Syndrome: Tehran Lipid and Glucose Study. *J Nutrigenet Nutrigenomics* (2014) 7(2):105-17. doi: 10.1159/000365445.
163. Hu FB, Rimm EB, Stampfer MJ, Ascherio A, Spiegelman D, Willett WC. Prospective Study of Major Dietary Patterns and Risk of Coronary Heart Disease in Men. *Am J Clin Nutr* (2000) 72(4):912-21. doi: 10.1093/ajcn/72.4.912.
164. Huijbregts P, Feskens E, Rasanen L, Fidanza F, Nissinen A, Menotti A, et al. Dietary Pattern and 20 Year Mortality in Elderly Men in Finland, Italy, and the Netherlands: Longitudinal Cohort Study. *BMJ* (1997) 315(7099):13-7. doi: 10.1136/bmj.315.7099.13.
165. Inoue-Choi M, Flood A, Robien K, Anderson K. Nutrients, Food Groups, Dietary Patterns, and Risk of Pancreatic Cancer in Postmenopausal Women. *Cancer Epidemiol Biomarkers Prev* (2011) 20(4):711-4. doi: 10.1158/1055-9965.EPI-11-0026.
166. Jacobs S, Boushey CJ, Franke AA, Shvetsov YB, Monroe KR, Haiman CA, et al. A Priori-Defined Diet Quality Indices, Biomarkers and Risk for Type 2 Diabetes in Five Ethnic Groups: The Multiethnic Cohort. *Br J Nutr* (2017) 118(4):312-20. doi: 10.1017/S0007114517002033.
167. Jacobs S, Harmon BE, Boushey CJ, Morimoto Y, Wilkens LR, Marchand Ll, et al. A Priori-Defined Diet Quality Indexes and Risk of Type 2 Diabetes: The Multiethnic Cohort. *Diabetologia* (2015) 58(1):98-112. doi: 10.1007/s00125-014-3404-8.
168. Jacobs S, Harmon BE, Ollberding NJ, Wilkens LR, Monroe KR, Kolonel LN, et al. Among 4 Diet Quality Indexes, Only the Alternate Mediterranean Diet Score Is Associated with Better Colorectal Cancer Survival and Only in African American Women in the Multiethnic Cohort. *J Nutr* (2016) 146(9):1746-55. doi: 10.3945/jn.116.234237.
169. Jones NRV, Forouhi NG, Khaw KT, Wareham NJ, Monsivais P. Accordance to the Dietary Approaches to Stop Hypertension Diet Pattern and Cardiovascular Disease in a British, Population-Based Cohort. *Eur J Epidemiol* (2018) 33(2):235-44. doi: 10.1007/s10654-017-0354-8.
170. Jones P, Cade JE, Evans CEL, Hancock N, Greenwood DC. The Mediterranean Diet and Risk of Colorectal Cancer in the Uk Women's Cohort Study. *Int J Epidemiol* (2017) 46(6):1786-96. doi: 10.1093/ije/dyx155.
171. Judd SE, Gutiérrez OM, Newby PK, Howard G, Howard VJ, Locher JL, et al. Dietary Patterns Are Associated with Incident Stroke and Contribute to Excess Risk of Stroke in Black Americans. *Stroke* (2013) 44(12):3305-11. doi: 10.1161/STROKEAHA.113.002636.
172. Julia C, Ducrot P, Lassale C, Fézeu L, Méjean C, Péneau S, et al. Prospective Associations between a Dietary Index Based on the British Food Standard Agency Nutrient Profiling System and 13-Year Weight Gain in the Su.Vi.Max Cohort. *Prev Med* (2015) 81:189-94. doi: 10.1016/j.ypmed.2015.08.022.
173. Kaluza J, Håkansson N, Brzozowska A, Wolk A. Diet Quality and Mortality: A Population-Based Prospective Study of Men. *Eur J Clin Nutr* (2009) 63(4):451-7. doi: 10.1038/sj.ejcn.1602968.

174. Kaluza J, Håkansson N, Harris HR, Orsini N, Michaëlsson K, Wolk A. Influence of Anti-Inflammatory Diet and Smoking on Mortality and Survival in Men and Women: Two Prospective Cohort Studies. *J Intern Med* (2018). doi: 10.1111/joim.12823.
175. Kane-Diallo A, Srour B, Sellem L, Deschasaux M, Latino-Martel P, Hercberg S, et al. Association between a Pro Plant-Based Dietary Score and Cancer Risk in the Prospective Nutrinet-Santé Cohort. *Int J Cancer* (2018) 143(9):2168-76. doi: 10.1002/ijc.31593.
176. Kant AK, Graubard BI, Schatzkin A. Dietary Patterns Predict Mortality in a National Cohort: The National Health Interview Surveys, 1987 and 1992. *J Nutr* (2004) 134(7):1793-9. doi: 10.1093/jn/134.7.1793.
177. Kant AK, Schatzkin A, Graubard BI, Schairer C. A Prospective Study of Diet Quality and Mortality in Women. *JAMA* (2000) 283(16):2109-15. doi: 10.1001/jama.283.16.2109.
178. Kesse E, Clavel-Chapelon F, Boutron-Ruault MC. Dietary Patterns and Risk of Colorectal Tumors: A Cohort of French Women of the National Education System (E3n). *Am J Epidemiol* (2006) 164(11):1085-93. doi: 10.1093/aje/kwj324.
179. Kesse-Guyot E, Ahluwalia N, Lassale C, Hercberg S, Fezeu L, Lairon D. Adherence to Mediterranean Diet Reduces the Risk of Metabolic Syndrome: A 6-Year Prospective Study. *Nutr Metab Cardiovasc Dis* (2013) 23(7):677-83. doi: 10.1016/j.numecd.2012.02.005.
180. Kim M, Sasaki S, Otani T, Tsugane S. Dietary Patterns and Subsequent Colorectal Cancer Risk by Subsite: A Prospective Cohort Study. *Int J Cancer* (2005) 115(5):790-8. doi: 10.1002/ijc.20943.
181. Kimokoti RW, Gona P, Zhu L, Newby PK, Millen BE, Brown LS, et al. Dietary Patterns of Women Are Associated with Incident Abdominal Obesity but Not Metabolic Syndrome. *J Nutr* (2012) 142(9):1720-7. doi: 10.3945/jn.112.162479.
182. Knoops KTB, de Groot LCPGM, Kromhout D, Perrin AE, Moreiras-Varela O, Menotti A, et al. Mediterranean Diet, Lifestyle Factors, and 10-Year Mortality in Elderly European Men and Women: The Hale Project. *JAMA* (2004) 292(12):1433-9. doi: 10.1001/jama.292.12.1433.
183. Knoops KTB, de Groot LC, Fidanza F, Alberti-Fidanza A, Kromhout D, Staveren WAv. Comparison of Three Different Dietary Scores in Relation to 10-Year Mortality in Elderly European Subjects: The Hale Project. *Eur J Clin Nutr* (2006) 60(6):746-55. doi: 10.1038/sj.ejcn.1602378.
184. Kojima R, Okada E, Ukawa S, Mori M, Wakai K, Date C, et al. Dietary Patterns and Breast Cancer Risk in a Prospective Japanese Study. *Breast Cancer* (2017) 24(1):152-60. doi: 10.1007/s12282-016-0689-0.
185. Koloverou E, Panagiotakos DB, Georgousopoulou EN, Grekas A, Christou A, Chatzigeorgiou M, et al. Dietary Patterns and 10-Year (2002-2012) Incidence of Type 2 Diabetes: Results from the Attica Cohort Study. *Rev Diabet Stud* (2016) 13(4):246-56. doi: 10.1900/RDS.2016.13.246.
186. Koloverou E, Panagiotakos DB, Pitsavos C, Chrysohoou C, Georgousopoulou EN, Grekas A, et al. Adherence to Mediterranean Diet and 10-Year Incidence (2002-2012) of Diabetes: Correlations with Inflammatory and Oxidative Stress Biomarkers in the Attica Cohort Study. *Diabetes Metab Res Rev* (2016) 32(1):73-81. doi: 10.1002/dmrr.2672.
187. Kouris-Blazos A, Gnardellis C, Wahlqvist ML, Trichopoulos D, Lukito W, Trichopoulou A. Are the Advantages of the Mediterranean Diet Transferable to Other Populations? A Cohort Study in Melbourne, Australia. *Br J Nutr* (1999) 82(1):57-61. doi: 10.1017/s0007114599001129.
188. Kouvari M, Panagiotakos DB, Yannakoulia M, Georgousopoulou E, Critselis E, Chrysohoou C, et al. Transition from Metabolically Benign to Metabolically Unhealthy Obesity and 10-Year Cardiovascular Disease Incidence: The Attica Cohort Study. *Metab Clin Exp* (2019) 93:18-24. doi: 10.1016/j.metabol.2019.01.003.

189. The InterAct Consortium, Kröger J, Schulze MB. Adherence to Predefined Dietary Patterns and Incident Type 2 Diabetes in European Populations: Epic-Interact Study. *Diabetologia* (2014) 57(2):321-33. doi: 10.1007/s00125-013-3092-9.
190. Kromhout D, Menotti A, Alberti-Fidanza A, Puddu PE, Hollman P, Kafatos A, et al. Comparative Ecologic Relationships of Saturated Fat, Sucrose, Food Groups, and a Mediterranean Food Pattern Score to 50-Year Coronary Heart Disease Mortality Rates among 16 Cohorts of the Seven Countries Study. *Eur J Clin Nutr* (2018) 72(8):1103-10. doi: 10.1038/s41430-018-0183-1.
191. Kumagai Y, Chou W, Tomata Y, Sugawara Y, Kakizaki M, Nishino Y, et al. Dietary Patterns and Colorectal Cancer Risk in Japan: The Ohsaki Cohort Study. *Cancer Causes Control* (2014) 25(6):727-36. doi: 10.1007/s10552-014-0375-5.
192. Kurotani K, Akter S, Kashino I, Goto A, Mizoue T, Noda M, et al. Quality of Diet and Mortality among Japanese Men and Women: Japan Public Health Center Based Prospective Study. *BMJ* (2016) 352(I1209). doi: 10.1136/bmj.i1209.
193. Kyro C, Skeie G, Loft S, Overvad K, Christensen J, Tjonneland A, et al. Adherence to a Healthy Nordic Food Index Is Associated with a Lower Incidence of Colorectal Cancer in Women: The Diet, Cancer and Health Cohort Study. *Br J Nutr* (2013) 109(5):920-7. doi: 10.1017/S0007114512002085.
194. Lachman S, Peters RJG, Lentjes MAH, Mulligan AA, Luben RN, Wareham NJ, et al. Ideal Cardiovascular Health and Risk of Cardiovascular Events in the Epic-Norfolk Prospective Population Study. *Eur J Prev Cardiol* (2015) 23(9):986-94. doi: 10.1177/2047487315602015.
195. Lacoppidan SA, Kyrø C, Loft S, Helnæs A, Christensen J, Hansen CP, et al. Adherence to a Healthy Nordic Food Index Is Associated with a Lower Risk of Type-2 Diabetes - the Danish Diet, Cancer and Health Cohort Study. *Nutrients* (2015) 7(10):8633-44. doi: 10.3390/nu7105418.
196. Lagiou P, Trichopoulos D, Sandin S, Lagiou A, Mucci L, Wolk A, et al. Mediterranean Dietary Pattern and Mortality among Young Women: A Cohort Study in Sweden. *Br J Nutr* (2006) 96(2):384-92. doi: 10.1079/BJN20061824.
197. Langsetmo L, Hanley DA, Prior JC, Barr SI, Anastassiades T, Towheed T, et al. Dietary Patterns and Incident Low-Trauma Fractures in Postmenopausal Women and Men Aged ≥50 Y: A Population-Based Cohort Study. *Am J Clin Nutr* (2011) 93(1):192-9. doi: 10.3945/ajcn.110.002956.
198. Langsetmo L, Poliquin S, Hanley DA, Prior JC, Barr S, Anastassiades T, et al. Dietary Patterns in Canadian Men and Women Ages 25 and Older: Relationship to Demographics, Body Mass Index, and Bone Mineral Density. *BMC Musculoskelet Disord* (2010) 11. doi: 10.1186/1471-2474-11-20.
199. Larsson SC, Åkesson A, Wolk A. Overall Diet Quality and Risk of Stroke: A Prospective Cohort Study in Women. *Atherosclerosis* (2014) 233(1):27-9. doi: 10.1016/j.atherosclerosis.2013.11.072.
200. Larsson SC, Åkesson A, Wolk A. Healthy Diet and Lifestyle and Risk of Stroke in a Prospective Cohort of Women. *Neurology* (2014) 83(19):1699-704. doi: 10.1212/WNL.0000000000000954.
201. Larsson SC, Tektonidis TG, Gigante B, Akesson A, Wolk A. Healthy Lifestyle and Risk of Heart Failure. *Circ Heart Fail* (2016) 9(4). doi: 10.1161/CIRCHEARTFAILURE.115.002855.
202. Larsson SC, Wallin A, Wolk A. Dietary Approaches to Stop Hypertension Diet and Incidence of Stroke: Results from 2 Prospective Cohorts. *Stroke* (2016) 47(4):986-90. doi: 10.1161/STROKEAHA.116.012675.
203. Larsson SC, Wolk A, Bäck M. Dietary Patterns, Food Groups, and Incidence of Aortic Valve Stenosis: A Prospective Cohort Study. *Int J Cardiol* (2018) 283:184-8. doi: 10.1016/j.ijcard.2018.11.007.

204. Lassale C, Fezeu L, Andreeva VA, Hercberg S, Kengne AP, Czernichow S, et al. Association between Dietary Scores and 13-Year Weight Change and Obesity Risk in a French Prospective Cohort. *Int J Obes* (2012) 36(11):1455-62. doi: 10.1038/ijo.2011.264.
205. Lassale C, Gunter MJ, Romaguera D, Peelen LM, Van Der Schouw YT, Beulens JWJ, et al. Diet Quality Scores and Prediction of All-Cause, Cardiovascular and Cancer Mortality in a Pan-European Cohort Study. *PLoS ONE* (2016) 11(7). doi: 10.1371/journal.pone.0159025.
206. Lavalette C, Adjibade M, Srour B, Sellem L, Fiolet T, Hercberg S, et al. Cancer-Specific and General Nutritional Scores and Cancer Risk: Results from the Prospective Nutrinet-Santé Cohort. *Cancer Res* (2018) 78(15):4427-35. doi: 10.1158/0008-5472.CAN-18-0155.
207. Leary SD, Lawlor DA, Davey Smith G, Brion MJ, Ness AR. Behavioural Early-Life Exposures and Body Composition at Age 15 Years. *Nutr Diabetes* (2015) 5(1). doi: 10.1038/nutd.2014.47.
208. Lee MS, Huang YC, Su HH, Lee MZ, Wahlqvist ML. A Simple Food Quality Index Predicts Mortality in Elderly Taiwanese. *J Nutr Health Aging* (2011) 15(10):815-21. doi: 10.1007/s12603-011-0081-x.
209. Leenders M, Siersema PD, Overvad K, Tjønneland A, Olsen A, Boutron-Ruault MC, et al. Subtypes of Fruit and Vegetables, Variety in Consumption and Risk of Colon and Rectal Cancer in the European Prospective Investigation into Cancer and Nutrition. *Int J Cancer* (2015) 137(11):2705-14. doi: 10.1002/ijc.29640.
210. Lemming EW, Byberg L, Melhus H, Wolk A, Michaëlsson K. Long-Term a Posteriori Dietary Patterns and Risk of Hip Fractures in a Cohort of Women. *Eur J Epidemiol* (2017) 32(7):605-16. doi: 10.1007/s10654-017-0267-6.
211. Lemming EW, Byberg L, Wolk A, Michaëlsson K. A Comparison between Two Healthy Diet Scores, the Modified Mediterranean Diet Score and the Healthy Nordic Food Index, in Relation to All-Cause and Cause-Specific Mortality. *Br J Nutr* (2018) 119(7):836-46. doi: 10.1017/S0007114518000387.
212. Letois F, Mura T, Scali J, Gutierrez LA, Féart C, Berr C. Nutrition and Mortality in the Elderly over 10 Years of Follow-Up: The Three-City Study. *Br J Nutr* (2016) 116(5):882-9. doi: 10.1017/S000711451600266X.
213. Levitan EB, Wolk A, Mittleman MA. Relation of Consistency with the Dietary Approaches to Stop Hypertension Diet and Incidence of Heart Failure in Men Aged 45 to 79 Years. *Am J Cardiol* (2009) 104(10):1416-20. doi: 10.1016/j.amjcard.2009.06.061.
214. Levitan EB, Wolk A, Mittleman MA. Consistency with the Dash Diet and Incidence of Heart Failure. *Arch Intern Med* (2009) 169(9):851-7. doi: 10.1001/archinternmed.2009.56.
215. Ley SH, Pan A, Li Y, Manson JE, Willett WC, Sun Q, et al. Changes in Overall Diet Quality and Subsequent Type 2 Diabetes Risk: Three U.S. Prospective Cohorts. *Diabetes Care* (2016) 39(11):2011-8. doi: 10.2337/dc16-0574.
216. Li M, Shi Z. Dietary Pattern During 1991–2011 and Its Association with Cardio Metabolic Risks in Chinese Adults: The China Health and Nutrition Survey. *Nutrients* (2017). doi: 10.3390/nu9111218.
217. Li P, Zhang M, Zhu Y, Liu W, Zhang Y, Gao Y, et al. Dietary Patterns and Changes in Cardiovascular Risk Factors in Apparently Healthy Chinese Women: A Longitudinal Study. *J Clin Biochem Nutr* (2016) 58(3):232-9. doi: 10.3164/jcbn.15-78.
218. Li W, Park Y, Wu JW, Ren J, Goldstein AM, Taylor PR, et al. Index-Based Dietary Patterns and Risk of Esophageal and Gastric Cancer in a Large Cohort Study. *Clin Gastroenterol Hepatol* (2013) 11(9):1130-6. doi: 10.1016/j.cgh.2013.03.023.

219. Li WQ, Park YK, McGlynn KA, Hollenbeck AR, Taylor PR, Goldstein AM, et al. Index-Based Dietary Patterns and Risk of Incident Hepatocellular Carcinoma and Mortality from Chronic Liver Disease in a Prospective Study. *Hepatology* (2014) 60(2):588-97. doi: 10.1002/hep.27160.
220. Li WQ, Park Y, Wu JW, Goldstein AM, Taylor PR, Hollenbeck AR, et al. Index-Based Dietary Patterns and Risk of Head and Neck Cancer in a Large Prospective Study. *Am J Clin Nutr* (2014) 99(3):559-66. doi: 10.3945/ajcn.113.073163.
221. Li Y, Roswall N, Sandin S, Ström P, Adami HO, Weiderpass E. Adherence to a Healthy Nordic Food Index and Breast Cancer Risk: Results from a Swedish Cohort Study. *Cancer Causes Control* (2015) 26(6):893-902. doi: 10.1007/s10552-015-0564-x.
222. Li Y, Roswall N, Ström P, Sandin S, Adami HO, Weiderpass E. Mediterranean and Nordic Diet Scores and Long-Term Changes in Body Weight and Waist Circumference: Results from a Large Cohort Study. *Br J Nutr* (2015) 114(12):2093-102. doi: 10.1017/S0007114515003840.
223. Li Y, Pan A, Wang DD, Liu X, Dhana K, Franco OH, et al. Impact of Healthy Lifestyle Factors on Life Expectancies in the Us Population. *Circulation* (2018) 138(4):345-55. doi: 10.1161/CIRCULATIONAHA.117.032047.
224. Liese AD, Krebs-Smith SM, Subar AF, George SM, Harmon BE, Neuhouser ML, et al. The Dietary Patterns Methods Project: Synthesis of Findings across Cohorts and Relevance to Dietary Guidance. *J Nutr* (2015) 145(3):393-402. doi: 10.3945/jn.114.205336.
225. Liese AD, Weis KE, Schulz M, Tooze JA. Food Intake Patterns Associated with Incident Type 2 Diabetes: The Insulin Resistance Atherosclerosis Study. *Diabetes Care* (2009) 32(2):263-8. doi: 10.2337/dc08-1325.
226. Lim J, Lee Y, Shin S, Lee H, Kim CE, Lee J, et al. An Association between Diet Quality Index for Koreans (Dqi-K) and Total Mortality in Health Examinees Gem (Hexa-G) Study. *Nutr Res Pract* (2018) 12(3):258-64. doi: 10.4162/nrp.2018.12.3.258.
227. Limongi F, Noale M, Gesmundo A, Crepaldi G, Maggi S. Adherence to the Mediterranean Diet and All-Cause Mortality Risk in an Elderly Italian Population: Data from the Ilsa Study. *J Nutr Health Aging* (2017) 21(5):505-13. doi: 10.1007/s12603-016-0808-9.
228. Link LB, Canchola AJ, Bernstein L, Clarke CA, Stram DO, Ursin G, et al. Dietary Patterns and Breast Cancer Risk in the California Teachers Study Cohort. *Am J Clin Nutr* (2013) 98(6):1524-32. doi: 10.3945/ajcn.113.061184.
229. Liu L, Nishihara R, Qian Z, Tabung FK, Nevo D, Zhang X, et al. Association between Inflammatory Diet Pattern and Risk of Colorectal Carcinoma Subtypes Classified by Immune Responses to Tumor. *Gastroenterology* (2017) 153(6):1517-30.e14. doi: 10.1053/j.gastro.2017.08.045.
230. López-Laguna N, Martínez-González MA, Toledo E, Babio N, Sorlí JV, Ros E, et al. Risk of Peripheral Artery Disease According to a Healthy Lifestyle Score: The Predimed Study. *Atherosclerosis* (2018) 275:133-40. doi: 10.1016/j.atherosclerosis.2018.05.049.
231. Lutsey PL, Steffen LM, Stevens J. Dietary Intake and the Development of the Metabolic Syndrome: The Atherosclerosis Risk in Communities Study. *Circulation* (2008) 117(6):754-61. doi: 10.1161/CIRCULATIONAHA.107.716159.
232. Ma Y, Yang W, Simon TG, Smith-Warner SA, Fung TT, Sui J, et al. Dietary Patterns and Risk of Hepatocellular Carcinoma among U.S. Men and Women. *Hepatology* (2019). doi: 10.1002/hep.30362.
233. Makambi KH, Agurs-Collins T, Bright-Gbebry M, Rosenberg L, Palmer JR, Adams-Campbell LL. Dietary Patterns and the Risk of Colorectal Adenomas: The Black Women's Health Study. *Cancer Epidemiol Biomarkers Prev* (2011) 20(5):818-25. doi: 10.1158/1055-9965.EPI-10-1213.

234. Malekshah AFT, Zaroudi M, Etemadi A, Islami F, Sepanlou S, Sharafkhah M, et al. The Combined Effects of Healthy Lifestyle Behaviors on All-Cause Mortality: The Golestan Cohort Study. *Arch Iran Med* (2016) 19(11):752-61.
235. Malik VS, Fung TT, van Dam RM, Rimm EB, Rosner B, Hu FB. Dietary Patterns During Adolescence and Risk of Type 2 Diabetes in Middle-Aged Women. *Diabetes Care* (2012) 35(1):12-8. doi: 10.2337/dc11-0386.
236. Mandalazi E, Drake I, Wirfält E, Orho-Melander M, Sonestedt E. A High Diet Quality Based on Dietary Recommendations Is Not Associated with Lower Incidence of Type 2 Diabetes in the Malmö Diet and Cancer Cohort. *Int J Mol Sci* (2016) 17(6). doi: 10.3390/ijms17060901.
237. Mangano KM, Sahni S, Kiel DP, Tucker KL, Dufour AB, Hannan MT. Dietary Protein Is Associated with Musculoskeletal Health Independently of Dietary Pattern: The Framingham Third Generation Study. *Am J Clin Nutr* (2017) 105(3):714-22. doi: 10.3945/ajcn.116.136762.
238. Männistö S, Dixon LB, Balder HF, Virtanen MJ, Krogh V, Khani BR, et al. Dietary Patterns and Breast Cancer Risk: Results from Three Cohort Studies in the Dietscan Project. *Cancer Causes Control* (2005) 16(6):725-33. doi: 10.1007/s10552-005-1763-7.
239. Martínez-González MA, García-López M, Bes-Rastrollo M, Toledo E, Martínez-Lapiscina EH, Delgado-Rodriguez M, et al. Mediterranean Diet and the Incidence of Cardiovascular Disease: A Spanish Cohort. *Nutr Metab Cardiovasc Dis* (2011) 21(4):237-44. doi: 10.1016/j.numecd.2009.10.005.
240. Martínez-González MA, Zazpe I, Razquin C, Sánchez-Tainta A, Corella D, Salas-Salvadó J, et al. Empirically-Derived Food Patterns and the Risk of Total Mortality and Cardiovascular Events in the Predimed Study. *Clin Nutr* (2015) 34(5):859-67. doi: 10.1016/j.clnu.2014.09.006.
241. Martínez-González MÁ, Fuente-Arrillaga Cdl, Nunez-Cordoba JM, Basterra-Gortari FJ, Beunza JJ, Vazquez Z, et al. Adherence to Mediterranean Diet and Risk of Developing Diabetes: Prospective Cohort Study. *BMJ* (2008) 336:1348-51. doi: 10.1136/bmj.39561.501007.BE.
242. Maruyama K, Iso H, Date C, Kikuchi S, Watanabe Y, Wada Y, et al. Dietary Patterns and Risk of Cardiovascular Deaths among Middle-Aged Japanese: Jacc Study. *Nutr Metab Cardiovasc Dis* (2013) 23(6):519-27. doi: 10.1016/j.numecd.2011.10.007.
243. Masala G, Ceroti M, Pala V, Krogh V, Vineis P, Sacerdote C, et al. A Dietary Pattern Rich in Olive Oil and Raw Vegetables Is Associated with Lower Mortality in Italian Elderly Subjects. *Br J Nutr* (2007) 98(2):406-15. doi: 10.1017/S0007114507704981.
244. McCourt HJ, Draffin CR, Woodside JV, Cardwell CR, Young IS, Hunter SJ, et al. Dietary Patterns and Cardiovascular Risk Factors in Adolescents and Young Adults: The Northern Ireland Young Hearts Project. *Br J Nutr* (2014) 112(10):1685-98. doi: 10.1017/S0007114514002682.
245. McCullough ML, Feskanich D, Rimm EB, Giovannucci EL, Ascherio A, Variyam JN, et al. Adherence to the Dietary Guidelines for Americans and Risk of Major Chronic Disease in Men. *Am J Clin Nutr* (2000) 72(5):1223-31. doi: 10.1093/ajcn/72.5.1223.
246. McCullough ML, Willett WC. Evaluating Adherence to Recommended Diets in Adults: The Alternate Healthy Eating Index. *Public Health Nutr* (2006) 9(1A):152-7. doi: 10.1079/PHN2005938.
247. McNaughton SA, Bates CJ, Mishra GD. Diet Quality Is Associated with All-Cause Mortality in Adults Aged 65 Years and Older. *J Nutr* (2012) 142(2):320-5. doi: 10.3945/jn.111.148692.
248. Mehta RS, Nishihara R, Cao Y, Song M, Mima K, Qian ZR, et al. Association of Dietary Patterns with Risk of Colorectal Cancer Subtypes Classified by Fusobacterium Nucleatum in Tumor Tissue. *JAMA Oncol* (2017) 3(7):921-7. doi: 10.1001/jamaoncol.2016.6374.
249. Mehta RS, Song M, Nishihara R, Drew DA, Wu K, Qian ZR, et al. Dietary Patterns and Risk of Colorectal Cancer: Analysis by Tumor Location and Molecular Subtypes. *Gastroenterology* (2017) 152(8):1944-53.e1. doi: 10.1053/j.gastro.2017.02.015.

250. Melaku YA, Gill TK, Appleton SL, Taylor AW, Adams R, Shi Z. Prospective Associations of Dietary and Nutrient Patterns with Fracture Risk: A 20-Year Follow-up Study. *Nutrients* (2017). doi: 10.3390/nu9111198.
251. Mendez MA, Popkin BM, Jakszyn P, Berenguer A, Tormo MJ, Sanchéz MJ, et al. Adherence to a Mediterranean Diet Is Associated with Reduced 3-Year Incidence of Obesity. *J Nutr* (2006) 136(11):2934-8. doi: 10.1093/jn/136.11.2934.
252. Menotti A, Kromhout D, Blackburn H, Fidanza F, Buzina R, Nissinen A. Food Intake Patterns and 25-Year Mortality from Coronary Heart Disease: Cross-Cultural Correlations in the Seven Countries Study. The Seven Countries Study Research Group. *Eur J Epidemiol* (1999) 15(6):507-15. doi: 10.1023/a:1007529206050.
253. Menotti A, Puddu PE, Lanti M, Maiani G, Catasta G, Fidanza AA. Lifestyle Habits and Mortality from All and Specific Causes of Death: 40-Year Follow-up in the Italian Rural Areas of the Seven Countries Study. *J Nutr Health Aging* (2014) 18(3):314-21. doi: 10.1007/s12603-013-0392-1.
254. Mertens E, Markey O, Geleijnse JM, Givens DI, Lovegrove JA. Dietary Patterns in Relation to Cardiovascular Disease Incidence and Risk Markers in a Middle-Aged British Male Population: Data from the Caerphilly Prospective Study. *Nutrients* (2017). doi: 10.3390/nu9010075.
255. Meyer J, Döring A, Herder C, Roden M, Koenig W, Thorand B. Dietary Patterns, Subclinical Inflammation, Incident Coronary Heart Disease and Mortality in Middle-Aged Men from the Monica/Kora Augsburg Cohort Study. *Eur J Clin Nutr* (2011) 65(7):800-7. doi: 10.1038/ejcn.2011.37.
256. Michaud DS, Skinner HG, Wu K, Hu F, Giovannucci E, Willett WC, et al. Dietary Patterns and Pancreatic Cancer Risk in Men and Women. *J Natl Cancer Inst* (2005) 97(7):518-24. doi: 10.1093/jnci/dji094.
257. Michels KB, Wolk A. A Prospective Study of Variety of Healthy Foods and Mortality in Women. *Int J Epidemiol* (2002) 31(4):847-54. doi: 10.1093/ije/31.4.847.
258. Millen BE, Quatromoni PA, Pencina M, Kimokoti R, Nam BHO, Cobain S, et al. Unique Dietary Patterns and Chronic Disease Risk Profiles of Adult Men: The Framingham Nutrition Studies. *J Am Diet Assoc* (2005) 105(11):1723-34. doi: 10.1016/j.jada.2005.08.007.
259. Misirli G, Benetou V, Lagiou P, Bamia C, Trichopoulos D, Trichopoulou A. Relation of the Traditional Mediterranean Diet to Cerebrovascular Disease in a Mediterranean Population. *Am J Epidemiol* (2012) 176(12):1185-92. doi: 10.1093/aje/kws205.
260. Mohammadifard N, Talaei M, Sadeghi M, Oveisegharan S, Golshahi J, Esmaillzadeh A, et al. Dietary Patterns and Mortality from Cardiovascular Disease: Isfahan Cohort Study. *Eur J Clin Nutr* (2017) 71(2):252-8. doi: 10.1038/ejcn.2016.170.
261. Molina-Montes E, Sánchez MJ, Buckland G, Bueno-De-Mesquita HB, Weiderpass E, Amiano P, et al. Mediterranean Diet and Risk of Pancreatic Cancer in the European Prospective Investigation into Cancer and Nutrition Cohort. *Br J Cancer* (2017) 116(6):811-20. doi: 10.1038/bjc.2017.14.
262. Monjardino T, Lucas R, Ramos E, Barros H. Associations between a Priori-Defined Dietary Patterns and Longitudinal Changes in Bone Mineral Density in Adolescents. *Public Health Nutr* (2014) 17(1):195-205. doi: 10.1017/S1368980012004879.
263. Monjardino T, Lucas R, Ramos E, Lopes C, Gaio R, Barros H. Associations between a Posteriori Defined Dietary Patterns and Bone Mineral Density in Adolescents. *Eur J Nutr* (2015) 54(2):273-82. doi: 10.1007/s00394-014-0708-x.
264. Monma Y, Niu K, Iwasaki K, Tomita N, Nakaya N, Hozawa A, et al. Dietary Patterns Associated with Fall-Related Fracture in Elderly Japanese: A Population Based Prospective Study. *BMC Geriatr* (2010) 10:31. doi: 10.1186/1471-2318-10-31.

265. Montonen J, Knekt P, Härkänen T, Järvinen R, Heliövaara M, Aromaa A, et al. Dietary Patterns and the Incidence of Type 2 Diabetes. *Am J Epidemiol* (2005) 161(3):219-27. doi: 10.1093/aje/kwi039.
266. Morimoto A, Ohno Y, Tatsumi Y, Mizuno S, Watanabe S. Effects of Healthy Dietary Pattern and Other Lifestyle Factors on Incidence of Diabetes in a Rural Japanese Population. *Asia Pac J Clin Nutr* (2012) 21(4):601-8.
267. Moslehi N, Hosseini-Esfahani F, Hosseinpanah F, Mirmiran P, Azizi F. Patterns of Food Consumption and Risk of Type 2 Diabetes in an Iranian Population: A Nested Case-Control Study. *Nutr Diet* (2016) 73(2):169-76. doi: 10.1111/1747-0080.12189.
268. Muller DC, Severi G, Baglietto L, Krishnan K, English DR, Hopper JL, et al. Dietary Patterns and Prostate Cancer Risk. *Cancer Epidemiol Biomarkers Prev* (2009) 18(11):3126-9. doi: 10.1158/1055-9965.EPI-09-0780.
269. Nanri A, Shimazu T, Takachi R, Ishihara J, Mizoue T, Noda M, et al. Dietary Patterns and Type 2 Diabetes in Japanese Men and Women: The Japan Public Health Center-Based Prospective Study. *Eur J Clin Nutr* (2013) 67(1):18-24. doi: 10.1038/ejcn.2012.171.
270. Neelakantan N, Naidoo N, Koh WP, Yuan JM, van Dam RM. The Alternative Healthy Eating Index Is Associated with a Lower Risk of Fatal and Nonfatal Acute Myocardial Infarction in a Chinese Adult Population. *J Nutr* (2016) 146(7):1379-86. doi: 10.3945/jn.116.231605.
271. Neelakantan N, Koh W, Yuan J, Dam RMv. Diet-Quality Indexes Are Associated with a Lower Risk of Cardiovascular, Respiratory, and All-Cause Mortality among Chinese Adults. *J Nutr* (2018) 148(8):1323-32. doi: 10.1093/jn/nxy094.
272. Nettleton JA, Polak JF, Tracy R, Burke GL, Jacobs DR, Jr. Dietary Patterns and Incident Cardiovascular Disease in the Multi-Ethnic Study of Atherosclerosis. *Am J Clin Nutr* (2009) 90(3):647-54. doi: 10.3945/ajcn.2009.27597.
273. Neufcourt L, Assmann KE, Fezeu LK, Touvier M, Graffouillere L, Shivappa N, et al. Prospective Association between the Dietary Inflammatory Index and Metabolic Syndrome: Findings from the Su.Vi.Max Study. *Nutr Metab Cardiovasc Dis* (2015) 25(11):988-96. doi: 10.1016/j.numecd.2015.09.002.
274. Newby PK, Weismayer C, Åkesson A, Tucker KL, Wolk A. Longitudinal Changes in Food Patterns Predict Changes in Weight and Body Mass Index and the Effects Are Greatest in Obese Women. *J Nutr* (2006) 136(10):2580-7. doi: 10.1093/jn/136.10.2580.
275. Nimptsch K, Malik VS, Fung TT, Pischon T, Hu FB, Willett WC, et al. Dietary Patterns During High School and Risk of Colorectal Adenoma in a Cohort of Middle-Aged Women. *Int J Cancer* (2014) 134(10):2458-67. doi: 10.1002/ijc.28578.
276. Niu K, Momma H, Kobayashi Y, Guan L, Chujo M, Otomo A, et al. The Traditional Japanese Dietary Pattern and Longitudinal Changes in Cardiovascular Disease Risk Factors in Apparently Healthy Japanese Adults. *Eur J Nutr* (2016) 55(1):267-79. doi: 10.1007/s00394-015-0844-y.
277. Nobbs HM, Yaxley A, Thomas J, Delaney C, Koczwara B, Luszcz M, et al. Do Dietary Patterns in Older Age Influence the Development of Cancer and Cardiovascular Disease: A Longitudinal Study of Ageing. *Clin Nutr* (2016) 35(2):528-35. doi: 10.1016/j.clnu.2015.04.003.
278. Nomura SJO, Dash C, Rosenberg L, Yu J, Palmer JR, Adams-Campbell LL. Is Adherence to Diet, Physical Activity, and Body Weight Cancer Prevention Recommendations Associated with Colorectal Cancer Incidence in African American Women? *Cancer Causes Control* (2016) 27(7):869-79. doi: 10.1007/s10552-016-0760-3.
279. Nomura SJO, Dash C, Rosenberg L, Yu J, Palmer JR, Adams-Campbell LL. Adherence to Diet, Physical Activity and Body Weight Recommendations and Breast Cancer Incidence in the Black Women's Health Study. *Int J Cancer* (2016) 139(12):2738-52. doi: 10.1002/ijc.30410.

280. Northstone K, Joinson C, Emmett P, Ness A, Paus T. Are Dietary Patterns in Childhood Associated with Iq at 8 Years of Age? A Population-Based Cohort Study. *J Epidemiol Community Health* (2012) 66(7):624-8. doi: 10.1136/jech.2010.111955.
281. Nöthlings U, Murphy SP, Wilkens LR, Boeing H, Schulze MB, Bueno-de-Mesquita HB, et al. A Food Pattern That Is Predictive of Flavonol Intake and Risk of Pancreatic Cancer. *Am J Clin Nutr* (2008) 88(6):1653-62. doi: 10.3945/ajcn.2008.26398.
282. Oddy WH, Allen KL, Trapp GSA, Ambrosini GL, Black LJ, Huang R, et al. Dietary Patterns, Body Mass Index and Inflammation: Pathways to Depression and Mental Health Problems in Adolescents. *Brain Behav Immun* (2018) 69:428-39. doi: 10.1016/j.bbi.2018.01.002.
283. Odegaard AO, Koh WP, Yuan JM, Gross MD, Pereira MA. Dietary Patterns and Mortality in a Chinese Population. *Am J Clin Nutr* (2014) 100(3):877-83. doi: 10.3945/ajcn.114.086124.
284. Ogilvie RP, Lutsey PL, Heiss G, Folsom AR, Steffen LM. Dietary Intake and Peripheral Arterial Disease Incidence in Middle-Aged Adults: The Atherosclerosis Risk in Communities (Aric) Study. *Am J Clin Nutr* (2017) 105(3):651-9. doi: 10.3945/ajcn.116.137497.
285. Okada E, Nakamura K, Ukawa S, Sakata K, Date C, Iso H, et al. Dietary Patterns and Risk of Esophageal Cancer Mortality: The Japan Collaborative Cohort Study. *Nutr Cancer* (2016) 68(6):1001-9. doi: 10.1080/01635581.2016.1192202.
286. Okubo H, Crozier SR, Harvey NC, Godfrey KM, Inskip HM, Cooper C, et al. Diet Quality across Early Childhood and Adiposity at 6 Years: The Southampton Women's Survey. *Int J Obes* (2015) 39(10):1456-62. doi: 10.1038/ijo.2015.97.
287. Olsen A, Egeberg R, Halkjær J, Christensen J, Overvad K, Tjønneland A. Healthy Aspects of the Nordic Diet Are Related to Lower Total Mortality. *J Nutr* (2011) 141(4):639-44. doi: 10.3945/jn.110.131375.
288. Orchard T, Yildiz V, Steck SE, Hébert JR, Ma Y, Cauley JA, et al. Dietary Inflammatory Index, Bone Mineral Density, and Risk of Fracture in Postmenopausal Women: Results from the Women's Health Initiative. *J Bone Miner Res* (2017) 32(5):1136-46. doi: 10.1002/jbmr.3070.
289. Osler M, Andreasen AH, Heitmann B, Høidrup S, Gerdes U, Mørch Jøgensen L, et al. Food Intake Patterns and Risk of Coronary Heart Disease: A Prospective Cohort Study Examining the Use of Traditional Scoring Techniques. *Eur J Clin Nutr* (2002) 56(7):568-74. doi: 10.1038/sj.ejcn.1601360.
290. Osler M, Heitmann BL, Gerdes LU, Jørgensen LM, Schroll M. Dietary Patterns and Mortality in Danish Men and Women: A Prospective Observational Study. *Br J Nutr* (2001) 85(2):219-25. doi: 10.1079/BJN2000240.
291. Osler M, Heitmann BL, Høidrup S, Jørgensen LM, Schroll M. Food Intake Patterns, Self Rated Health and Mortality in Danish Men and Women. A Prospective Observational Study. *J Epidemiol Community Health* (2001) 55(6):399-403. doi: 10.1136/jech.55.6.399.
292. Osler M, Schroll M. Diet and Mortality in a Cohort of Elderly People in a North European Community. *Int J Epidemiol* (1997) 26(1):155-9. doi: 10.1093/ije/26.1.155.
293. Pachucki MA. Food Pattern Analysis over Time: Unhealthful Eating Trajectories Predict Obesity. *Int J Obes* (2012) 36(5):686-94. doi: 10.1038/ijo.2011.133.
294. Pala V, Lissner L, Hebestreit A, Lanfer A, Sieri S, Siani A, et al. Dietary Patterns and Longitudinal Change in Body Mass in European Children: A Follow-up Study on the Idefics Multicenter Cohort. *Eur J Clin Nutr* (2013) 67(10):1042-9. doi: 10.1038/ejcn.2013.145.
295. Panagiotakos DB, Georgousopoulou EN, Georgiopoulos GA, Pitsavos C, Chrysohoou C, Skoumas I, et al. Adherence to Mediterranean Diet Offers an Additive Protection over the Use of Statin Therapy: Results from the Attica Study (2002-2012). *Curr Vasc Pharmacol* (2015) 13(6):778-87. doi: 10.2174/1570161113666150416124957.

296. Panizza CE, Shvetsov YB, Harmon BE, Wilkens LR, Le Marchand L, Haiman C, et al. Testing the Predictive Validity of the Healthy Eating Index-2015 in the Multiethnic Cohort: Is the Score Associated with a Reduced Risk of All-Cause and Cause-Specific Mortality? *Nutrients* (2018). doi: 10.3390/nu10040452.
297. Park S, Boushey CJ, Wilkens LR, Haiman CA, Marchand Ll. High-Quality Diets Associate with Reduced Risk of Colorectal Cancer: Analyses of Diet Quality Indexes in the Multiethnic Cohort. *Gastroenterology* (2017) 153(2):386-94.e2. doi: 10.1053/j.gastro.2017.04.004.
298. Park YM, Steck SE, Fung TT, Zhang J, Hazlett LJ, Han K, et al. Mediterranean Diet and Mortality Risk in Metabolically Healthy Obese and Metabolically Unhealthy Obese Phenotypes. *Int J Obes* (2016) 40(10):1541-9. doi: 10.1038/ijo.2016.114.
299. Pastorino S, Richards M, Pierce M, Ambrosini GL. A High-Fat, High-Glycaemic Index, Low-Fibre Dietary Pattern Is Prospectively Associated with Type 2 Diabetes in a British Birth Cohort. *Br J Nutr* (2016) 115(9):1632-42. doi: 10.1017/S0007114516000672.
300. Paterson KE, Myint PK, Jennings A, Bain LKM, Lentjes MAH, Khaw K, et al. Mediterranean Diet Reduces Risk of Incident Stroke in a Population with Varying Cardiovascular Disease Risk Profiles. *Stroke* (2018) 49(10):2415-20. doi: 10.1161/ STROKEAHA.117.020258.
301. Petimar J, Smith-Warner SA, Fung TT, Rosner B, Chan AT, Hu FB, et al. Recommendation-Based Dietary Indexes and Risk of Colorectal Cancer in the Nurses' Health Study and Health Professionals Follow-up Study. *Am J Clin Nutr* (2018) 108(5):1092-103. doi: 10.1093/ajcn/nqy171.
302. Pham TM, Fujino Y, Kikuchi S, Tamakoshi A, Matsuda S, Yoshimura T. Dietary Patterns and Risk of Stomach Cancer Mortality: The Japan Collaborative Cohort Study. *Ann Epidemiol* (2010) 20(5):356-63. doi: 10.1016/j.annepidem.2010.02.002.
303. Pilleron S, Ajana S, Jutand MA, Helmer C, Dartigues JF, Samieri C, et al. Dietary Patterns and 12-Year Risk of Frailty: Results from the Three-City Bordeaux Study. *J Am Med Dir Assoc* (2017) 18(2):169-75. doi: 10.1016/j.jamda.2016.09.014.
304. Pimenta AM, Toledo E, Rodriguez-Diez MC, Gea A, Lopez-Iracheta R, Shivappa N, et al. Dietary Indexes, Food Patterns and Incidence of Metabolic Syndrome in a Mediterranean Cohort: The Sun Project. *Clin Nutr* (2015) 34(3):508-14. doi: 10.1016/j.clnu.2014.06.002.
305. Ping-Delfos WLCS, Beilin LJ, Oddy WH, Burrows S, Mori TA. Use of the Dietary Guideline Index to Assess Cardiometabolic Risk in Adolescents. *Br J Nutr* (2015) 113(11):1741-52. doi: 10.1017/S0007114515001026.
306. Prinelli F, Yannakoulia M, Anastasiou CA, Adorni F, Santo SGd, Musicco M, et al. Mediterranean Diet and Other Lifestyle Factors in Relation to 20-Year All-Cause Mortality: A Cohort Study in an Italian Population. *Br J Nutr* (2015) 113(6):1003-11. doi: 10.1017/S0007114515000318.
307. Qi L, Cornelis MC, Zhang CL, Dam RMv, Hu FB. Genetic Predisposition, Western Dietary Pattern, and the Risk of Type 2 Diabetes in Men. *Am J Clin Nutr* (2009) 89(5):1453-8. doi: 10.3945/ajcn.2008.27249.
308. Quatromoni PA, Copenhafer DL, D'Agostino RB, Millen BE. Dietary Patterns Predict the Development of Overweight in Women: The Framingham Nutrition Studies. *J Am Diet Assoc* (2002) 102(9):1240-6. doi: 10.1016/s0002-8223(02)90275-0.
309. Rahi B, Ajana S, Tabue-Teguo M, Dartigues JF, Peres K, Feart C. High Adherence to a Mediterranean Diet and Lower Risk of Frailty among French Older Adults Community-Dwellers: Results from the Three-City-Bordeaux Study. *Clin Nutr* (2018) 37(4):1293-8. doi: 10.1016/j.clnu.2017.05.020.
310. Rajaobelina K, Dow C, Romana Mancini F, Dartois L, Boutron-Ruault MC, Balkau B, et al. Population Attributable Fractions of the Main Type 2 Diabetes Mellitus Risk Factors in Women:

Findings from the French E3n Cohort. *J Diabetes* (2019) 11(3):242-53. doi: 10.1111/1753-0407.12839.
311. Rashidkhani B, Åkesson A, Lindblad P, Wolk A. Major Dietary Patterns and Risk of Renal Cell Carcinoma in a Prospective Cohort of Swedish Women. *J Nutr* (2005) 135(7):1757-62. doi: 10.1093/jn/135.7.1757.
312. Reedy J, Wirfält E, Flood A, Mitrou PN, Krebs-Smith SM, Kipnis V, et al. Comparing 3 Dietary Pattern Methods-Cluster Analysis, Factor Analysis, and Index Analysis-with Colorectal Cancer Risk. *Am J Epidemiol* (2010) 171(4):479-87. doi: 10.1093/aje/kwp393.
313. Ritchie LD, Spector P, Stevens MJ, Schmidt MM, Schreiber GB, Striegel-Moore RH, et al. Dietary Patterns in Adolescence Are Related to Adiposity in Young Adulthood in Black and White Females. *J Nutr* (2007) 137(2):399-406. doi: 10.1093/jn/137.2.399.
314. Rogers TS, Harrison S, Judd S, Orwoll ES, Marshall LM, Shannon J, et al. Dietary Patterns and Longitudinal Change in Hip Bone Mineral Density among Older Men. *Osteoporos Int* (2018) 29(5):1135-45. doi: 10.1007/s00198-018-4388-x.
315. Romaguera D. Mediterranean Diet and Type 2 Diabetes Risk in the European Prospective Investigation into Cancer and Nutrition (Epic) Study: The Interact Project. *Diabetes Care* (2011) 34(9):1913-8. doi: 10.2337/dc11-0891.
316. Romaguera D, Norat T, Vergnaud AC, Mouw T, May AM, Agudo A, et al. Mediterranean Dietary Patterns and Prospective Weight Change in Participants of the Epic-Panacea Project. *Am J Clin Nutr* (2010) 92(4):912-21. doi: 10.3945/ajcn.2010.29482.
317. Rossi M, Turati F, Lagiou P, Trichopoulos D, Augustin LS, Vecchia Cl, et al. Mediterranean Diet and Glycaemic Load in Relation to Incidence of Type 2 Diabetes: Results from the Greek Cohort of the Population-Based European Prospective Investigation into Cancer and Nutrition (Epic). *Diabetologia* (2013) 56(11):2405-13. doi: 10.1007/s00125-013-3013-y.
318. Roswall N, Ängquist L, Ahluwalia TS, Romaguera D, Larsen SC, Østergaard JN, et al. Association between Mediterranean and Nordic Diet Scores and Changes in Weight and Waist Circumference: Influence of Fto and Tcf7l2 Loci. *Am J Clin Nutr* (2014) 100(4):1188-97. doi: 10.3945/ajcn.114.089706.
319. Roswall N, Sandin S, Löf M, Skeie G, Olsen A, Adami HO, et al. Adherence to the Healthy Nordic Food Index and Total and Cause-Specific Mortality among Swedish Women. *Eur J Epidemiol* (2015) 30(6):509-17. doi: 10.1007/s10654-015-0021-x.
320. Roswall N, Sandin S, Scragg R, Löf M, Skeie G, Olsen A, et al. No Association between Adherence to the Healthy Nordic Food Index and Cardiovascular Disease Amongst Swedish Women: A Cohort Study. *J Intern Med* (2015) 278(5):531-41. doi: 10.1111/joim.12378.
321. Rumawas ME, Meigs JB, Dwyer JT, McKeown NM, Jacques PF. Mediterranean-Style Dietary Pattern, Reduced Risk of Metabolic Syndrome Traits, and Incidence in the Framingham Offspring Cohort. *Am J Clin Nutr* (2009) 90(6):1608-14. doi: 10.3945/ajcn.2009.27908.
322. Rutten-Jacobs LC, Larsson SC, Malik R, Rannikmäe K, Sudlow CL, Dichgans M, et al. Genetic Risk, Incident Stroke, and the Benefits of Adhering to a Healthy Lifestyle: Cohort Study of 306 473 Uk Biobank Participants. *BMJ* (2018) 363. doi: 10.1136/bmj.k4168.
323. Sadeghi M, Talaei M, Parvaresh rizi EP, Dianatkhah M, Oveisgharan S, Sarrafzadegan N. Determinants of Incident Prediabetes and Type 2 Diabetes in a 7-Year Cohort in a Developing Country: The Isfahan Cohort Study. *J Diabetes* (2015) 7(5):633-41. doi: 10.1111/1753-0407.12236.
324. Saldanha-Gomes C, Heude B, Charles MA, Lauzon-Guillain Bd, Botton J, Carles S, et al. Prospective Associations between Energy Balance-Related Behaviors at 2 Years of Age and Subsequent Adiposity: The Eden Mother-Child Cohort. *Int J Obes* (2017) 41(1):38-45. doi: 10.1038/ijo.2016.138.

325. Sánchez-Villegas A, Bes-Rastrollo M, Martínez-González M, Serra-Majem L. Adherence to a Mediterranean Dietary Pattern and Weight Gain in a Follow-up Study: The Sun Cohort. *Int J Obes* (2006) 30(2):350-8. doi: 10.1038/sj.ijo.0803118.
326. Satija A, Bhupathiraju SN, Rimm EB, Spiegelman D, Chiuve SE, Borgi L, et al. Plant-Based Dietary Patterns and Incidence of Type 2 Diabetes in Us Men and Women: Results from Three Prospective Cohort Studies. *PLoS Med* (2016) 13(6). doi: 10.1371/journal.pmed.1002039.
327. Schneider BC, Dumith Sde C, Lopes C, Severo M, Assuncao MC. How Do Tracking and Changes in Dietary Pattern During Adolescence Relate to the Amount of Body Fat in Early Adulthood? *PLoS ONE* (2016) 11(2):e0149299. doi: 10.1371/journal.pone.0149299.
328. Schulpen M, Van Den Brandt PA. Adherence to the Mediterranean Diet and Risk of Lung Cancer in the Netherlands Cohort Study. *Br J Nutr* (2018) 119(6):674-84. doi: 10.1017/S0007114517003737.
329. Schulpen M, Peeters PH, van den Brandt PA. Mediterranean Diet Adherence and Risk of Esophageal and Gastric Cancer Subtypes in the Netherlands Cohort Study. *Gastric Cancer* (2019) 22:663-74. doi: 10.1007/s10120-019-00927-x.
330. Schulpen M, Peeters PH, van den Brandt PA. Mediterranean Diet Adherence and Risk of Pancreatic Cancer: A Pooled Analysis of Two Dutch Cohorts. *Int J Cancer* (2019) 144(7):1550-60. doi: 10.1002/ijc.31872.
331. Schulz M, Hoffmann K, Weikert C, Nöthlings U, Schulze MB, Boeing H. Identification of a Dietary Pattern Characterized by High-Fat Food Choices Associated with Increased Risk of Breast Cancer: The European Prospective Investigation into Cancer and Nutrition (Epic)-Potsdam Study. *Br J Nutr* (2008) 100(5):942-6. doi: 10.1017/S0007114508966149.
332. Schulz M, Nöthlings U, Hoffmann K, Bergmann MM, Boeing H. Identification of a Food Pattern Characterized by High-Fiber and Low-Fat Food Choices Associated with Low Prospective Weight Change in the Epic-Potsdam Cohort. *J Nutr* (2005) 135(5):1183-9. doi: 10.1093/jn/135.5.1183.
333. Schulze MB, Hoffmann K, Manson JE, Willett WC, Meigs JB, Weikert C, et al. Dietary Pattern, Inflammation, and Incidence of Type 2 Diabetes in Women. *Am J Clin Nutr* (2005) 82(3):675-84. doi: 10.1093/ajcn.82.3.675.
334. Seino F, Date C, Nakayama T, Yoshiike N, Yokoyama T, Yamaguchi M, et al. Dietary Lipids and Incidence of Cerebral Infarction in a Japanese Rural Community. *J Nutr Sci Vitaminol* (1997) 43(1):83-99. doi: 10.3177/jnsv.43.83.
335. Seymour JD, Calle EE, Flagg EW, Coates RJ, Ford ES, Thun MJ. Diet Quality Index as a Predictor of Short-Term Mortality in the American Cancer Society Cancer Prevention Study Ii Nutrition Cohort. *Am J Epidemiol* (2003) 157(11):980-8. doi: 10.1093/aje/kwg077.
336. Shah NS, Leonard D, Finley CE, Rodriguez F, Sarraju A, Barlow CE, et al. Dietary Patterns and Long-Term Survival: A Retrospective Study of Healthy Primary Care Patients. *Am J Med* (2018) 131(1):48-55. doi: 10.1016/j.amjmed.2017.08.010.
337. Shan Z, Li Y, Zong G, Guo Y, Li J, Manson JE, et al. Rotating Night Shift Work and Adherence to Unhealthy Lifestyle in Predicting Risk of Type 2 Diabetes: Results from Two Large Us Cohorts of Female Nurses. *BMJ* (2018) 363. doi: 10.1136/bmj.k4641.
338. Shaw SC, Parsons CM, Fuggle NR, Edwards MH, Robinson SM, Dennison EM, et al. Diet Quality and Bone Measurements Using Hrpqct and Pqct in Older Community-Dwelling Adults from the Hertfordshire Cohort Study. *Calcif Tissue Int* (2018):1-7. doi: 10.1007/s00223-018-0445-x.
339. Sherafat-Kazemzadeh R, Egtesadi S, Mirmiran P, Gohari M, Farahani SJ, Esfahani FH, et al. Dietary Patterns by Reduced Rank Regression Predicting Changes in Obesity Indices in a Cohort Study: Tehran Lipid and Glucose Study. *Asia Pac J Clin Nutr* (2010) 19(1):22-32.

340. Shi L, Brunius C, Johansson I, Bergdahl IA, Lindahl B, Hanhineva K, et al. Plasma Metabolites Associated with Healthy Nordic Dietary Indexes and Risk of Type 2 Diabetes - a Nested Case-Control Study in a Swedish Population. *Am J Clin Nutr* (2018) 108(3):564-75. doi: 10.1093/ajcn/nqy145.
341. Shimazu T, Kuriyama S, Hozawa A, Ohmori K, Sato Y, Nakaya N, et al. Dietary Patterns and Cardiovascular Disease Mortality in Japan: A Prospective Cohort Study. *Int J Epidemiol* (2007) 36(3):600-9. doi: 10.1093/ije/dym005.
342. Shin S, Saito E, Sawada N, Ishihara J, Takachi R, Nanri A, et al. Dietary Patterns and Colorectal Cancer Risk in Middle-Aged Adults: A Large Population-Based Prospective Cohort Study. *Clin Nutr* (2018) 37(3):1019-26. doi: 10.1016/j.clnu.2017.04.015.
343. Shin S, Saito E, Inoue M, Sawada N, Ishihara J, Takachi R, et al. Dietary Pattern and Breast Cancer Risk in Japanese Women: The Japan Public Health Center-Based Prospective Study (Jphc Study). *Br J Nutr* (2016) 115(10):1769-79. doi: 10.1017/S0007114516000684.
344. Shin S, Saito E, Sawada N, Ishihara J, Takachi R, Nanri A, et al. Dietary Patterns and Prostate Cancer Risk in Japanese: The Japan Public Health Center-Based Prospective Study (Jphc Study). *Cancer Causes Control* (2018) 29(6):589-600. doi: 10.1007/s10552-018-1030-3.
345. Shivappa N, Hebert JR, Kivimaki M, Akbaraly T. Alternative Healthy Eating Index 2010, Dietary Inflammatory Index and Risk of Mortality: Results from the Whitehall Ii Cohort Study and Meta-Analysis of Previous Dietary Inflammatory Index and Mortality Studies. *Br J Nutr* (2017) 118(3):210-21. doi: 10.1017/S0007114517001908.
346. Shvetsov YB, Harmon BE, Ettienne R, Wilkens LR, Marchand Ll, Kolonel LN, et al. The Influence of Energy Standardisation on the Alternate Mediterranean Diet Score and Its Association with Mortality in the Multiethnic Cohort. *Br J Nutr* (2016) 116(9):1592-601. doi: 10.1017/S0007114516003482.
347. Sieri S, Krogh V, Pala V, Muti P, Micheli A, Evangelista A, et al. Dietary Patterns and Risk of Breast Cancer in the Ordet Cohort. *Cancer Epidemiol Biomarkers Prev* (2004) 13(4):567-72.
348. Sijtsma FP, Soedamah-Muthu SS, de Hoon SE, Jacobs DR, Jr., Kromhout D. Healthy Eating and Survival among Elderly Men with and without Cardiovascular-Metabolic Diseases. *Nutr Metab Cardiovasc Dis* (2015) 25(12):1117-24. doi: 10.1016/j.numecd.2015.08.008.
349. Sjögren P, Becker W, Warensjö E, Olsson E, Byberg L, Gustafsson IB, et al. Mediterranean and Carbohydrate-Restricted Diets and Mortality among Elderly Men: A Cohort Study in Sweden. *Am J Clin Nutr* (2010) 92(4):967-74. doi: 10.3945/ajcn.2010.29345.
350. Solbak NM, Xu JY, Vena JE, Csizmadi I, Whelan HK, Robson PJ. Diet Quality Is Associated with Reduced Incidence of Cancer and Self-Reported Chronic Disease: Observations from Alberta's Tomorrow Project. *Prev Med* (2017) 101:178-87. doi: 10.1016/j.ypmed.2017.06.009.
351. Sotos-Prieto M, Bhupathiraju SN, Mattei J, Fung TT, Li Y, Pan A, et al. Changes in Diet Quality Scores and Risk of Cardiovascular Disease among Us Men and Women. *Circulation* (2015) 132(23):2212-9. doi: 10.1161/CIRCULATIONAHA.115.017158.
352. Steffen LM, Van Horn L, Daviglus ML, Zhou X, Reis JP, Loria CM, et al. A Modified Mediterranean Diet Score Is Associated with a Lower Risk of Incident Metabolic Syndrome over 25 Years among Young Adults: The Cardia (Coronary Artery Risk Development in Young Adults) Study. *Br J Nutr* (2014) 112(10):1654-61. doi: 10.1017/S0007114514002633.
353. Stefler D, Malyutina S, Kubinova R, Pajak A, Peasey A, Pikhart H, et al. Mediterranean Diet Score and Total and Cardiovascular Mortality in Eastern Europe: The Hapiee Study. *Eur J Nutr* (2017) 56(1):421-9. doi: 10.1007/s00394-015-1092-x.

354. Stefler D, Pikhart H, Jankovic N, Kubinova R, Pajak A, Malyutina S, et al. Healthy Diet Indicator and Mortality in Eastern European Populations: Prospective Evidence from the Hapiee Cohort. *Eur J Clin Nutr* (2014) 68(12):1346-52. doi: 10.1038/ejcn.2014.134.
355. Stricker MD, Onland-Moret NC, Boer JM, van der Schouw YT, Verschuren WM, May AM, et al. Dietary Patterns Derived from Principal Component- and K-Means Cluster Analysis: Long-Term Association with Coronary Heart Disease and Stroke. *Nutr Metab Cardiovasc Dis* (2013) 23(3):250-6. doi: 10.1016/j.numecd.2012.02.006.
356. Struijk EA, May AM, Wezenbeek NLW, Fransen HP, Soedamah-Muthu SS, Geelen A, et al. Adherence to Dietary Guidelines and Cardiovascular Disease Risk in the Epic-Nl Cohort. *Int J Cardiol* (2014) 176(2):354-9. doi: 10.1016/j.ijcard.2014.07.017.
357. Suwaidi JA. Dietary Patterns and Their Association with Acute Coronary Heart Disease: Lessons from the Regards Study. *Glob Cardiol Sci Pract* (2015) 2015(4). doi: 10.5339/gcsp.2015.56.
358. Tabung FK, Liu L, Wang W, Fung TT, Wu K, Smith-Warner SA, et al. Association of Dietary Inflammatory Potential with Colorectal Cancer Risk in Men and Women. *JAMA Oncol* (2018) 4(3):366-73. doi: 10.1001/jamaoncol.2017.4844.
359. Tabung FK, Wang W, Fung TT, Smith-Warner SA, Keum N, Wu K, et al. Association of Dietary Insulinemic Potential and Colorectal Cancer Risk in Men and Women. *Am J Clin Nutr* (2018) 108(2):363-70. doi: 10.1093/ajcn/nqy093.
360. Tektonidis TG, Åkesson A, Gigante B, Wolk A, Larsson SC. Adherence to a Mediterranean Diet Is Associated with Reduced Risk of Heart Failure in Men. *Eur J Heart Fail* (2016) 18(3):253-9. doi: 10.1002/ejhf.481.
361. Tektonidis TG, Åkesson A, Gigante B, Wolk A, Larsson SC. A Mediterranean Diet and Risk of Myocardial Infarction, Heart Failure and Stroke: A Population-Based Cohort Study. *Atherosclerosis* (2015) 243(1):93-8. doi: 10.1016/j.atherosclerosis.2015.08.039.
362. Terry P, Hu FB, Hansen H, Wolk A. Prospective Study of Major Dietary Patterns and Colorectal Cancer Risk in Women. *Am J Epidemiol* (2001) 154(12):1143-9. doi: 10.1093/aje/154.12.1143.
363. Tharrey M, Mariotti F, Mashchak A, Barbillon P, Delattre M, Fraser GE. Patterns of Plant and Animal Protein Intake Are Strongly Associated with Cardiovascular Mortality: The Adventist Health Study-2 Cohort. *Int J Epidemiol* (2018) 47(5):1603-12. doi: 10.1093/ije/dyy030.
364. Tikk K, Sookthai D, Monni S, Gross ML, Lichy C, Kloss M, et al. Primary Preventive Potential for Stroke by Avoidance of Major Lifestyle Risk Factors: The European Prospective Investigation into Cancer and Nutrition-Heidelberg Cohort. *Stroke* (2014) 45(7):2041-6. doi: 10.1161/STROKEAHA.114.005025.
365. Tobias DK, Hu FB, Chavarro J, Rosner B, Mozaffarian D, Zhang C. Healthful Dietary Patterns and Type 2 Diabetes Mellitus Risk among Women with a History of Gestational Diabetes Mellitus. *Arch Intern Med* (2012) 172(20):1566-72. doi: 10.1001/archinternmed.2012.3747.
366. Tognon G, Hebestreit A, Lanfer A, Moreno LA, Pala V, Siani A, et al. Mediterranean Diet, Overweight and Body Composition in Children from Eight European Countries: Cross-Sectional and Prospective Results from the Idefics Study. *Nutr Metab Cardiovasc Dis* (2014) 24(2):205-13. doi: 10.1016/j.numecd.2013.04.013.
367. Tognon G, Lissner L, Sæbye D, Walker KZ, Heitmann BL. The Mediterranean Diet in Relation to Mortality and Cvd: A Danish Cohort Study. *Br J Nutr* (2014) 111(1):151-9. doi: 10.1017/S0007114513001931.
368. Tong TYN, Wareham NJ, Khaw KT, Imamura F, Forouhi NG. Prospective Association of the Mediterranean Diet with Cardiovascular Disease Incidence and Mortality and Its Population Impact

in a Non-Mediterranean Population: The Epic-Norfolk Study. *BMC Med* (2016) 14(1). doi: 10.1186/s12916-016-0677-4.
369. Tortosa A, Bes-Rastrollo M, Sanchez-Villegas A, Basterra-Gortari FJ, Nuñez-Cordoba JM, Martinez-Gonzalez MA. Mediterranean Diet Inversely Associated with the Incidence of Metabolic Syndrome: The Sun Prospective Cohort. *Diabetes Care* (2007) 30(11):2957-9. doi: 10.2337/dc07-1231.
370. Trichopoulou A. Modified Mediterranean Diet and Survival: Epic-Elderly Prospective Cohort Study. *BMJ* (2005) 330(7498):991-5. doi: 10.1136/bmj.38415.644155.8F.
371. Trichopoulou A, Bamia C, Lagiou P, Trichopoulos D. Conformity to Traditional Mediterranean Diet and Breast Cancer Risk in the Greek Epic (European Prospective Investigation into Cancer and Nutrition) Cohort. *Am J Clin Nutr* (2010) 92(3):620-5. doi: 10.3945/ajcn.2010.29619.
372. Trichopoulou A, Bamia C, Trichopoulos D. Anatomy of Health Effects of Mediterranean Diet: Greek Epic Prospective Cohort Study. *BMJ* (2009) 339(7711):26-8. doi: 10.1136/bmj.b2337.
373. Trichopoulou A, Kouris-Blazos A, Wahlqvist ML, Gnardellis C, Lagiou P, Polychronopoulos E, et al. Diet and Overall Survival in Elderly People. *BMJ* (1995) 311(7018):1457-60. doi: 10.1136/bmj.311.7018.1457.
374. Tseng M, Breslow RA, DeVellis RF, Ziegler RG. Dietary Patterns and Prostate Cancer Risk in the National Health and Nutrition Examination Survey Epidemiological Follow-up Study Cohort. *Cancer Epidemiol Biomarkers Prev* (2004) 13(1):71-7. doi: 10.1158/1055-9965.EPI-03-0076.
375. Tsivgoulis G, Psaltopoulou T, Wadley VG, Alexandrov AV, Howard G, Unverzagt FW, et al. Adherence to a Mediterranean Diet and Prediction of Incident Stroke. *Stroke* (2015) 46(3):780-5. doi: 10.1161/STROKEAHA.114.007894.
376. Turati F, Dilis V, Rossi M, Lagiou P, Benetou V, Katsoulis M, et al. Glycemic Load and Coronary Heart Disease in a Mediterranean Population: The Epic Greek Cohort Study. *Nutr Metab Cardiovasc Dis* (2015) 25(3):336-42. doi: 10.1016/j.numecd.2014.12.002.
377. van Dam RM, Rimm EB, Willett WC, Stampfer MJ, Hu FB. Dietary Patterns and Risk for Type 2 Diabetes Mellitus in U.S. Men. *Ann Intern Med* (2002) 136(3):201-9. doi: 10.7326/0003-4819-136-3-200202050-00008.
378. Van Dam RM, Li T, Spiegelman D, Franco OH, Hu FB. Combined Impact of Lifestyle Factors on Mortality: Prospective Cohort Study in Us Women. *BMJ* (2008) 337(7672):742-5. doi: 10.1136/bmj.a1440.
379. van de Laar RJ, Stehouwer CD, van Bussel BC, Prins MH, Twisk JW, Ferreira I. Adherence to a Mediterranean Dietary Pattern in Early Life Is Associated with Lower Arterial Stiffness in Adulthood: The Amsterdam Growth and Health Longitudinal Study. *J Intern Med* (2013) 273(1):79-93. doi: 10.1111/j.1365-2796.2012.02577.x.
380. van den Brandt PA. The Impact of a Mediterranean Diet and Healthy Lifestyle on Premature Mortality in Men and Women. *Am J Clin Nutr* (2011) 94(3):913-20. doi: 10.3945/ajcn.110.008250.
381. van den Brandt PA, Schulpen M. Mediterranean Diet Adherence and Risk of Postmenopausal Breast Cancer: Results of a Cohort Study and Meta-Analysis. *Int J Cancer* (2017) 140(10):2220-31. doi: 10.1002/ijc.30654.
382. Van Den Hooven EH, Ambrosini GL, Huang RC, Mountain J, Straker L, Walsh JP, et al. Identification of a Dietary Pattern Prospectively Associated with Bone Mass in Australian Young Adults. *Am J Clin Nutr* (2015) 102(5):1035-43. doi: 10.3945/ajcn.115.110502.
383. Horn Lv, Tian L, Neuhouser ML, Howard BV, Eaton CB, Snetselaar L, et al. Dietary Patterns Are Associated with Disease Risk among Participants in the Women's Health Initiative Observational Study. *J Nutr* (2012) 142(2):284-91. doi: 10.3945/jn.111.145375.

384. Vargas AJ, Neuhouser ML, George SM, Thomson CA, Ho GYF, Rohan TE, et al. Diet Quality and Colorectal Cancer Risk in the Women's Health Initiative Observational Study. *Am J Epidemiol* (2016) 184(1):23-32. doi: 10.1093/aje/kwv304.
385. Veglia F, Baldassarre D, de Faire U, Kurl S, Smit AJ, Rauramaa R, et al. A Priori-Defined Mediterranean-Like Dietary Pattern Predicts Cardiovascular Events Better in North Europe Than in Mediterranean Countries. *Int J Cardiol* (2019) 282:88-92. doi: 10.1016/j.ijcard.2018.11.124.
386. Velie EM, Schairer C, Flood A, He JP, Khattree R, Schatzkin A. Empirically Derived Dietary Patterns and Risk of Postmenopausal Breast Cancer in a Large Prospective Cohort Study. *Am J Clin Nutr* (2005) 82(6):1308-19. doi: 10.1093/ajcn/82.6.1308.
387. Voortman T, Kiefte-de Jong JC, Ikram MA, Stricker BH, van Rooij FJA, Lahousse L, et al. Adherence to the 2015 Dutch Dietary Guidelines and Risk of Non-Communicable Diseases and Mortality in the Rotterdam Study. *Eur J Epidemiol* (2017) 32(11):993-1005. doi: 10.1007/s10654-017-0295-2.
388. Wahlqvist ML, Darmadi-Blackberry I, Kouris-Blazos A, Jolley D, Steen B, Lukito W, et al. Does Diet Matter for Survival in Long-Lived Cultures? *Asia Pac J Clin Nutr* (2005) 14(1):2-6.
389. Waijers PMCM, Ocké MC, Rossum CTMv, Peeters PHM, Bamia C, Chloptsios Y, et al. Dietary Patterns and Survival in Older Dutch Women. *Am J Clin Nutr* (2006) 83(5):1170-6. doi: 10.1093/ajcn/83.5.1170.
390. Walls HL, Magliano DJ, McNeil JJ, Stevenson C, Ademi Z, Shaw J, et al. Predictors of Increasing Waist Circumference in an Australian Population. *Public Health Nutr* (2011) 14(5):870-81. doi: 10.1017/S1368980010002673.
391. Wang Z, Adair LS, Cai J, Gordon-Larsen P, Siega-Riz AM, Zhang B, et al. Diet Quality Is Linked to Insulin Resistance among Adults in China. *J Nutr* (2017) 147(11):2102-8. doi: 10.3945/jn.117.256180.
392. Wang Z, Siega-Riz AM, Gordon-Larsen P, Cai J, Adair LS, Zhang B, et al. Diet Quality and Its Association with Type 2 Diabetes and Major Cardiometabolic Risk Factors among Adults in China. *Nutr Metab Cardiovasc Dis* (2018) 28(10):987-1001. doi: 10.1016/j.numecd.2018.06.012.
393. Ward KA, Prentice A, Kuh DL, Adams JE, Ambrosini GL. Life Course Dietary Patterns and Bone Health in Later Life in a British Birth Cohort Study. *J Bone Miner Res* (2016) 31(6):1167-76. doi: 10.1002/jbmr.2798.
394. Whalen KA, Judd S, McCullough ML, Flanders WD, Hartman TJ, Bostick RM. Paleolithic and Mediterranean Diet Pattern Scores Are Inversely Associated with All-Cause and Cause-Specific Mortality in Adults. *J Nutr* (2017) 147(4):612-20. doi: 10.3945/jn.116.241919.
395. Wie GA, Cho YA, Kang H, Ryu KA, Yoo MK, Kim J, et al. Identification of Major Dietary Patterns in Korean Adults and Their Association with Cancer Risk in the Cancer Screening Examination Cohort. *Eur J Clin Nutr* (2017) 71(10):1223-9. doi: 10.1038/ejcn.2017.6.
396. Wirfält E, Midthune D, Reedy J, Mitrou P, Flood A, Subar AF, et al. Associations between Food Patterns Defined by Cluster Analysis and Colorectal Cancer Incidence in the Nih-Aarp Diet and Health Study. *Eur J Clin Nutr* (2009) 63(6):707-17. doi: 10.1038/ejcn.2008.40.
397. Witlox WJA, van Osch FHM, Brinkman M, Jochems S, Goossens ME, Weiderpass E, et al. An Inverse Association between the Mediterranean Diet and Bladder Cancer Risk: A Pooled Analysis of 13 Cohort Studies. *Eur J Nutr* (2019) 59:287-96. doi: 10.1007/s00394-019-01907-8.
398. Wolters M, Joslowski G, Plachta-Danielzik S, Standl M, Müller MJ, Ahrens W, et al. Dietary Patterns in Primary School Are of Prospective Relevance for the Development of Body Composition in Two German Pediatric Populations. *Nutrients* (2018) 10(10):1442-. doi: 10.3390/nu10101442.

399. Wu K, Hu FB, Fuchs C, Rimm EB, Willett WC, Giovannucci E. Dietary Patterns and Risk of Colon Cancer and Adenoma in a Cohort of Men (United States). *Cancer Causes Control* (2004) 15(9):853-62. doi: 10.1007/s10552-004-1809-2.
400. Wu K, Hu FB, Willett WC, Giovannucci E. Dietary Patterns and Risk of Prostate Cancer in U.S. Men. *Cancer Epidemiol Biomarkers Prev* (2006) 15(1):167-71. doi: 10.1158/1055-9965.EPI-05-0100.
401. Xie J, Poole EM, Terry KL, Fung TT, Rosner BA, Willett WC, et al. A Prospective Cohort Study of Dietary Indices and Incidence of Epithelial Ovarian Cancer. *J Ovarian Res* (2014) 7(1). doi: 10.1186/s13048-014-0112-4.
402. Yu D, Sonderman J, Buchowski MS, McLaughlin JK, Shu XO, Steinwandel M, et al. Healthy Eating and Risks of Total and Cause-Specific Death among Low-Income Populations of African-Americans and Other Adults in the Southeastern United States: A Prospective Cohort Study. *PLoS Med* (2015) 12(5). doi: 10.1371/journal.pmed.1001830.
403. Yu D, Zheng W, Cai H, Xiang YB, Li H, Gao YT, et al. Long-Term Diet Quality and Risk of Type 2 Diabetes among Urban Chinese Adults. *Diabetes Care* (2018) 41(4):723-30. doi: 10.2337/dc17-1626.
404. Yu D, Zhang X, Xiang YB, Yang G, Li H, Gao YT, et al. Adherence to Dietary Guidelines and Mortality: A Report from Prospective Cohort Studies of 134,000 Chinese Adults in Urban Shanghai. *Am J Clin Nutr* (2014) 100(2):693-700. doi: 10.3945/ajcn.113.079194.
405. Yu R, Woo J, Chan R, Sham A, Ho S, Tso A, et al. Relationship between Dietary Intake and the Development of Type 2 Diabetes in a Chinese Population: The Hong Kong Dietary Survey. *Public Health Nutr* (2011) 14(7):1133-41. doi: 10.1017/S136898001100053X.
406. Zamora D, Gordon-Larsen P, Jacobs DR, Jr., Popkin BM. Diet Quality and Weight Gain among Black and White Young Adults: The Coronary Artery Risk Development in Young Adults (Cardia) Study (1985-2005). *Am J Clin Nutr* (2010) 92(4):784-93. doi: 10.3945/ajcn.2010.29161.
407. Zazpe I, Sánchez-Tainta A, Toledo E, Sánchez-Villegas A, Martínez-González MÁ. Dietary Patterns and Total Mortality in a Mediterranean Cohort: The Sun Project. *J Acad Nutr Diet* (2014) 114(1):37-47. doi: 10.1016/j.jand.2013.07.024.
408. Zhang W, Xiang YB, Li HL, Yang G, Cai H, Ji BT, et al. Vegetable-Based Dietary Pattern and Liver Cancer Risk: Results from the Shanghai Women's and Men's Health Studies. *Cancer Sci* (2013) 104(10):1353-61. doi: 10.1111/cas.12231.
409. Zhao W, Ukawa S, Okada E, Wakai K, Kawamura T, Ando M, et al. The Associations of Dietary Patterns with All-Cause Mortality and Other Lifestyle Factors in the Elderly: An Age-Specific Prospective Cohort Study. *Clin Nutr* (2018) 38:288-96. doi: 10.1016/j.clnu.2018.01.018.
410. Zheng J, Merchant AT, Wirth MD, Zhang J, Antwi SO, Shoaibi A, et al. Inflammatory Potential of Diet and Risk of Pancreatic Cancer in the Prostate, Lung, Colorectal and Ovarian (Plco) Cancer Screening Trial. *Int J Cancer* (2018) 142(12):2461-70. doi: 10.1002/ijc.31271.
